# Causal effects of air pollutants on lung function and chronic respiratory diseases: a Mendelian randomization study

**DOI:** 10.3389/fpubh.2024.1438974

**Published:** 2024-09-09

**Authors:** Xuannian Li, Suqi Liu, Nan Jiang, Fei Xu, Huaman Liu, Xinhua Jia

**Affiliations:** ^1^The First College of Clinical Medicine, Shandong University of Traditional Chinese Medicine, Jinan, Shandong, China; ^2^Department of Geriatric Medicine, Affiliated Hospital of Shandong University of Traditional Chinese Medicine, Jinan, Shandong, China; ^3^Department of General Medicine, Affiliated Hospital of Shandong University of Traditional Chinese Medicine, Jinan, Shandong, China; ^4^Department of Pneumology and Critical Care Medicine, Affiliated Hospital of Shandong University of Traditional Chinese Medicine, Jinan, Shandong, China

**Keywords:** air pollution, Mendelian randomization, inflammatory proteins, chronic respiratory diseases, lung function

## Abstract

**Objectives:**

Our study aims to clarify the causality between air pollutants and lung function, chronic respiratory diseases, and the potential mediating effects of inflammatory proteins.

**Method:**

We employed Mendelian Randomization (MR) analysis with comprehensive instrumental variables screening criteria to investigate the effects of air pollutants on lung function and chronic lung diseases. Our study incorporated genetic instruments for air pollutants, ensuring *F*-statistics above 20.86. A total of 18 MR analyses were conducted using the inverse-variance weighted approach, along with heterogeneity and pleiotropy tests to validate the results. Mediated MR analysis was utilized to evaluate the inflammatory proteins mediating the effects of air pollutants.

**Result:**

MR analysis demonstrated significant causal interactions of particulate matter 2.5 (PM_2.5_), PM_10_, and Nitrogen dioxide (NO_2_) with lung function decline. Specifically, PM_10_ negatively affected forced expiratory volume in one second (FEV_1_) (OR: 0.934, 95% CI: 0.904–0.965, *p* = 4.27 × 10^−5^), forced vital capacity (FVC) (OR: 0.941, 95% CI: 0.910–0.972, *p* = 2.86 × 10^−4^), and FEV_1_/FVC (OR: 0.965, 95% CI: 0.934–0.998, *p* = 0.036). PM_2.5_ and NO_2_ were identified as potential risk factors for impairing FEV_1_ (OR: 0.936, 95% CI: 0.879–0.998, *p* = 0.042) and FEV_1_/FVC (OR: 0.943, 95% CI: 0.896–0.992, *p* = 0.024), respectively. For chronic respiratory diseases, PM_2.5_ and NO_2_ were associated with increased COPD incidence (OR: 1.273, 95% CI: 1.053–1.541, *p* = 0.013 for PM_2.5_; OR: 1.357, 95% CI: 1.165–1.581, *p* = 8.74 × 10^−5^ for NO_2_). Sensitivity analyses confirmed the robustness of these findings, with no significant heterogeneity or horizontal pleiotropy detected.

**Conclusion:**

Our study ascertained the causal correlations of air pollutants with lung function and COPD, emphasizing the importance of reducing air pollution. Interleukin-17A mediates the reduction of FEV_1_ and FVC by PM_10_, revealing potential therapeutic targets.

## Introduction

With the advancement of industrialization, air pollutants have progressively emerged as a significant public health and societal concern, resulting in negative health consequences (1). According to the current global air quality guidelines issued by the World Health Organization, nearly seven million people die each year from air pollution. The causes of death include noncommunicable diseases of the respiratory, cardiovascular, and other organs, resulting in a considerable socioeconomic burden (2). Therefore, air pollutants are regarded as one of the greatest environmental risks to human health. Various types of air pollutants have been identified, with the World Health Organization including nitrogen dioxide (NO_2_), particulate matter (PM), sulfur dioxide, and ozone in their classification in 2005 (3). PM consists of microscopic particles of varying sizes. The aerodynamic particle diameter determines PM deposition in the human body. Coarse particles, with a diameter of less than 10 μm, are primarily deposited in the larger conducting airways. Conversely, particles with a diameter of less than 2.5 μm can penetrate the alveolar-capillary barrier, entering the bloodstream, and adversely affecting the functioning of various organs in the human body (4). Despite the implementation of certain measures to control air pollution, its detrimental impact on human health caused by air pollution cannot be overlooked, particularly in developing countries. Many experimental and observational studies have consistently demonstrated the significant impact of air pollution on the development of various diseases, particularly chronic respiratory diseases (CRDs), and on unfavorable outcomes (5–7).

CRDs, as a type of non-communicable disease that recurs and deteriorates over time, are predominantly characterized by aberrant lung function and chronic airway inflammation, which ultimately lead to the death of patients due to respiratory failure accompanied by multi-organ complications. According to epidemiological surveys, CRDs have become one of the most common causes of mortality worldwide, ranking just below cardiovascular diseases and malignancies. Current data show that nearly 4 million people globally died from prevalent CRDs such as chronic obstructive pulmonary disease, asthma, and interstitial lung disease in 2017, posing a significant social and economic burden (8). Hence, it is imperative to conduct further research to ascertain the predisposing triggers of CRDs. While the exact cause of CRDs is complex and their pathogenesis is not completely understood, the shared characteristics of these conditions include airway blockage and limited airflow. Numerous observational and experimental studies have consistently identified a strong correlation between air pollutants and both reduced lung function and the acute worsening of CRDs (5, 9). For example, a clinic research conducted in Canada showed that per increase of 2.4 ug/m^3^ PM_2.5_ and 9.2 ppb NO_2_ induce 101.7 mL (95% confidence interval, −166.2 to −37.2) and 115.0 mL (95% confidence interval, −196.5 to −33.4) lower forced expiratory volume in the first second (FEV_1_), respectively (10). The underlying molecular mechanism involves an imbalance in reoxidation due to mitochondrial dysfunction, decreased levels of circulating fatty acid metabolites, and increased levels of inflammatory proteins, such as interleukin (11–13). Previous research shows that exposure to particulate pollutants can result in elevated levels of inflammatory proteins such as interleukin 6, C-reactive protein, and tumor necrosis factor, leading to a decrease of FEV_1_ and peak expiratory flow (14, 15). And air pollution induces activation of T-helper lymphocyte type 2 (Th2) and T-helper lymphocyte type 17 (Th17), contributing to dysregulation of the adaptive immune response (16). These findings indicate that air pollutants can cause airway obstruction and airflow limitation by inducing inflammation. However, given that observational research could be altered by undetected confounders, the reliability of the results is insufficient. Further research is required to evaluate the causal interaction of air pollutants with chronic lung diseases and lung function. In addition, exploring the mechanisms by which air pollution, as a risk factor, interferes with chronic lung disease could help develop new therapeutic targets.

Mendelian randomization (MR) is an epidemiological statistical method that applies genetic variation as an instrumental variable (IV), which is widely utilized to explore causality between exposure factors and outcomes. Since genetic variation is randomly assigned at the formation of a zygote, MR has the advantage of reducing confounding factors and avoiding reverse causation (17). In addition, compared to randomized controlled trials with a higher level of evidence, MR could generate reliable conclusions while consuming less time and at a lower cost.

Although several studies have shown a correlation between air pollutants and chronic lung diseases, evidence of causality is lacking. For example, Mohammadi et al. identified that polycyclic aromatic hydrocarbons, one of the components of PM, could induce chronic obstructive pulmonary disease (COPD) (18). Additionally, Seihei et al. also found that the exposure to PM10 could cause adverse health outcome (19). Therefore, MR approach was employed in the present study, aiming to provide deeper evidence for the causal role of air pollution in lung function and chronic lung diseases. Meanwhile, based on a two-step MR approach, the mediating role of inflammatory proteins was explored with the aim of providing potential therapeutic targets.

## Methods

In this study, the MR approach was utilized to investigate the causality of air pollution with CRDs and pulmonary function. Based on genome-wide association study (GWAS) data, MR adopts single nucleotide polymorphism (SNP) as an IV, effectively avoiding statistical bias in observational studies.

### Study design

**Figure 1:**
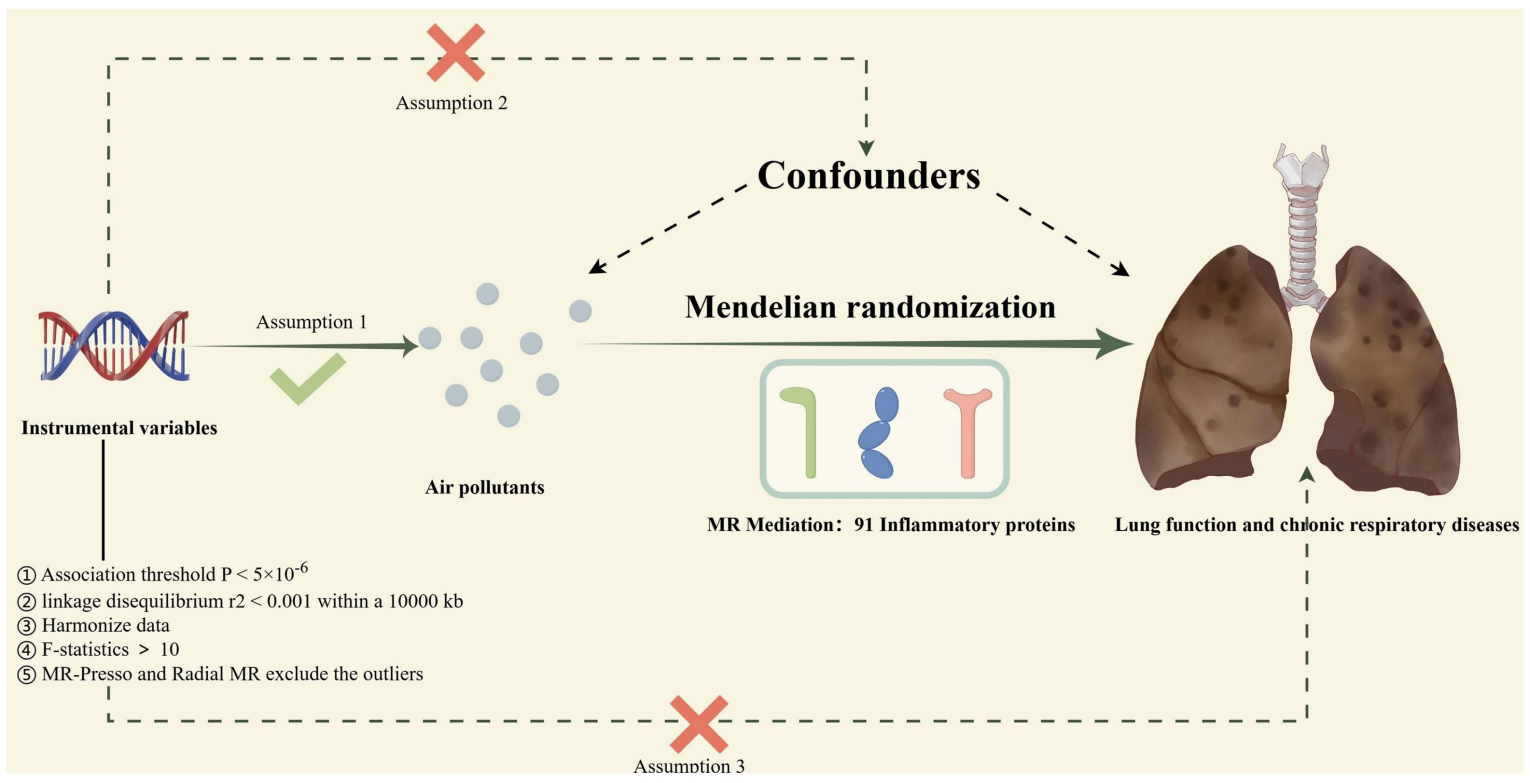
The process of the current Mendelian randomization (MR) study; Assumption 1: IVs must exhibit a significant association with exposure factors but not with outcomes; Assumption 2: IVs are independent of all potential confounders of exposure and outcome; Assumption 3: IVs can only influence outcomes through exposure factors; Mendelian Randomization Pleiotropy Residual Sum and Outlier; MR, Mendelian randomization.

The procedure for the research design is shown in Figure 1. Specifically, our study consisted of four steps. First, genetic variants were extracted as IVs from publicly available GWAS datasets based on the rigorous instrumental variable selection criteria. Second, we conducted a total of 18 two-sample MR to investigate the causal relationship between air pollutants and chronic lung diseases as well as lung function. Subsequently, comprehensive heterogeneity and pleiotropy tests were utilized to ensure the robustness of the findings. Finally, to explore potential mediators of inflammatory proteins, we calculated their mediating proportions using two-step MR. Crucially, our MR study was guided by three key assumptions to guarantee the validity and authenticity of the results. First, IV must exhibit a significant association with exposure factors, but not outcomes. Second, considering the potential confounding factors of exposure and outcome, the IVs remained independent. Third, the IV may affect outcomes only through exposure variables (20). All the data in the present study were obtained from a publicly available GWAS; therefore, no ethical approval was required.

### Data sources

As exposure factors, PM_2.5_ (dataset ID: ukb-b-10817), PM_10_ (dataset ID: ukb-b-589), and NO_2_ (dataset ID: ukb-b-5620) derived from the IEU Open GWAS^1^ database were involved in the present MR study. A land-use regression model was used to evaluate the air pollutant levels in the areas where the study participants were located. And the GWAS summary datasets of air pollutants were published in 2018. Subsequently, for the outcomes, we selected GWAS summary datasets of three CRDs from the Global Biobank Meta-Analysis Initiative, comprising asthma, COPD, and idiopathic pulmonary fibrosis (21). The database was established in 2019. In concrete terms, the COPD dataset is composed of 58,559 cases and 937,358 controls. The asthma dataset included 121,940 cases and 1,254,131 controls. And the idiopathic pulmonary fibrosis dataset contains 6,257 cases and 947,616 controls. In addition, we also selected the GWAS including FEV_1_, FVC, and FEV_1_/FVC ratio performed by Shrine et al., which is the largest pulmonary function dataset (*n* = 400,102) currently accessible (22).

Finally, to assess the mediating effects of inflammatory proteins on the causality of air pollution with CRDs and pulmonary function, we acquired summary datasets of 91 inflammatory proteins in the GWAS catalog (ID: GCST90274758-GCST90274848). Data for each inflammatory protein has been previously described in the research conducted by Zhao et al. (23).

### Selection of genetic instruments

In this study, the IVs of air pollution and inflammatory proteins were defined for MR analysis according to the following procedure. Initially, SNPs with a correlation threshold of *p* < 5 × 10^−6^ were screened as potential IVs. Meanwhile, the linkage disequilibrium *r*^2^ threshold was established at < 0.001, with a clumping window of 10,000 kb or greater to ensure the independence of IVs, which is a common practice in previous MR studies (24). Next, we extracted eligible SNPs from the lung function and CRDs summary statistical data, excluding those having significant associations with outcome datasets. After harmonization, SNPs that met the following criteria were excluded: (1) allele orientations cannot be aligned; (2) genetic variation with minor allele frequencies not exceeding 0.01; and (3) genetic instruments with palindromic sequences. Further, the formula *F* = (beta^2/se^2) was utilized to calculate the *F*-statistic of every single SNP, which aimed to ensure that the study results were not influenced by weak IVs. When the *F*-statistic is inferior to 10, the SNPs were excluded (25). Finally, to eliminate potential heterogeneity and horizontal pleiotropy caused by outliers, we applied MR-Pleiotropy Residual Sum and Outlier (MR-PRESSO) and radial MR, which could detect and eliminate outliers with a *p*-value less than 0.05 (26, 27). Therefore, SNPs that met the above screening criteria were retained as real IVs.

### Univariate Mendelian randomization

To determine the causal interaction of air pollution with lung function as well as CRDs, we utilized four statistical approaches in Univariate MR analyses. Among them, we executed the random-effect inverse variance weighted approach as the predominant criteria, which adopts a meta-analysis approach to aggregate Wald estimates for each SNP under the assumption that none of the genetic variants is an invalid IV (28). Moreover, as complementary techniques, three rigorous techniques derived from different statistical assumptions were integrated into the current MR. In concrete terms, MR-Egger, relying on the InSIDE assumption, can assess causality when all IVs are not valid (29). The weighted median approach, on the other hand, can provide an estimate even when half of the SNPs violate the IV assumption (30). Additionally, the constrained maximum likelihood and model averaging can eliminate bias induced by correlated and uncorrelated pleiotropy in the presence of invalid IVs (31).

### Heterogeneity and pleiotropy analysis

Extensive and rigorous heterogeneity and pleiotropy tests were conducted to ensure the robustness of the results. Specifically, Cochran’s *Q* method was used to evaluate the existence of heterogeneity. When the *p*-value of *Q* was >0.05, heterogeneity was not considered (32). The symmetry of the funnel plot supported the conclusions of the heterogeneity tests. Second, because horizontal pleiotropy violates the exclusion and independence hypotheses, the MR-PRESSO global test and MR-Egger intercept were used to validate the hypothesis (26, 29). Scatter plots were used to examine the directional consistencies of the four statistical methods. Finally, to detect the SNPs with a significant effect on the pooled inverse-variance weighted estimations, a leave-one-out analysis was conducted.

### Median MR

Based on the mediation MR approach, an area of analysis that attempts to detect causal pathways through which exposure affects outcomes and the mediating effects of circulating inflammatory proteins were evaluated in subsequent analyses (33). This study adopted a mediated MR analysis to calculate the causality of exposure with potential mediators and mediators with outcomes. SNPs that fulfilled the above screening criteria were screened from the circulating inflammatory protein dataset to infer causal interactions between air pollutants and circulating inflammatory proteins. Circulating inflammatory proteins that were strongly linked to air pollutants were selected to confirm how mediators and lung function, as well as CRDs, were linked. Using delta and Sobel tests, the intermediary effect, standard error (SE), and 95% confidence interval (CI) were calculated.

### Statistical analysis

Our research was conducted by utilizing the TwoSampleMR (version 0.5.10), MRPRESSO (version 1.0), Rmediation (version 1.2.2), and MendelianRandomization (version 0.8.0) packages in the R software (version 4.3.0) available at https://www.R-project.org. Our research used odds ratios (OR) and 95% confidence intervals to present the MR results. Confidence intervals are calculated from the standard error and the corresponding *z*-value, specifically effect size ± z * SE, where the *z*-value is usually taken to be 1.96 corresponding to a 95% confidence interval. *p*-values are calculated to test the statistical significance of the null hypothesis by comparing the observed statistic with the reference distribution of its distribution. Additionally, considering that multiple analyses contribute to the extended probability of type I error, the Bonferroni correction was applied to correct the p-threshold for inverse-variance weighted to 2.78 × 10^−3^ (0.05/18). Correlations with *p*-values between 0.05 and 0.00278 were considered to be nominal evidence of association, which indicated that there may be a potential association that warrants further exploration.

## Results

Using comprehensive SNP screening criteria, IVs for the MR analysis that complied with the three hypotheses were identified. Specifically, for the MR analyses of air pollutants on lung function and CRDs, this study incorporated genetic instruments ranging from 38 to 210, and each *F*-statistic of the IVs was not lower than 20.86, eliminating statistical bias due to weak IVs. All the outliers identified and discarded using the radial MR approach are listed in Supplementary Table S1. Similarly, regarding mediator MR analysis, it was confirmed that there was a minimum of three IVs for each inflammatory protein, and no weak IV was detected.

### Univariate Mendelian randomization

**Figure 2:**
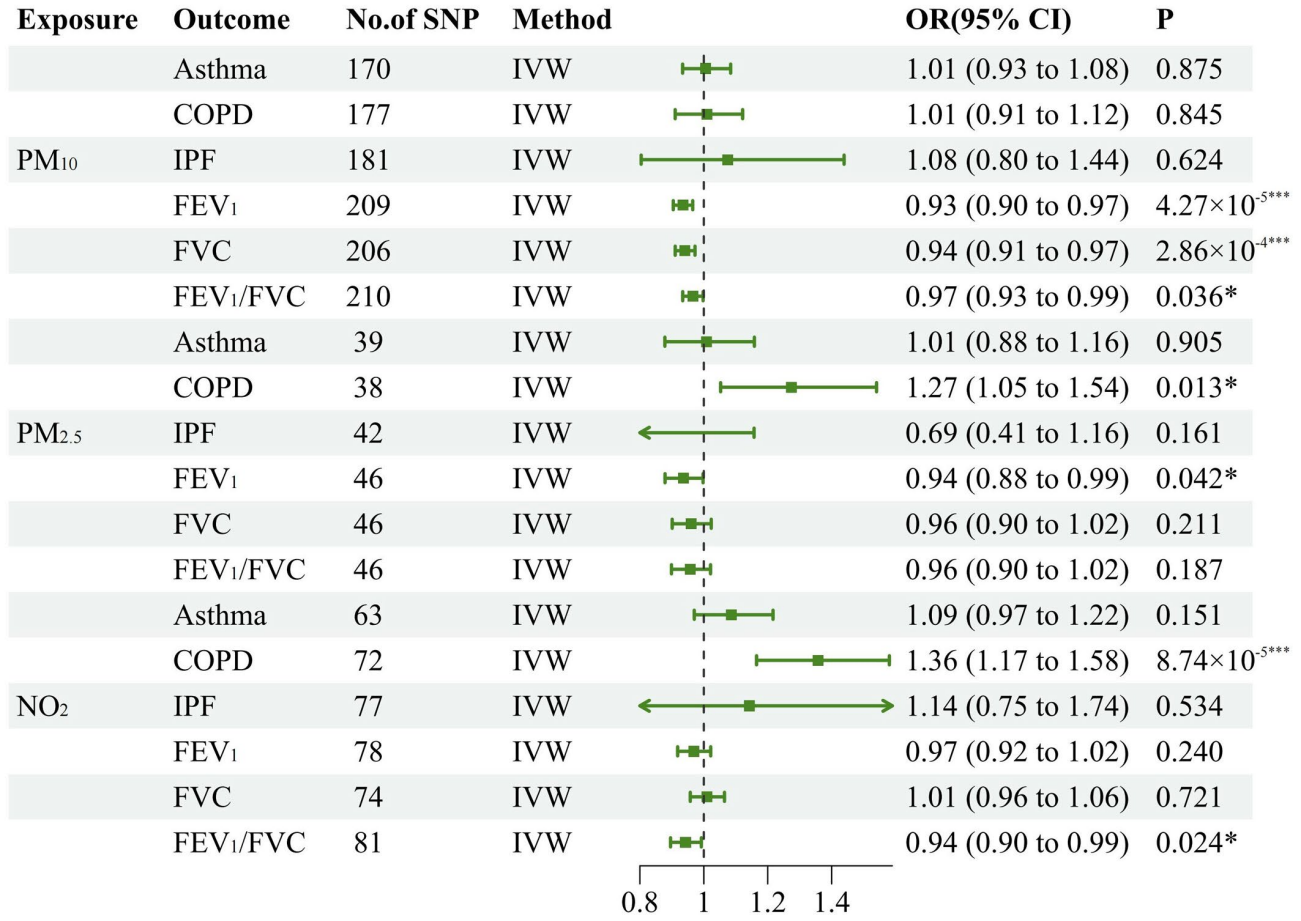
Forest plot for the causal effect of air pollution on the risk of chronic pulmonary diseases and lung function. The green line represents the 95% confidence interval for a study's effect size and the dots colored in green in the middle of the line represents the estimate of the effect size. PM, particulate matter; N02, nitrogen dioxide; COPD, chronic obstructive pulmonary disease; IPF, idiopathic pulmonary fibrosis; FEVI, forced expiratory volume in one second; FVC, forced vital capacity; IVW, inverse variance weighted; *p<0.05; **p<0.01;***p<0.001.

To estimate the causal effects of various air pollutants on lung function and CRDs, we conducted a total of 18 MR analyses. Initially, our study substantiated the causal interaction of PM_2.5_, PM_10_, and NO_2_ with the decline of lung function indicators. In concrete terms, the inverse variance weighted approach established negative causality of PM_10_ on FEV_1_ (OR: 0.934, 95% CI: 0.904–0.965, *p* = 4.27 × 10^−5^), FVC (OR: 0.941, 95% CI: 0.910–0.972, *p* = 2.86 × 10^−4^), and FEV_1_/FVC (OR: 0.965, 95% CI: 0.934–0.998, *p* = 0.036). Additionally, our research has revealed that PM_2.5_ was a potential risk factor for impairing FEV_1_ (OR: 0.936, 95% CI: 0.879–0.998, *p* = 0.042). Similarly, genetically predicted NO_2_ may also be a potential risk factor for diminishing FEV_1_/FVC (OR: 0.943, 95% CI: 0.896–0.992, *p* = 0.024). Meanwhile, for CRDs, PM_2.5_ has a potential causal interaction with the incidence of COPD (OR: 1.273, 95% CI: 1.053–1.541, *p* = 0.013). In addition, NO_2_ also contributes to the development of COPD (OR: 1.357, 95% CI: 1.165–1.581, *p* = 8.74 × 10^−5^). Since the significance threshold was corrected to 0.0278 after applying Bonferroni’s approach to avoid type one error, we considered that there was suggestive causality at 0.00278 < *p* < 0.05. All the MR results embracing the inverse variance weighted analytical models have been displayed in Figure 2, and the other three models were tabulated in Table 1.

**Table 1:** Mendelian randomization results from complementary analytical models.

Air pollution	Outcome	MR egger		Weighted median		Constrained maximum likelihood	
		OR (95%CI)	*p*-value	OR (95%CI)	*P*-value	OR (95%CI)	*P*-value
PM_2.5_	Lung function						
	FEV_1_	0.897 (0.769–1.045)	0.171	0.935 (0.852–1.027)	0.163	0.935 (0.871–1.003)	0.061
	FVC	0.963 (0.823–1.126)	0.638	0.955 (0.872–1.047)	0.330	0.963 (0.896–1.034)	0.296
	FEV_1_/FVC	0.810 (0.692–0.948)	0.012^*^	0.967 (0.912–1.092)	0.894	0.957 (0.893–1.026)	0.218
	CRDs						
	Asthma	1.061 (0.810–1.390)	0.670	1.120 (0.918–1.366)	0.263	1.006 (0.873–1.159)	0.934
	COPD	1.085 (0.753–1.564)	0.664	1.158 (0.853–1.573)	0.347	1.270 (1.033–1.561)	0.023^*^
	IPF	0.975 (0.346–2.741)	0.961	0.689 (0.308–1.541)	0.364	0.711 (0.393–1.289)	0.261
PM_10_	Lung function						
	FEV_1_	0.964 (0.897–1.037)	0.331	0.946 (0.901–0.994)	0.027^*^	0.932 (0.897–0.967)	2.22 × 10^–4***^
	FVC	0.981 (0.911–1.056)	0.602	0.955 (0.910–1.002)	0.059	0.941 (0.907–0.976)	0.001^**^
	FEV_1_/FVC	0.961 (0.893–1.034)	0.287	0.960 (0.917–1.005)	0.081	0.964 (0.928–1.001)	0.058
	CRDs						
	Asthma	1.011 (0.858–1.192)	0.894	1.014 (0.909–1.131)	0.801	1.006 (0.927–1.091)	0.886
	COPD	0.904 (0.716–1.143)	0.400	1.002 (0.861–1.167)	0.978	1.013 (0.910–1.127)	0.819
	IPF	1.292 (0.659–2.533)	0.456	1.160 (0.766–1.758)	0.484	1.078 (0.773–1.504)	0.657
NO_2_	Lung function						
	FEV_1_	0.973 (0.867–1.092)	0.642	0.962 (0.891–1.039)	0.329	0.969 (0.921–1.020)	0.229
	FVC	1.006 (0.896–1.131)	0.914	1.013 (0.941–1.090)	0.740	1.008 (0.953–1.067)	0.771
	FEV_1_/FVC	0.946 (0.845–1.060)	0.344	0.947 (0.879–1.019)	0.146	0.943 (0.892–0.996)	0.035^*^
	CRDs						
	Asthma	1.013 (0.810–1.267)	0.911	1.073 (0.912–1.263)	0.394	1.092 (0.965–1.192)	1.238
	COPD	1.412 (1.022–1.950)	0.040^*^	1.261 (1.001–1.590)	0.049^*^	1.366 (1.143–1.634)	6.29 × 10^–4***^
	IPF	1.198 (0.478–3.002)	0.701	1.307 (0.682–2.505)	0.419	1.150 (0.739–1.790)	0.536

CI, confidence intervals are calculated using effect size ± z * SE, where the *z*-value is usually taken to be 1.96 corresponding to a 95% confidence interval; OR, odds ratio; PM, particulate matter; NO2, nitrogen oxides; FEV1, forced expiratory volume in one second; FVC, forced vital capacity; CRDs, chronic respiratory disease; COPD, chronic obstructive pulmonary disease; IPF, idiopathic pulmonary fibrosis; ^*^p < 0.05; ^**^p < 0.01; ^***^p < 0.001.

### Heterogeneity and pleiotropy analysis

Comprehensive sensitivity analyses were implemented to determine whether the causal interactions obtained from the above analyses were inaccurate because of violations of the IV assumptions. Initially, the directional consistency of the scatter plot shown in Supplementary Figure S1 confirmed the credibility of the MR analysis results. Subsequently, we preliminarily excluded the existence of heterogeneity on the basis of the Cochran *Q* test with *p*-values all exceeding 0.05. The symmetry of the funnel plot shown in Supplementary Figure S2 confirmed the conclusions of the above test. Additionally, we utilized the MR-PRESSO global test and the MR-Egger intercept test to estimate the horizontal pleiotropy. The detailed results of Cochran *Q* and horizontal pleiotropy evaluations were exhibited in Table 2. Ultimately, the leave-one-out (LOO) analysis shown in Supplementary Figure S3 was executed to ascertain which SNPs significantly impact the pooled inverse variance weighted estimations. Overall, heterogeneity and horizontal pleiotropy were not observed in the current study, reinforcing the credibility of the MR results.

**Table 2:** Results of Cochran *Q* test and horizontal pleiotropy.

Air pollution	Outcome	Cochran’s *Q* test(IVW)		Egger		MR-PRESSO global test
		*Q*	*p*-value	Intercept	*p*-value	*p*-value
PM_2.5_	Lung function					
	FEV_1_	44.300	0.501	7.02 × 10^−4^	0.549	0.504
	FVC	38.242	0.752	−4.42 × 10^−5^	0.971	0.762
	FEV_1_/FVC	32.409	0.920	2.86 × 10^−3^	0.027^*^	0.917
	CRDs					
	Asthma	42.189	0.295	−9.85 × 10^−4^	0.669	0.272
	COPD	28.281	0.848	3.13 × 10^−3^	0.300	0.322
	IPF	129.790	0.998	3.20 × 10^−3^	0.554	0.997
	Lung function					
	FEV_1_	174.564	0.956	−5.67 × 10^−4^	0.342	0.960
	FVC	206.895	0.450	−7.32 × 10^−4^	0.223	0.464
PM_10_	FEV_1_/FVC	178.030	0.941	8.43 × 10^−5^	0.886	0.945
	CRDs					
	Asthma	138.664	0.958	−9.25 × 10^−5^	0.945	0.960
	COPD	160.545	0.792	1.99 × 10^−3^	0.300	0.778
	IPF	129.790	0.998	3.20 × 10^−3^	0.554	0.997
	Lung function					
	FEV_1_	83.468	0.288	−8.03 × 10^−5^	0.931	0.297
	FVC	62.246	0.811	5.68 × 10^−5^	0.951	0.801
NO_2_	FEV_1_/FVC	59.141	0.961	−6.67 × 10^−5^	0.942	0.958
	CRDs					
	Asthma	50.059	0.862	1.31 × 10^−3^	0.480	0.861
	COPD	58.953	0.846	6.97 × 10^−4^	0.787	0.860
	IPF	61.893	0.879	−8.04 × 10^−4^	0.911	0.884

PM, particulate matter; NO2, nitrogen oxides; FEV1, forced expiratory volume in one second; FVC, forced vital capacity; CRDs, chronic respiratory disease; COPD, chronic obstructive pulmonary disease; IPF, idiopathic pulmonary fibrosis; ^*^p < 0.05.

### Median MR

**Figure 3:**
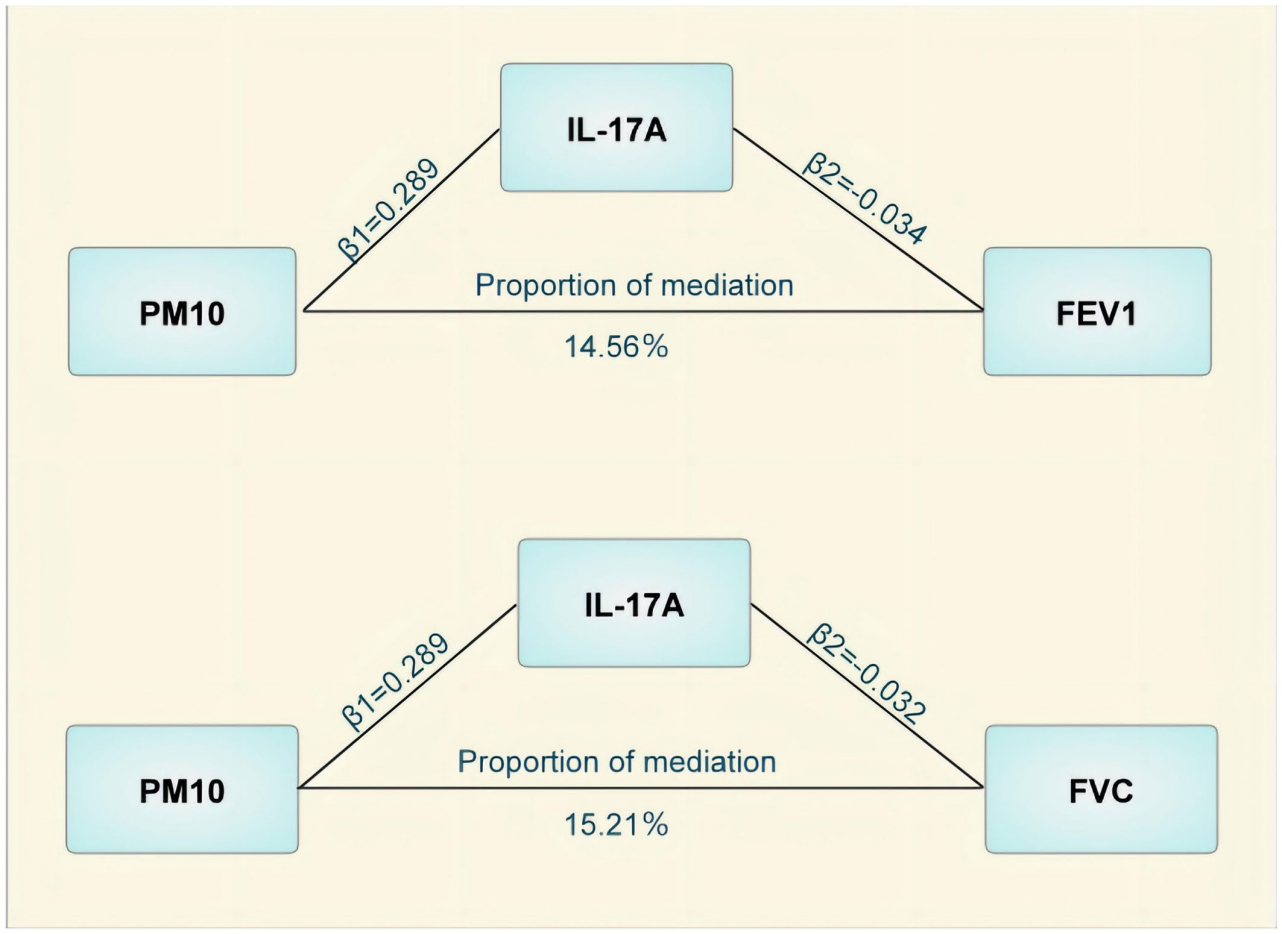
Mediating effects of Interleukin-17A in the causal effect of air pollution on lung function. PM, particulate matter; IL-17A, Interleukin-17A; FEVI, forced expiratory volume in one second; FVC, forced vital capacity.

The mediated MR analysis examined the causal correlations between the three pollutants and 91 inflammatory proteins. Ten inflammatory proteins that were causally related to pollutants, including seven with PM_10_, two with PM_2.5_, and one with NO_2_ (Table 3) were identified. These inflammatory proteins were used as exposure factors to further investigate their causal contribution to lung function and CRDs. Among them, Axin-1 was associated with decreased FEV_1_ and FEV_1_/FVC, IL-17A could cause decreased FEV_1_ and FVC, and IL-33 is a potential protective factor against FVC impairment. Additionally, the T-cell surface glycoprotein CD6 isoform was found to decrease FEV_1_/FVC. The results of the MR analysis for the causality of the 10 inflammatory proteins with COPD and pulmonary function are presented in Table 3. However, only IL-17A mediated the PM_10_-induced decrease in FEV_1_ and FVC after utilizing the delta and Sobel tests, with mediating effects accounting for 14.56 and 15.21%, respectively (Figure 3).

**Table 3:** Results of the mediation effects analysis.

Air pollutants	Inflammatory protein	Lung function and CRDs associated with exposures	OR (95%CI)	*p*-value
	OR (95%CI), *p*-value as outcome associated with air pollutants			
PM_2.5_	Interleukin-2 0.596 (0.436–0.816) *p* = 0.001^**^	FEV_1_	0.998 (0.978–1.019)	0.876
		COPD	1.001 (0.936–1.069)	0.996
	Neurotrophin-3 0.697 (0.529–0.920) *p* = 0.011^*^	FEV_1_	1.004 (0.984–1.025)	0.693
		COPD	1.030 (0.966–1.098)	0.374
PM_10_	Axin-1 0.840 (0.707–0.998) *p* = 0.048^*^	FEV_1_	0.968 (0.946–0.990)	0.005^**^
		FVC	0.987 (0.964–1.010)	0.267
		FEV_1_/FVC	0.961 (0.939–0.984)	0.001^**^
	Natural killer cell receptor 2B4 0.809 (0.694–0.944) *p* = 0.007^**^	FEV_1_	0.995 (0.978–1.012)	0.554
		FVC	0.996 (0.979–1.013)	0.644
		FEV_1_/FVC	0.990 (0.974–1.006)	0.234
	CD40L receptor 0.808 (0.694–0.940) *p* = 0.006^**^	FEV_1_	1.001 (0.990–1.011)	0.933
		FVC	0.996 (0.986–1.007)	0.513
		FEV_1_/FVC	1.003 (0.993–1.013)	0.541
	U-cell surface glycoprotein CD6 isoform 0.830 (0.710–0.969) *p* = 0.018^*^	FEV_1_	0.998 (0.990–1.006)	0.651
		FVC	1.001 (0.992–1.009)	0.936
		FEV_1_/FVC	0.991 (0.983–0.999)	0.041^*^
	Interleukin-17A 1.335 (1.123–1.587) *p* = 0.001^**^	FEV_1_	0.966 (0.946–0.987)	0.001^**^
		FVC	0.968 (0.9470.991)	0.005^**^
		FEV_1_/FVC	0.998 (0.977–1.018)	0.817
	Interleukin-33 0.801 (0.674–0.952 *P* = 0.01^*^)	FEV_1_	1.018 (0.995–1.042)	0.130
		FVC	1.028 (1.005–1.052)	0.018^*^
		FEV_1_/FVC	0.984 (0.957–1.011)	0.248
	STAM binding protein 0.827 (0.708–0.966) *p* = 0.016^*^	FEV_1_	0.999 (0.972–1.027)	0.953
		FVC	0.999 (0.973–1.028)	0.994
		FEV_1_/FVC	0.999 (0.971–1.027)	0.922
NO_2_	Matrix metalloproteinase-10 1.436 (1.134–1.819) *p* = 0.003^**^	COPD	1.005 (0.971–1.040)	0.774
		FEV_1_/FVC	0.998 (0.980–1.017)	0.826

PM, particulate matter; NO2, nitrogen oxides; FEV1, forced expiratory volume in one second; FVC, forced vital capacity; CRDs, chronic respiratory disease; COPD, chronic obstructive pulmonary disease; IPF, idiopathic pulmonary fibrosis; ^*^p < 0.05; ^**^p < 0.01.

## Discussion

Based on the utilization of publicly available summary GWAS datasets, the current study implemented a rigorous two-sample MR analysis to investigate the causal interaction of air pollutants (comprising NO_2_, PM_2.5_, and PM_10_) on lung function and CRDs, for which the mediating role of inflammatory proteins was substantiated by the two-step MR analysis. Finally, we confirmed the negative regulatory effects of several air pollutants on lung function and their contribution to the incidence of COPD. Notably, IL-17A plays an indispensable character in the process by which PM_10_ compromises lung function. To our knowledge, the current study is the first to utilize univariate and mediated MR to elucidate the above issues.

Air pollution is an important environmental trigger for morbidity and premature death, and has emerged as a global public health problem. According to the Global Burden of Disease analysis, 6.67 million premature mortalities in 2019 could be attributed to the involvement of air pollution (34). Extensive epidemiological and toxicological studies have demonstrated that excessive inhalation of air pollutants is strongly associated with the incidence of cardiovascular and respiratory diseases, cancer, and neurological disorders (35–37). Since the respiratory system is the most predominant part of the body exposed to inhaled air pollutants, it is the most targeted organ for the toxic effects of air pollutants. The morbidity of asthma, COPD, idiopathic pulmonary fibrosis, and other CRDs has been frequently estimated to be correlated with air pollution in the published literature (38, 39). Additionally, since CRDs are characterized by airway obstruction and airflow limitation, it is essential to observe lung function indicators to evaluate the progression of the diseases. Therefore, air pollutant-induced lung function impairment has also been extensively discussed in the previous literature (40). However, due to the vulnerability of observational studies to confounding factors and the lack of genetic evidence, further research to confirm these results is desirable. Consequently, we designed the comprehensive MR study aiming to provide further research evidence for the causal contribution of air pollution to lung function and CRDs.

In our study, NO_2_ was demonstrated to have a significant causal interaction with the incidence of COPD, whereas PM_2.5_ was the indicative risk factor, which is compatible with the results of previous studies. For instance, a clinical study conducted by Hoffmann et al. in the Berlin area revealed that exposure to NO_2_ induced an increased risk of hospitalization in patients with COPD (41). Moreover, a case-crossover study including 449 participants also revealed that inhalation of either PM_2.5_ or NO_2_ exacerbated respiratory symptoms such as coughing, sputum, and dyspnea in patients with COPD (42). Compared to these studies, our study utilized genetic variants as IVs, providing genetic evidence and more reliable conclusion for the causal relationship between air pollutants and COPD. For potential molecular mechanisms, PM_2.5_ is composed of various types of metal elements, inorganic salts, organic substances, and other deleterious substances that can enter the alveoli with respiratory activities. Therefore, on the one hand, it can directly stimulate the alveolar mucosa and pericapillary cells, inducing various types of hypersensitivity or toxicity. For instance, clinical studies have indicated a positive association between exposure to NO_2_ and serum IL-6 levels (43). As an essential pro-inflammatory cytokine, IL-6 is a potential predictive marker for the frequency of COPD exacerbations and significantly associated with progressive airflow obstruction (44). On the other hand, experimental studies have found that PM_2.5_ could induce redox imbalance and thus damage mitochondria, contributing to the inflammatory response in COPD patients. In addition, although the causal correlation of air pollutants with asthma and idiopathic pulmonary fibrosis was not established in this study, the impairment of lung function indicated their contributing role in causing airflow obstruction. Data collected by Lee et al. revealed the same conclusion that inhalation of PM_2.5_ and PM_10_ contributed to FEV_1_ impairment even for athletes with superior physical condition (45). Similarly, the cross-sectional investigation conducted by Havet et al. also verified that NO_2_ and PM_10_ negatively modulated FEV_1_, FVC, and FEV_1_/FVC (46). Our findings are consistent with the above studies that air pollutants are one of the important risk factors for inducing airway obstruction and airflow limitation and can aggravate respiratory diseases by impairing lung function.

Notably, in accordance with the intermediary MR analysis, IL-17A was identified as a mediator contributing to the downregulation of FEV_1_ and FVC by PM_10_ in our study. It is well known that lung function impairment and the pathogenesis of chronic lung diseases are intimately related to airway inflammation (47). After air pollutants are inhaled into the respiratory system, respiratory epithelial cells and macrophages are stimulated to release cytokines and chemokines, and immune cells are further activated by them to secrete more cytokines, which include pro-inflammatory factors such as IL-1β, IL-2, IL-6, IL-17A, and so on. For instance, PM_2.5_ was detected to dramatically increase IL-1β and tumor necrosis factor alpha activities in mouse lung lavage, implying that air pollutants can induce and exacerbate lung inflammation (48). Among them, IL-17A is a cytokine secreted by T helper cell 17 (Th17) with pro-inflammatory and pro-fibrotic functions. It has been revealed that peripheral blood Th17 and IL-17 levels were significantly elevated in patients with COPD and negatively correlated with FEV_1_, indicating that peripheral blood Th17 and L-17A correlate well with lung function in patients with COPD, and that they are effective indicators for early prediction of COPD (49). The above studies indicated that IL-17A is a potential mediator of air pollutant impairment of lung function, which is consistent with our findings. In brief, our study offered valuable insights and therapeutic targets for the treatment of air pollutant-induced lung function decline, and it is meaningful to further investigate the role of IL-17A inhibitors in CRDs.

Combining our results with previous studies, there are several research directions worth implementing in the future. First, our study provides genetic evidence that air pollution impairs lung function and induces chronic respiratory diseases, and further exploration of the interactions between air pollution and genetic factors can help reveal the genetic susceptibility to air pollution pathogenesis. Second, the mediating effect of interleukin-17A suggests an important role of inflammatory mechanisms in air pollution pathogenesis, and targeting inflammatory mediators to ameliorate air pollution-induced lung function impairment deserves further investigation. Third, although we relied on statistically rigorous methods to illustrate the causal relationship between air pollution and chronic lung diseases and lung function, this still needs to be further validated by multiple databases. Cohort studies with long-term follow-up could be useful to validate our conclusions.

With a rigorous univariate and mediated MR approach, our research has the following advantages: (1) We minimized statistical bias introduced by potential confounders in observational and experimental studies by utilizing genetically predicted phenotypes as exposures; (2) multiple statistical models and comprehensive sensitivity analyses were implemented to ensure the robustness of causality; and (3) mediating MR analyses provided indicative evidence for potential molecular mechanisms.

Additionally, our study also have several limitations. First, our findings may not be applicable to other populations since GWAS data were all derived from European ancestry. Second, the screening threshold for IVs was extended to 5 × 10^−6^, which may introduce false positives. Fortunately, the implementation of the *F*-value formula addressed the bias introduced by weak instrumental variables. Finally, exploration of causality with summary data limited the ability to further stratify analyses. However, despite these limitations, our mediated MR analysis provides valuable insights into the causal role of air pollutants in COPD and lung function.

## Conclusion

In brief, our two-sample MR study provides relatively stronger evidence that air pollution impairs lung function and contributes to the development of COPD. It emphasizes once again the necessity of controlling air pollution. Reducing pollutants emissions and switching to cleaner energy sources are feasible initiatives. Meanwhile, the two-step MR analysis also provides evidence that targeting interleukin-17A to ameliorate air pollution-induced lung function impairment. Further studies to investigate the molecular mechanisms may contribute to the prevention and treatment of CRDs.

## Data Availability

Publicly available datasets were analyzed in this study. This data can be found at: https://gwas.mrcieu.ac.uk/; https://www.ebi.ac.uk/gwas/downloads/summary-statistics; https://www.globalbiobankmeta.org/.

## Footnotes

^1^https://gwas.mrcieu.ac.uk/datasets
