# Supplementary material for: Causal effects of air pollutants on lung function and chronic respiratory diseases: a Mendelian randomization study
Source: Front Public Health. 2024 Sep 9;12:1438974. doi: 10.3389/fpubh.2024.1438974 (PMC11416934; doi:10.3389/fpubh.2024.1438974)
Supplement: Supplementary file 1 [file Data_Sheet_1.DOCX]

## Supplemental Materials

To investigate the causality between air pollution and lung function, as well as chronic respiratory diseases, we conducted a total of 18 MR analyses in the R software. Subsequently, we utilized the datasets of 91 inflammatory proteins to assess their mediating effects. The R codes utilized in our study are as follows:

#run mr analysis

mr_fun <- function(id_exposures, id_outcomes, p1=5e-6,

method_list = c("mr_ivw_mre", "mr_egger_regression", "mr_weighted_median"),

rm_snps=NULL){

#1.read ieu exposure data

exposure_data <- TwoSampleMR::extract_instruments(id_exposures, p1)

#remove some snps

if(is.null(rm_snps)){

exposure_data <- subset(exposure_data, !SNP %in% rm_snps)

}

#2.read local outcome data

outcome_data <- TwoSampleMR::extract_outcome_data(exposure_data$SNP, id_outcomes, proxies = F)

#3.harmonise data

harmonise_data <- TwoSampleMR::harmonise_data(exposure_data, outcome_data)

#4.run mr analysis

mr_res <- TwoSampleMR::mr(harmonise_data, method_list = method_list)

#5. run presso

presso_res<-MRPRESSO::mr_presso(harmonise_data$beta.outcome,harmonise_data$beta.exposure, harmonise_data$se.outcome, harmonise_data$se.exposure)

return(mr_res)

}

mediation_fun <- function(id_exposure, id_mediation, id_outcome){

#1.run exposure to mediator mr

dat_exposure_mediator <- mr_fun(id_exposure, id_mediation, method_list = "mr_ivw")

#2.run exposure to outcome mr

dat_exposure_outcome <- mr_fun(id_exposure, id_outcome, method_list = "mr_ivw")

#3.run mediator to outcome mr

dat_mediator_outcome <- mv_fun(id_mediation, id_outcome, method_list = "mr_ivw")

#4.calculate mediation effect

a <- as.numeric(dat_exposure_mediator$b)

b <- as.numeric(dat_mediator_outcome$b)

c1 <- as.numeric(dat_exposure_outcome$b)

sa <<- as.numeric(dat_exposure_mediator$se)

sb <<- as.numeric(dat_mediator_outcome$se)

#5.calculat effect

total_effect <- c1

mediator_effect <- a * b

direct_effect <- c1 - IE

mediator_proportion <- IE / TE

}
