# Supplementary material for: Causal effects of air pollutants on lung function and chronic respiratory diseases: a Mendelian randomization study
Source: Front Public Health. 2024 Sep 9;12:1438974. doi: 10.3389/fpubh.2024.1438974 (PMC11416934; doi:10.3389/fpubh.2024.1438974)
Supplement: Supplementary file 2 [file Image_1.PDF]

Supplementary Figure

Figure S1. Scatter plots of air pollution with CRDs and lung function

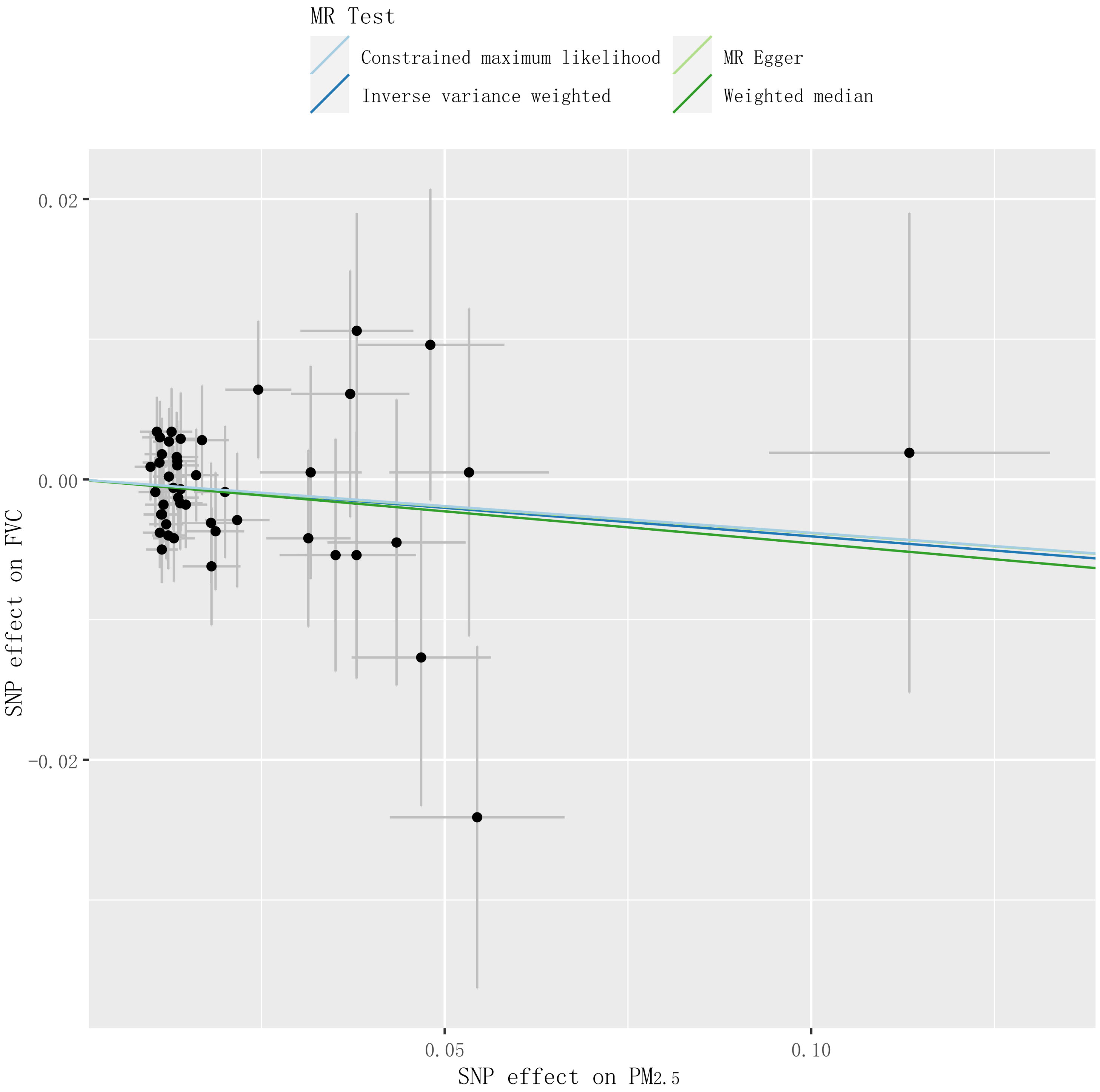

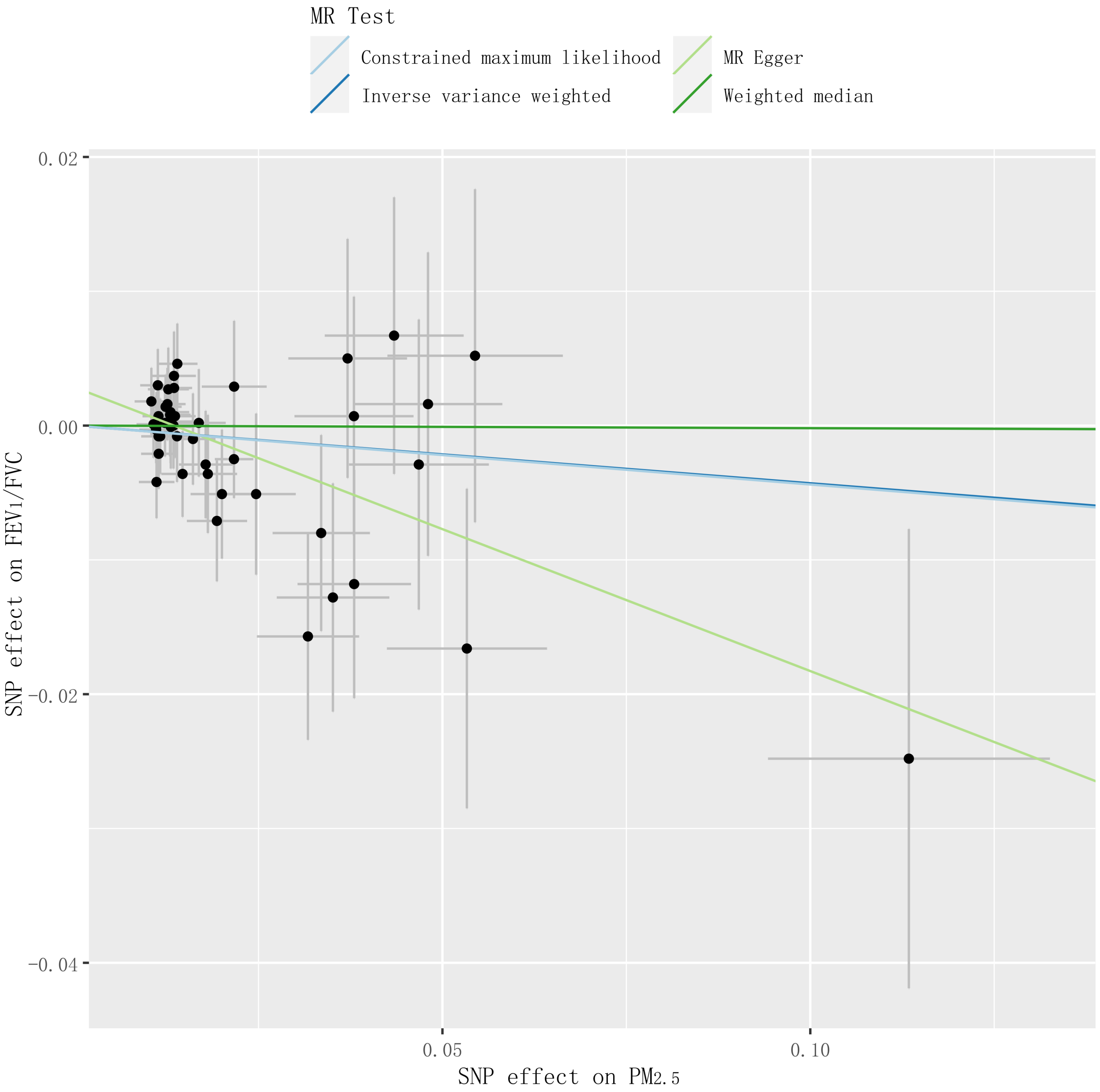
MR Test
Constrained maximum likelihood
Inverse variance weighted
MR Egger
Weighted median
0.02
0.00
−0.02
−0.04
0.05
0.10
SNP effect on FEV1/FVC
SNP effect on PM2.5

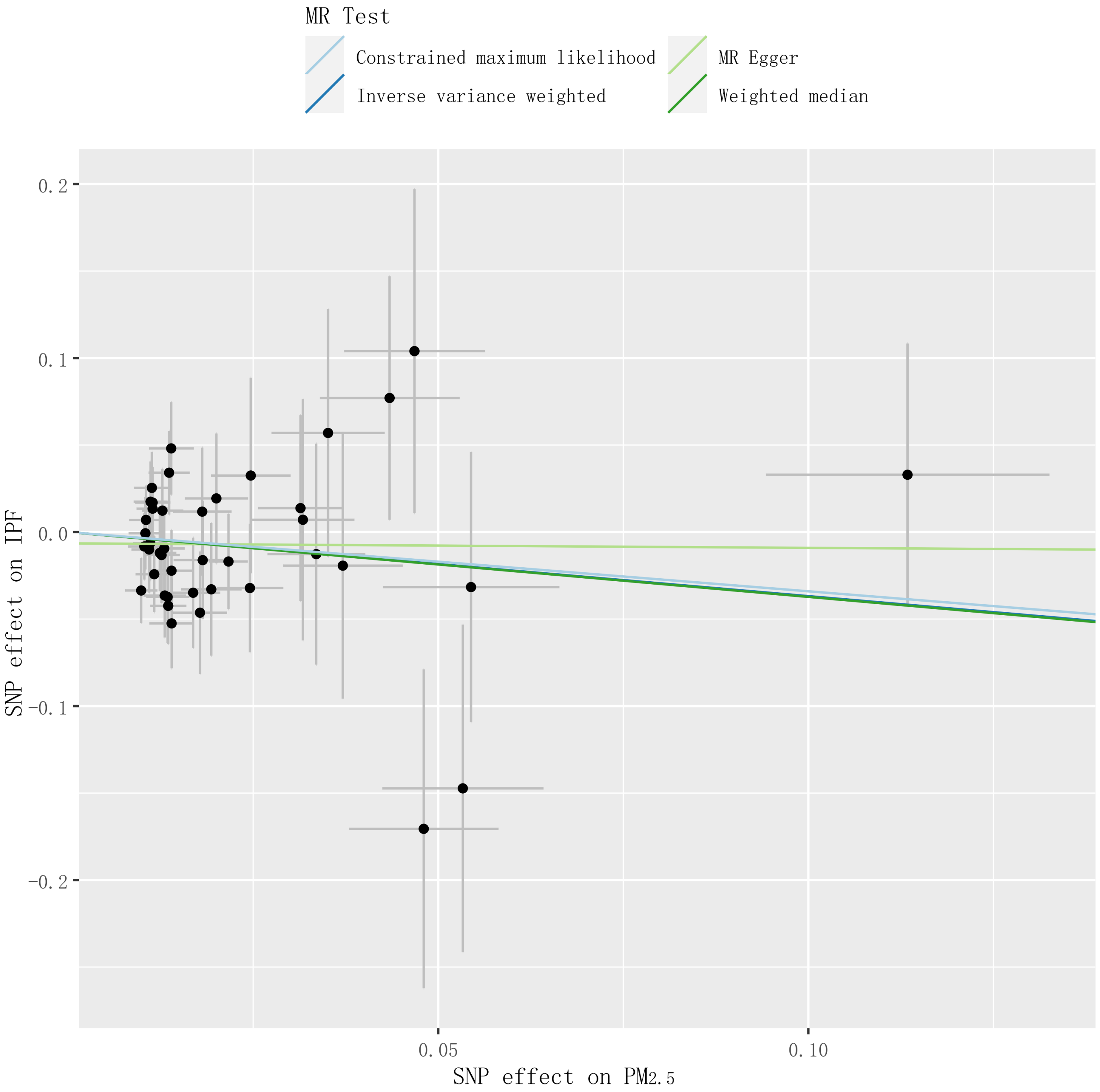
MR Test
Constrained maximum likelihood
Inverse variance weighted
MR Egger
Weighted median
0.2
0.1
0.0
−0.1
−0.2
SNP effect on IPF
0.05
0.10
SNP effect on PM2.5

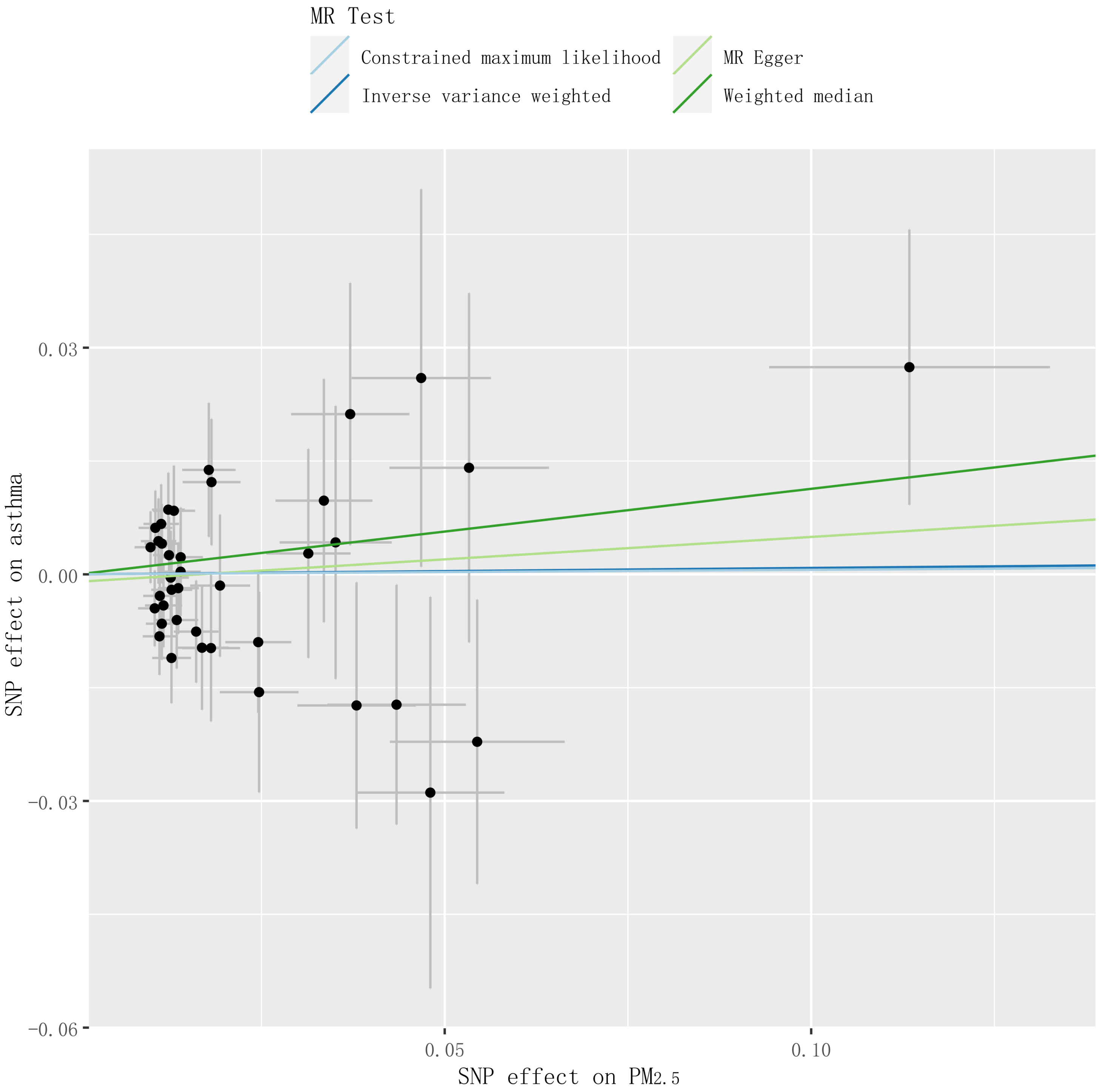

MR Test
Constrained maximum likelihood
Inverse variance weighted
MR Egger
Weighted median
0.03
0.00
−0.03
−0.06
0.05
0.10
SNP effect on asthma
SNP effect on PM2.5

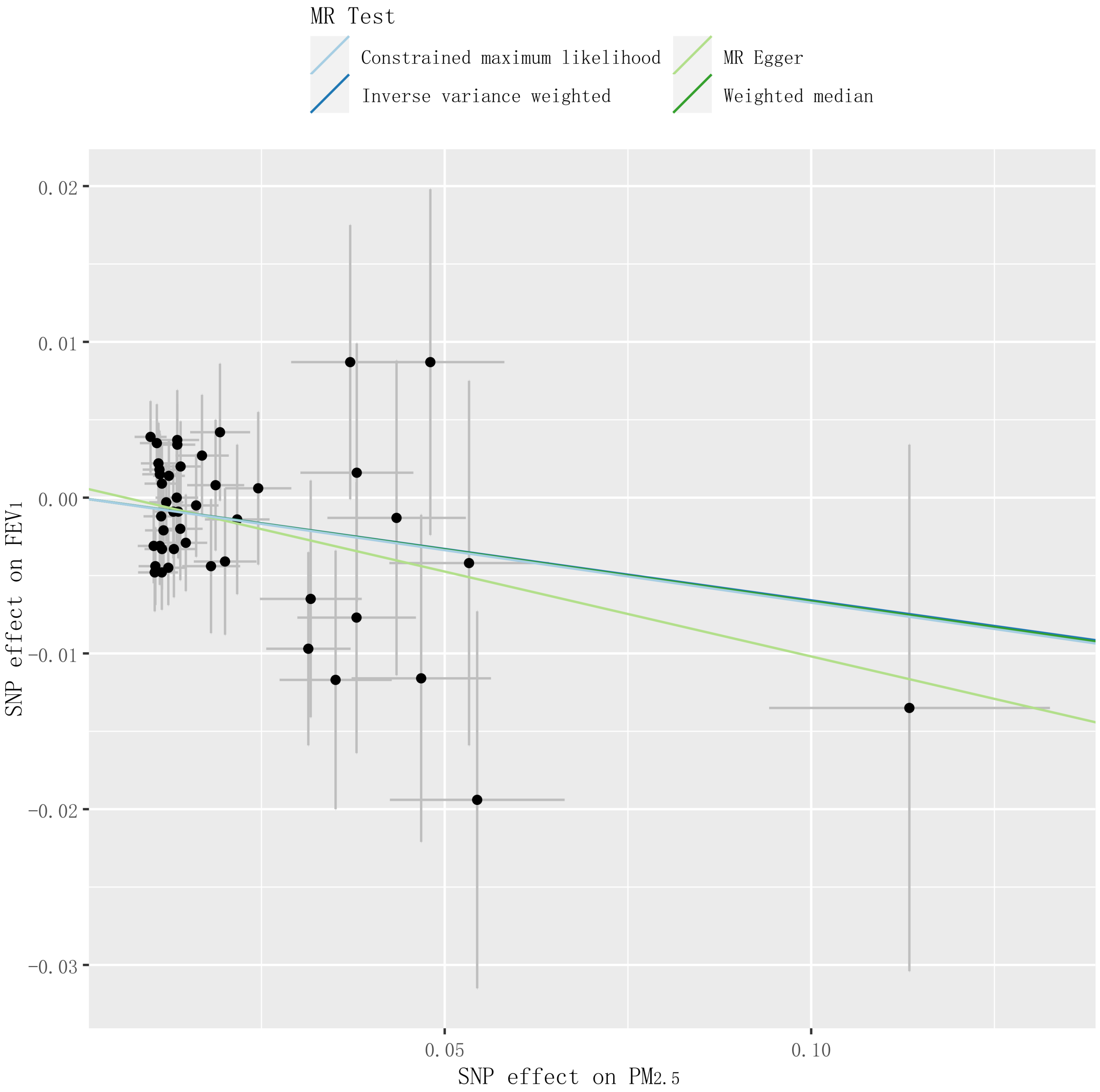
MR Test
Constrained maximum likelihood
Inverse variance weighted
MR Egger
Weighted median
0.02
0.01
0.00
−0.01
−0.02
−0.03
0.05
0.10
SNP effect on PM2.5
SNP effect on FEV1

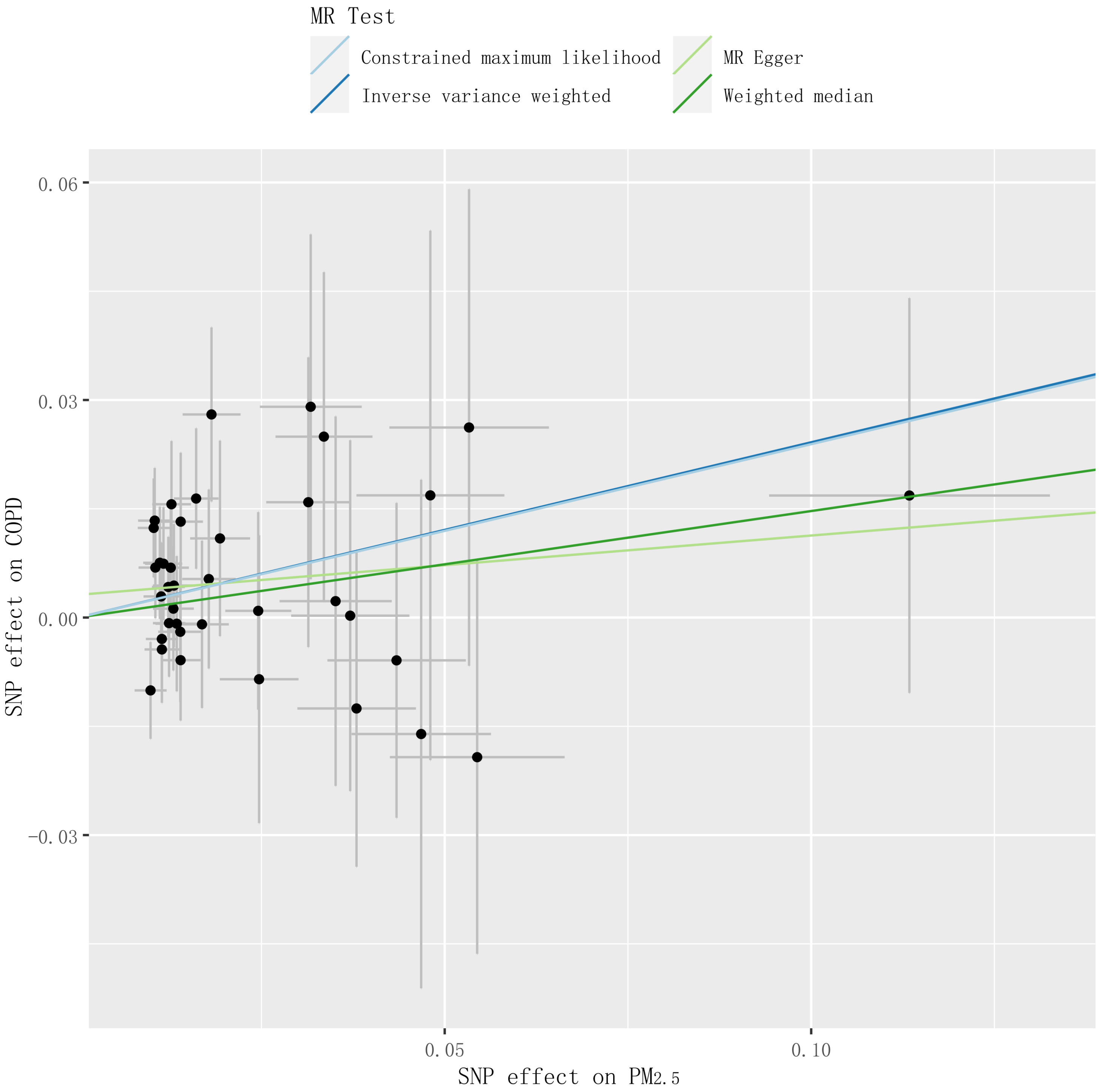
MR Test
Constrained maximum likelihood
Inverse variance weighted
MR Egger
Weighted median
0.06
0.03
0.00
-0.03
SNP effect on COPD
0.05
0.10
SNP effect on PM2.5

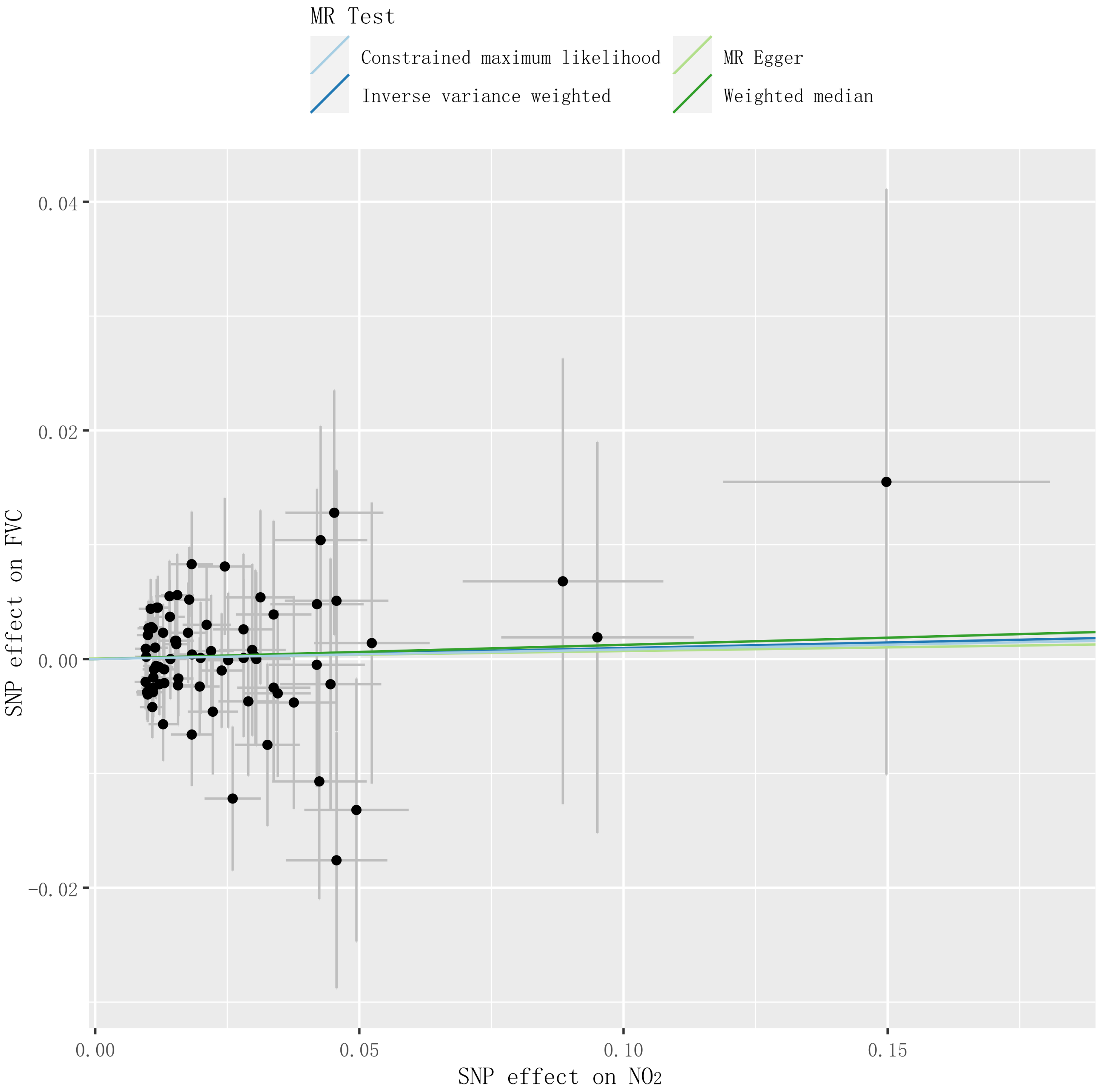
MR Test
Constrained maximum likelihood
Inverse variance weighted
MR Egger
Weighted median
0.04
0.02
0.00
−0.02
SNP effect on FVC
0.00
0.05
0.10
0.15
SNP effect on NO2

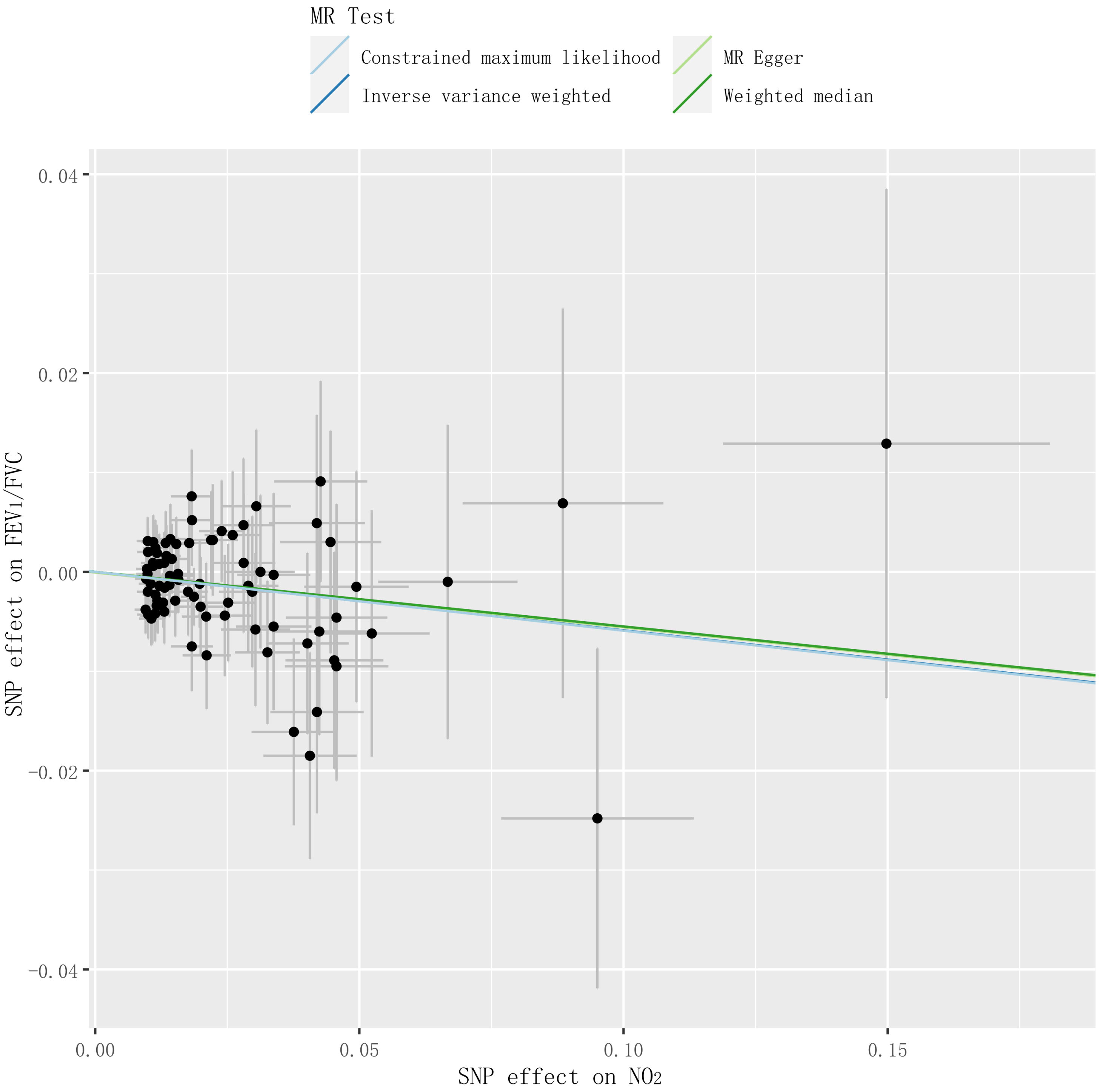

MR Test
Constrained maximum likelihood
Inverse variance weighted
MR Egger
Weighted median
0.04
0.02
0.00
−0.02
−0.04
SNP effect on FEV1/FVC
0.00
0.05
0.10
0.15
SNP effect on NO2

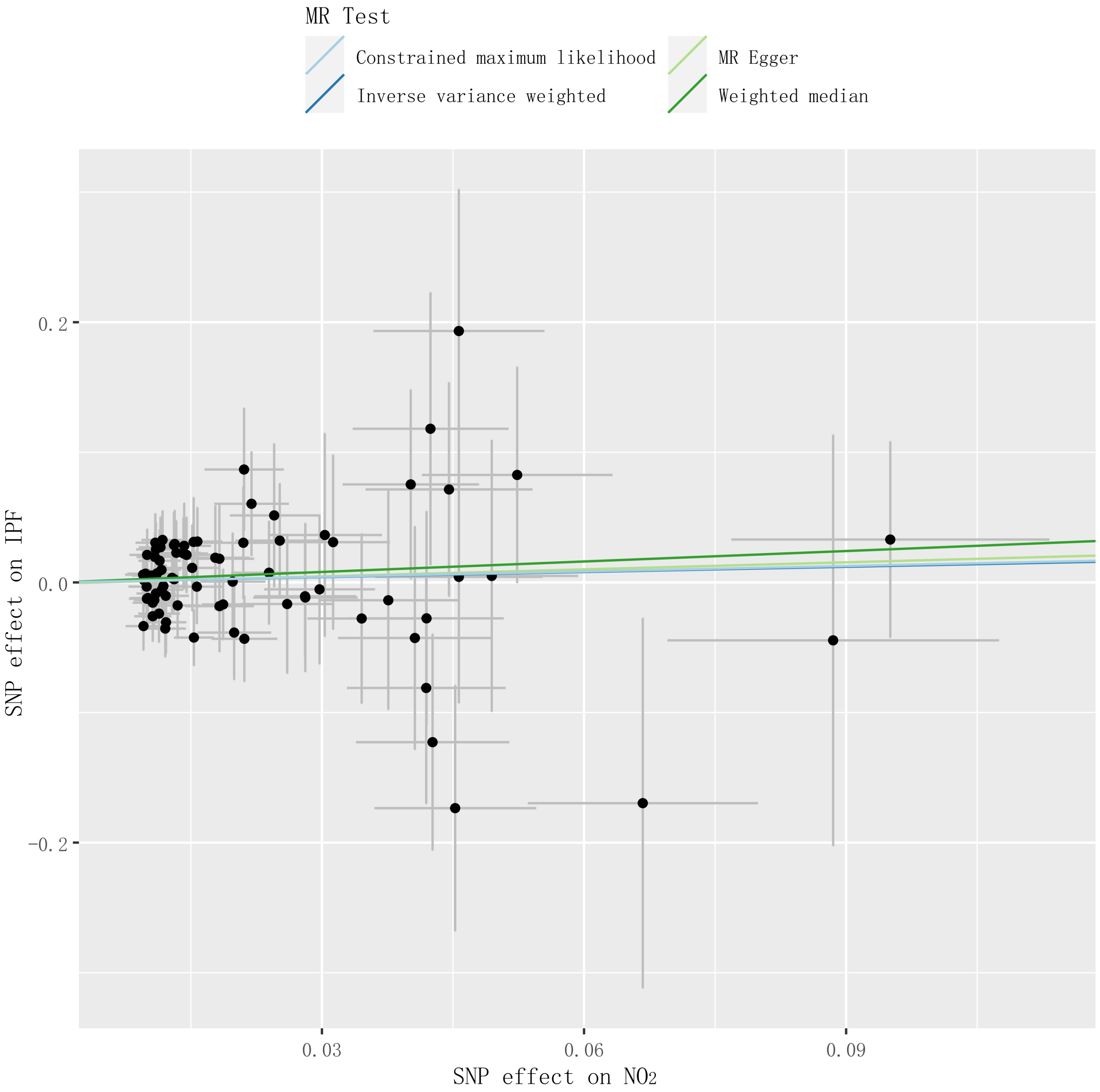

MR Test
Constrained maximum likelihood
Inverse variance weighted
MR Egger
Weighted median
0.2
0.0
-0.2
SNP effect on IPF
0.03
0.06
0.09
SNP effect on NO2

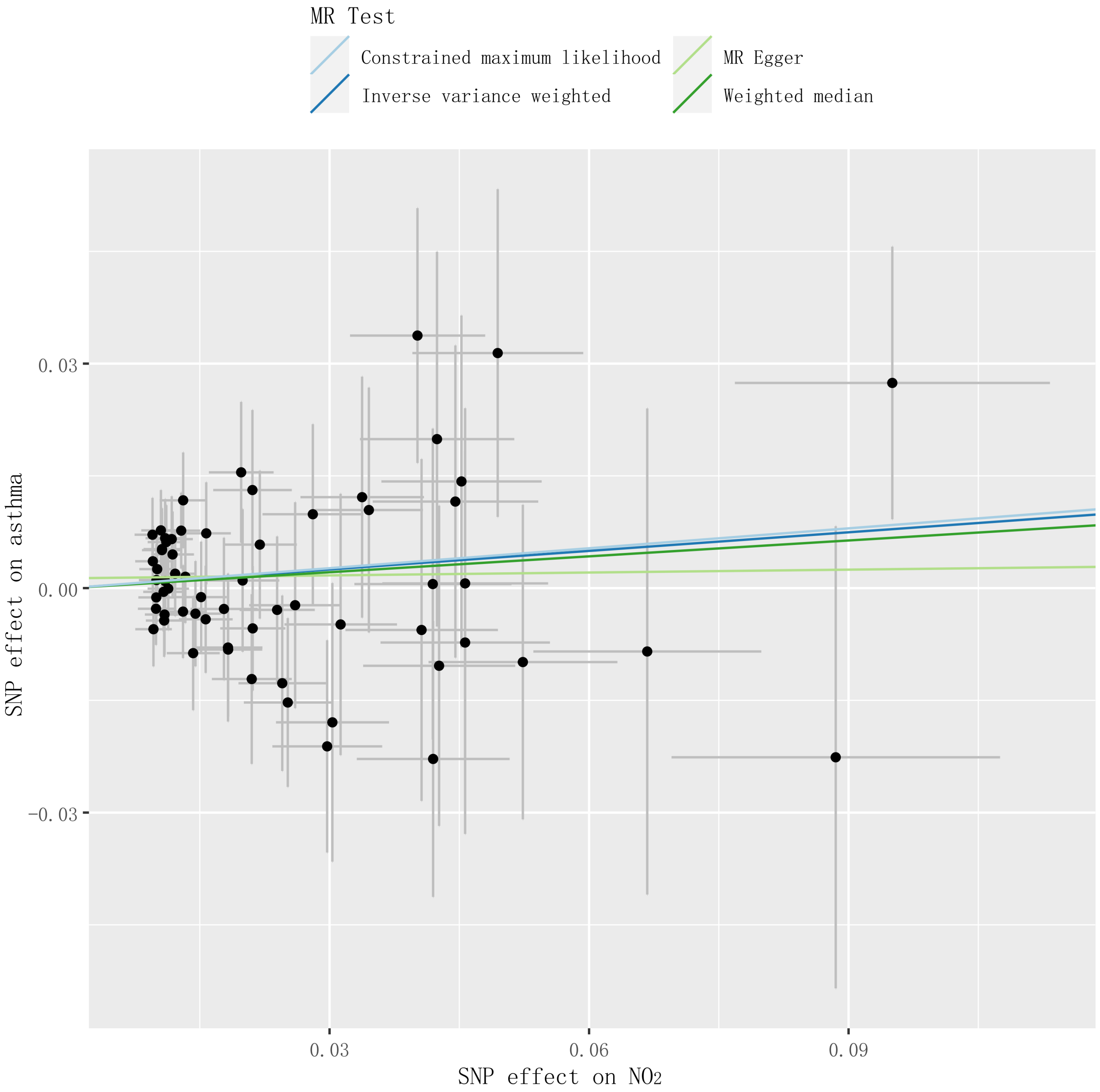
MR Test
Constrained maximum likelihood
Inverse variance weighted
MR Egger
Weighted median
0.03
0.00
-0.03
SNP effect on asthma
0.03
0.06
0.09
SNP effect on NO2

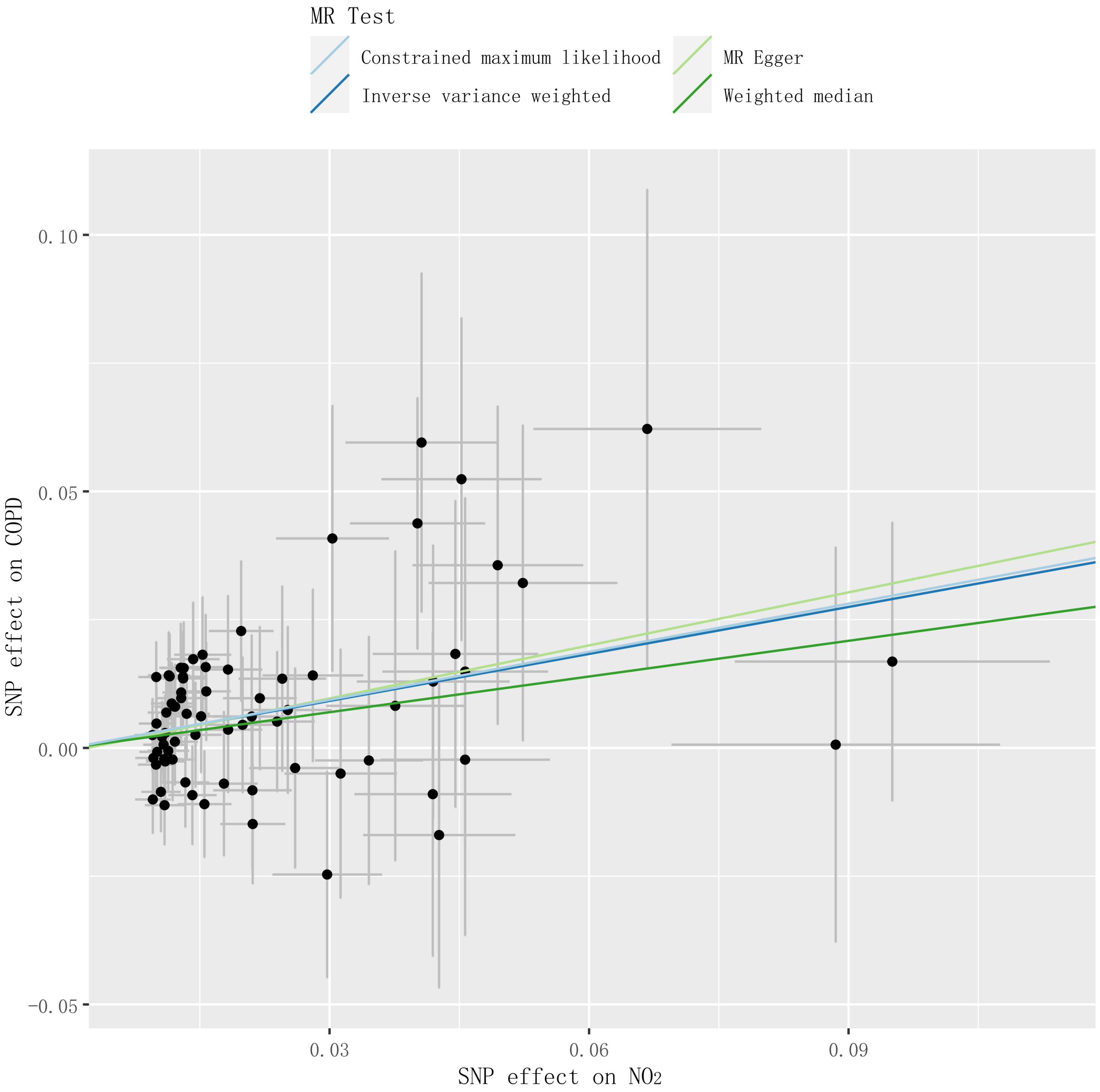
MR Test
Constrained maximum likelihood
Inverse variance weighted
MR Egger
Weighted median
0.10
0.05
0.00
−0.05
SNP effect on COPD
0.03
0.06
0.09
SNP effect on NO2

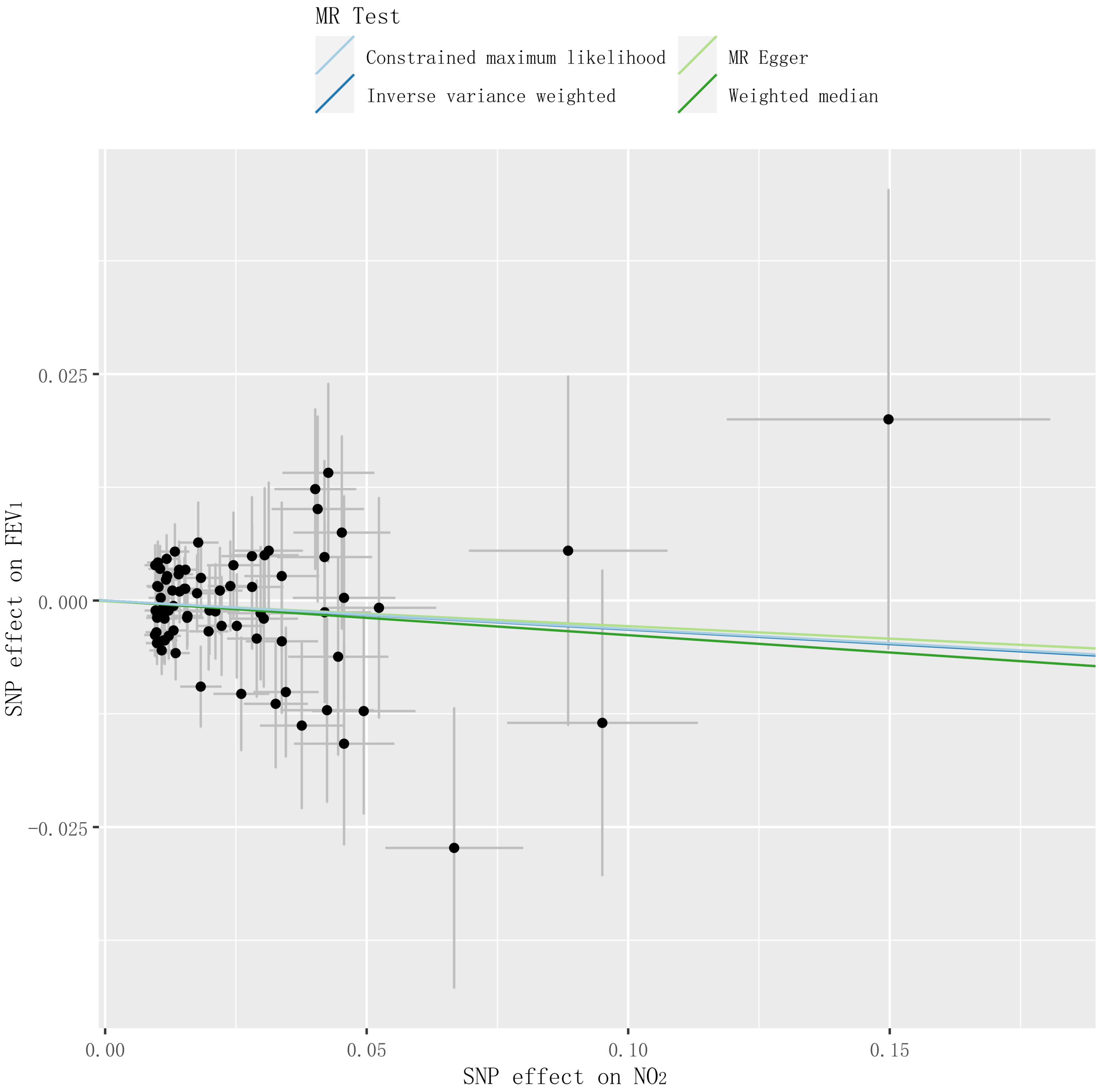

MR Test
Constrained maximum likelihood
Inverse variance weighted
MR Egger
Weighted median
0.025
0.000
−0.025
SNP effect on FEV1
0.00
0.05
0.10
0.15
SNP effect on NO2

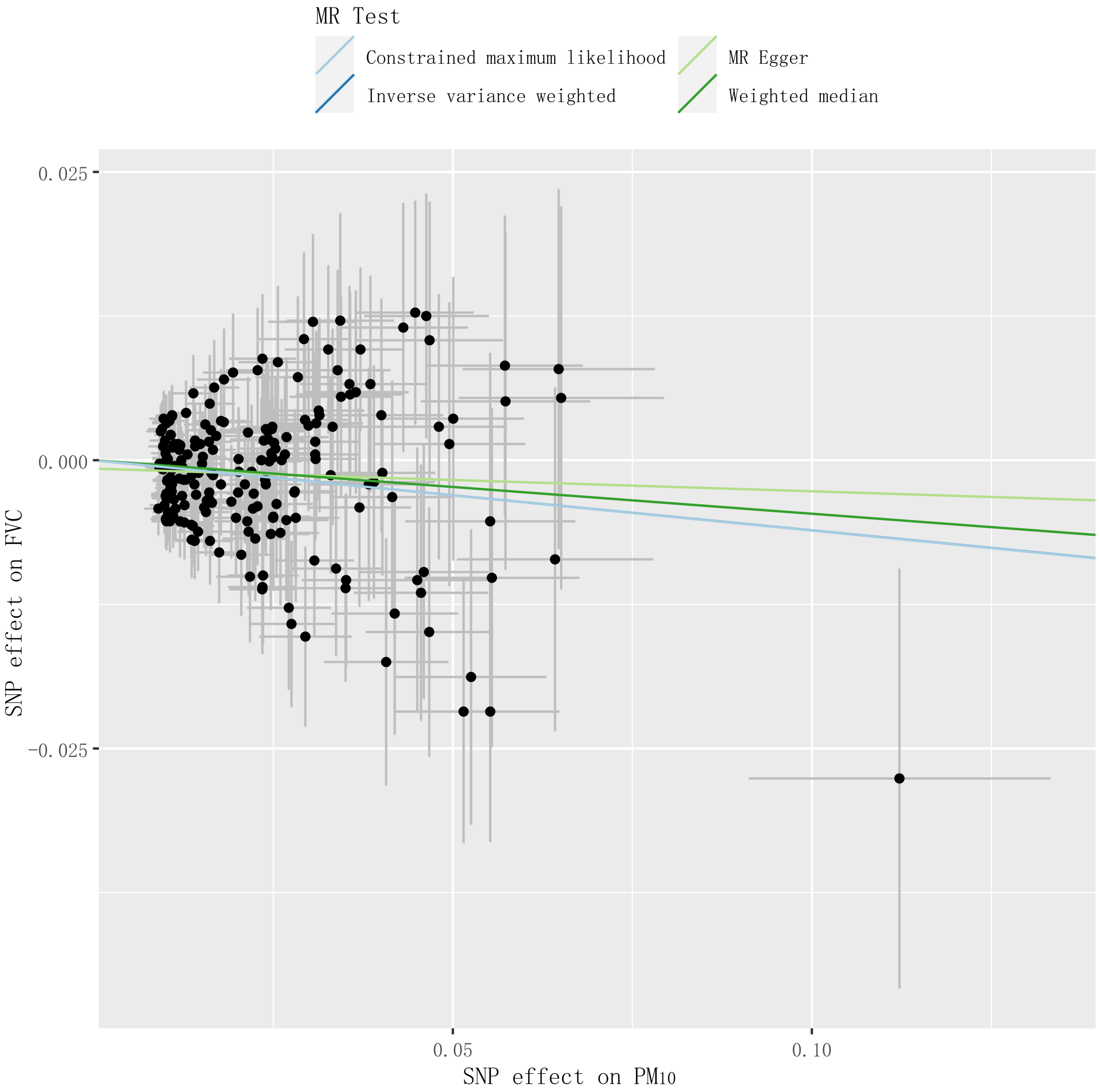

MR Test
Constrained maximum likelihood
Inverse variance weighted
MR Egger
Weighted median
0.025
0.000
−0.025
SNP effect on FVC
0.05
0.10
SNP effect on PM10

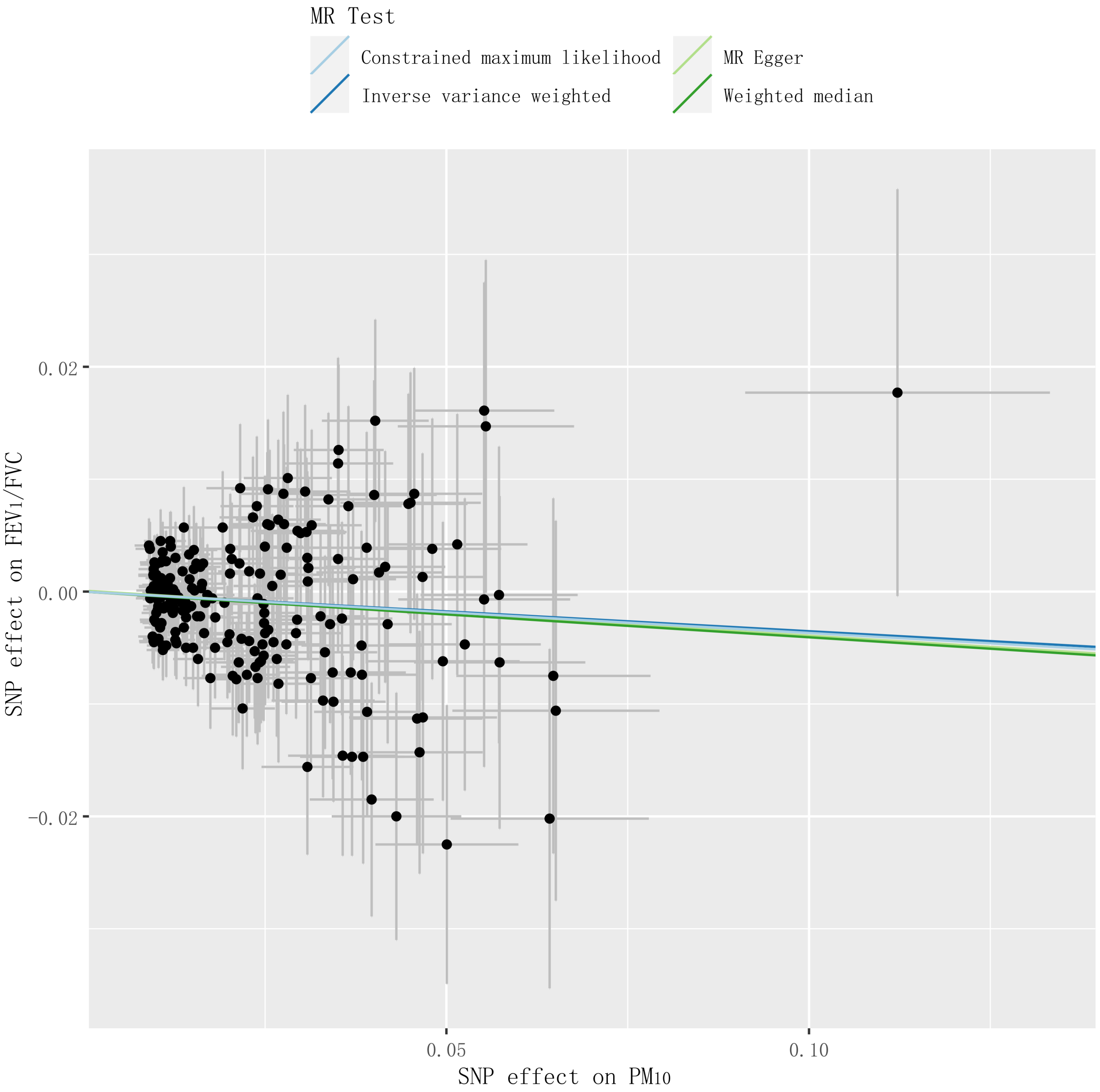

MR Test
Constrained maximum likelihood
Inverse variance weighted
MR Egger
Weighted median
0.02
0.00
-0.02
SNP effect on FEV1/FVC
0.05
0.10
SNP effect on PM10

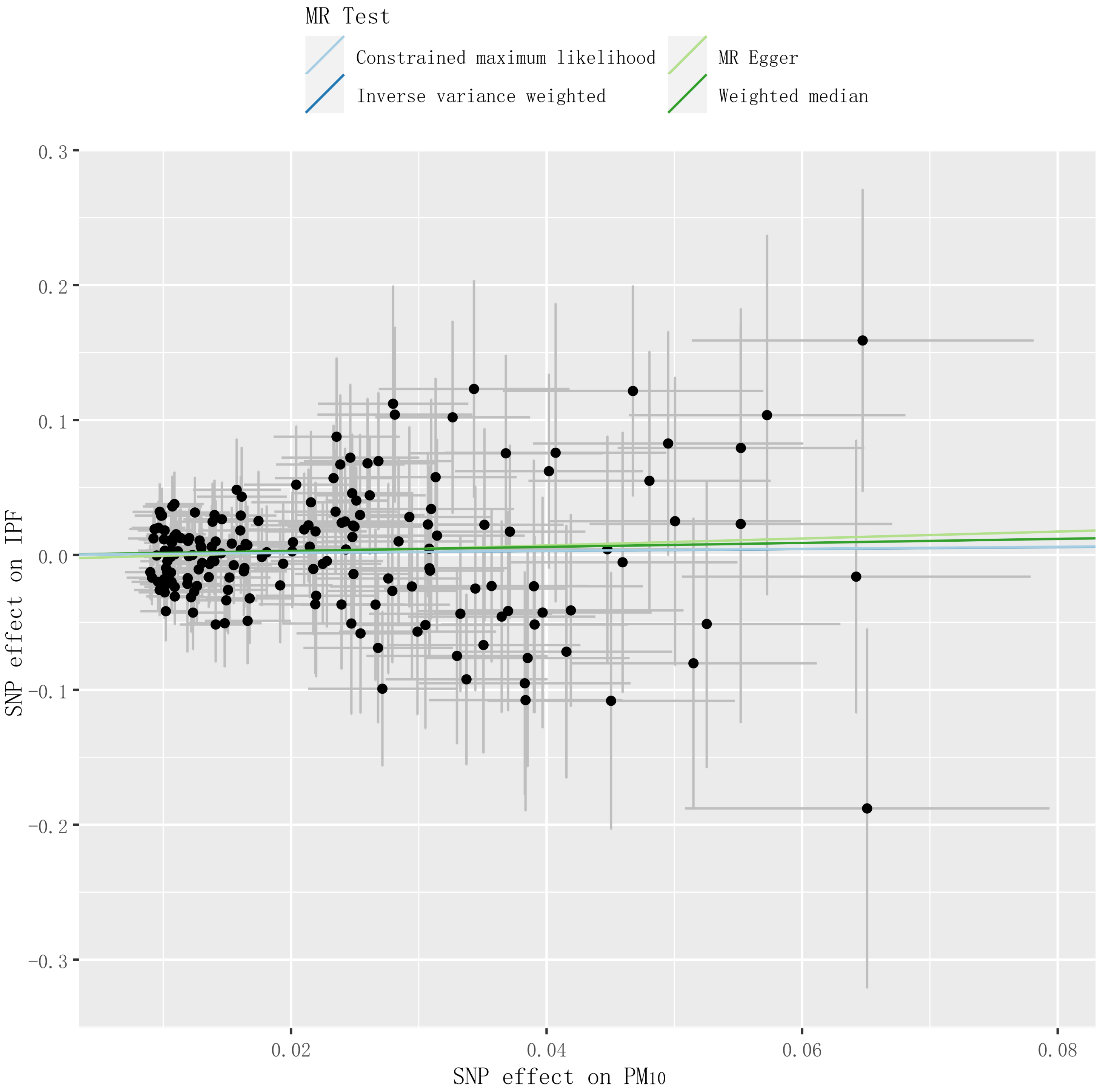

MR Test
Constrained maximum likelihood
Inverse variance weighted
MR Egger
Weighted median
0.3
0.2
0.1
0.0
−0.1
−0.2
−0.3
SNP effect on IPF
0.02
0.04
0.06
0.08
SNP effect on PM10

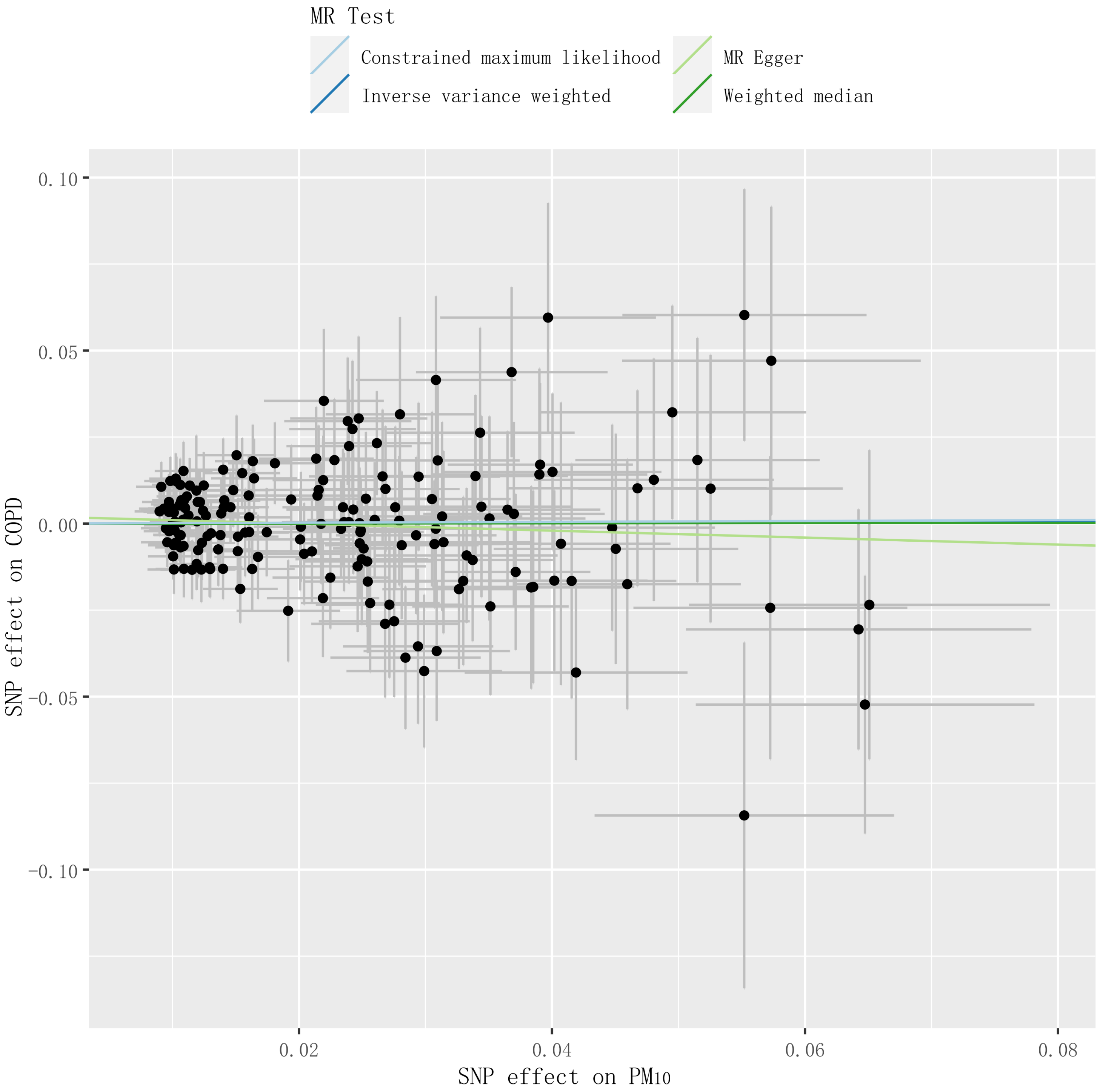
MR Test
Constrained maximum likelihood
Inverse variance weighted
MR Egger
Weighted median
0.10
0.05
0.00
−0.05
−0.10
SNP effect on COPD
0.02
0.04
0.06
0.08
SNP effect on PM10

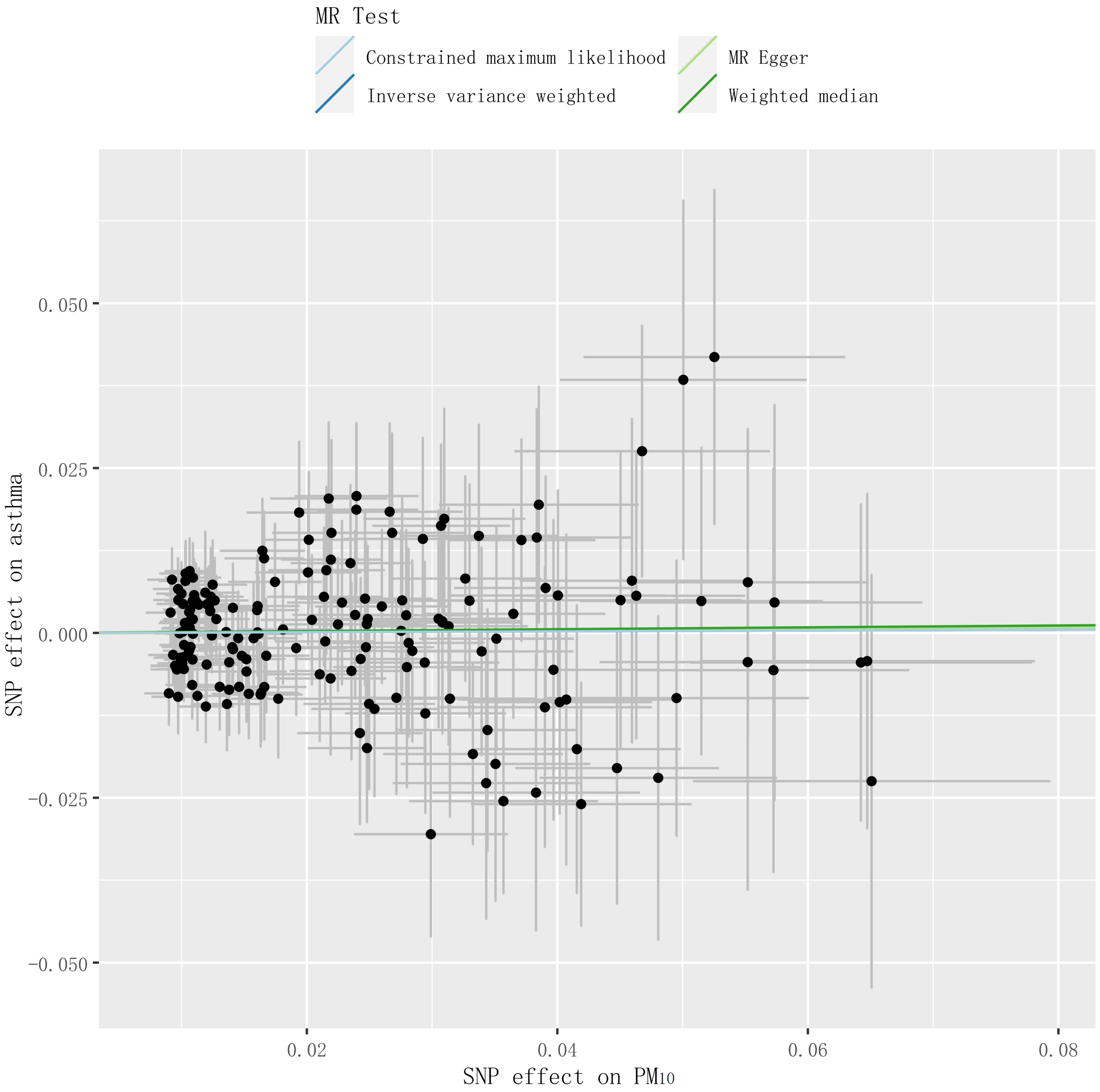

MR Test
Constrained maximum likelihood
Inverse variance weighted
MR Egger
Weighted median
0.050
0.025
0.000
−0.025
−0.050
SNP effect on asthma
0.02
0.04
0.06
0.08
SNP effect on PM10

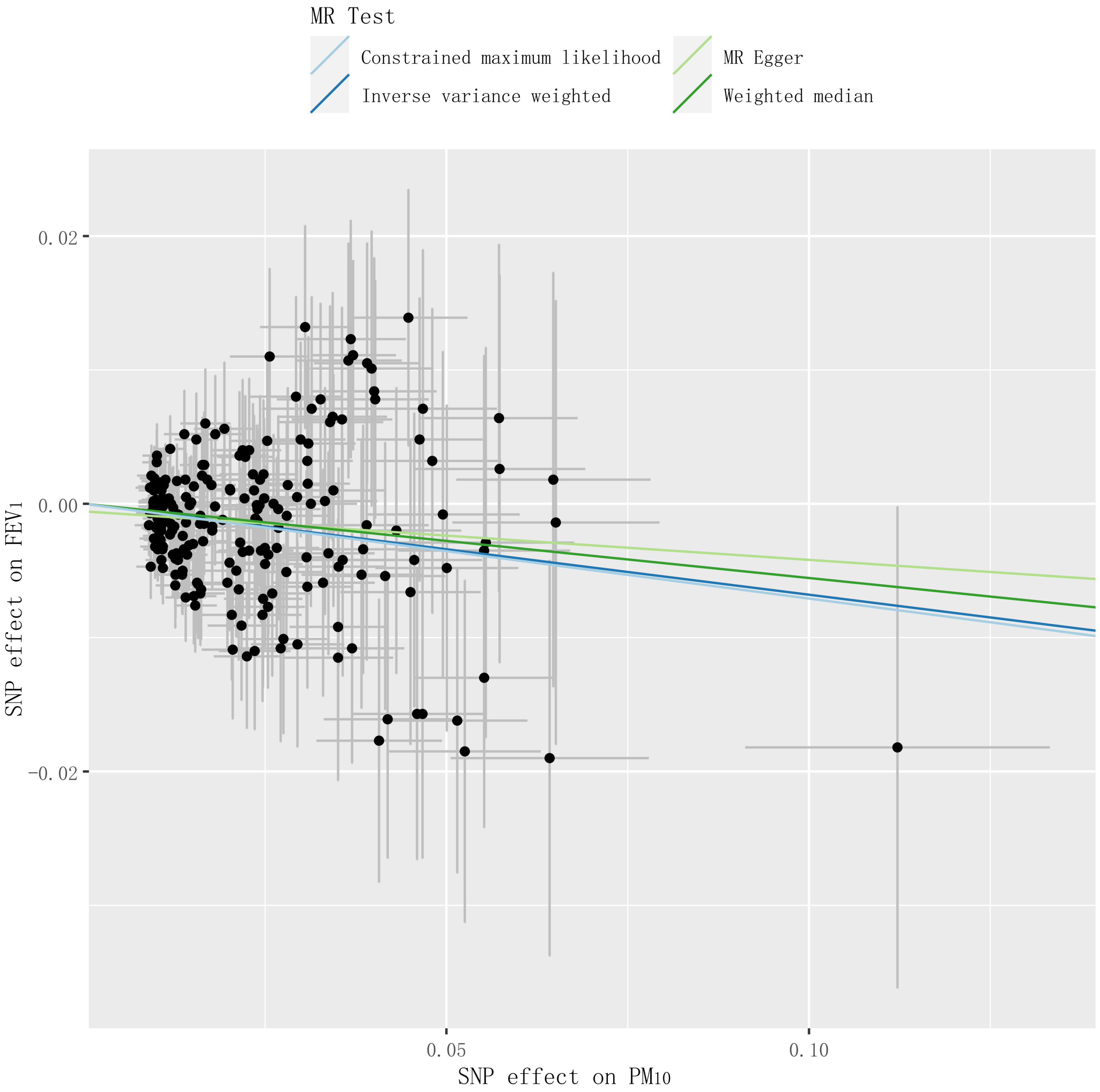

MR Test
Constrained maximum likelihood
Inverse variance weighted
MR Egger
Weighted median
0.02
0.00
−0.02
SNP effect on FEV1
0.05
0.10
SNP effect on PM10

Figure S2.Funnel plot of air pollution with CRDs and lung function

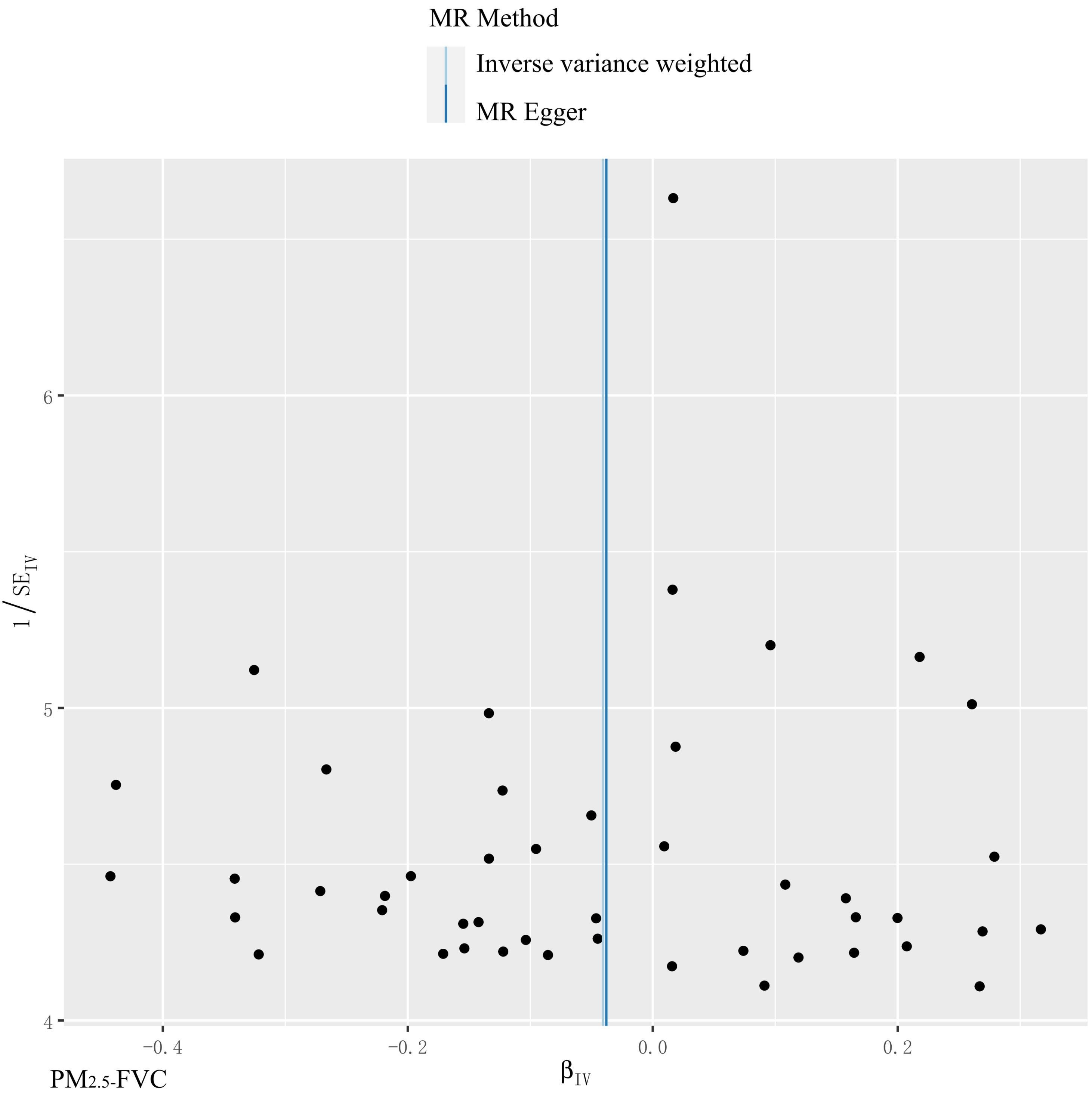

PM2.5-FVC

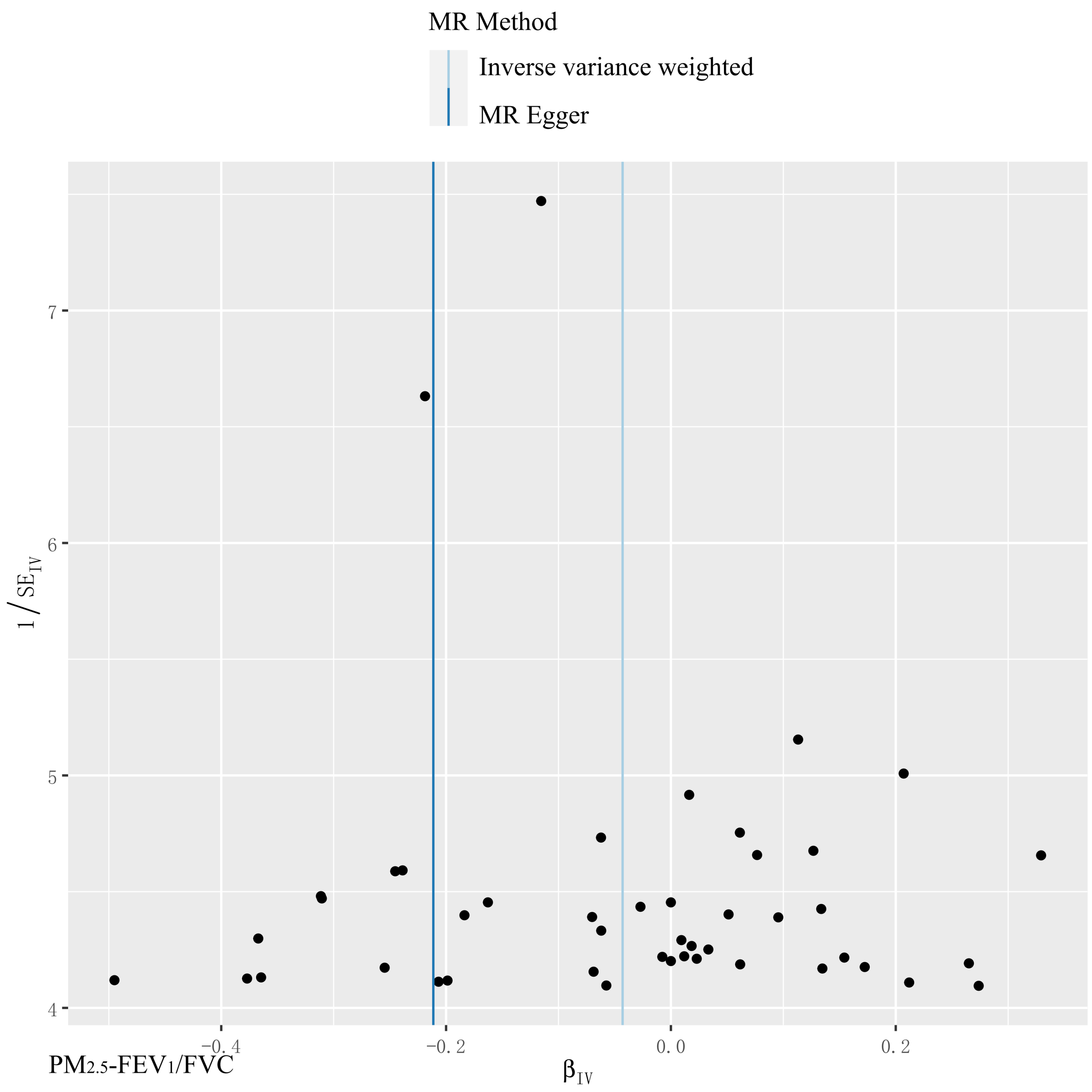

PM2.5-FEV1/FVC

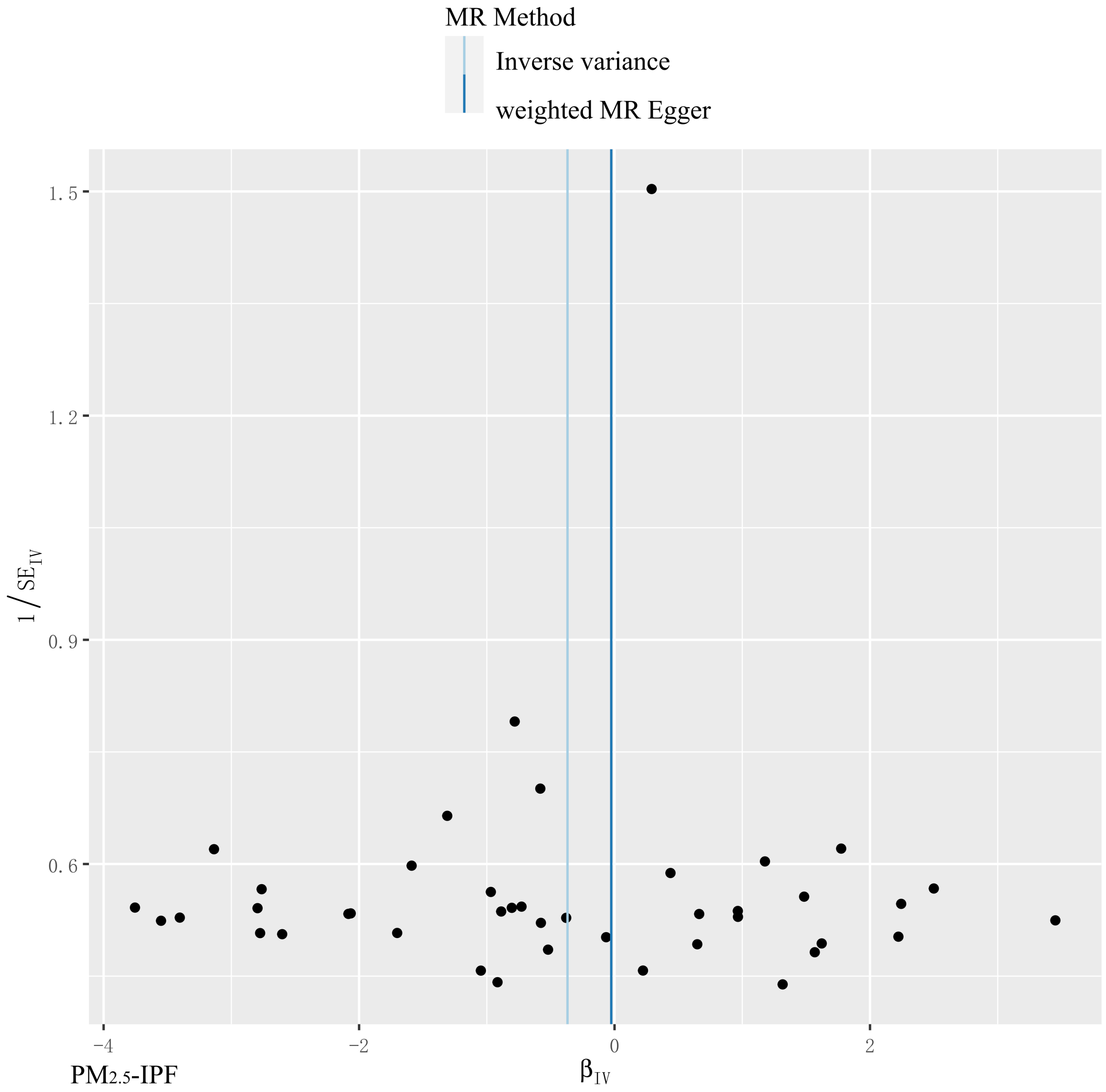

MR Method
Inverse variance
weighted MR Egger
1.5
1.2
0.9
0.6
1/SE_IV
−4
−2
0
2
β_IV

PM2.5-IPF

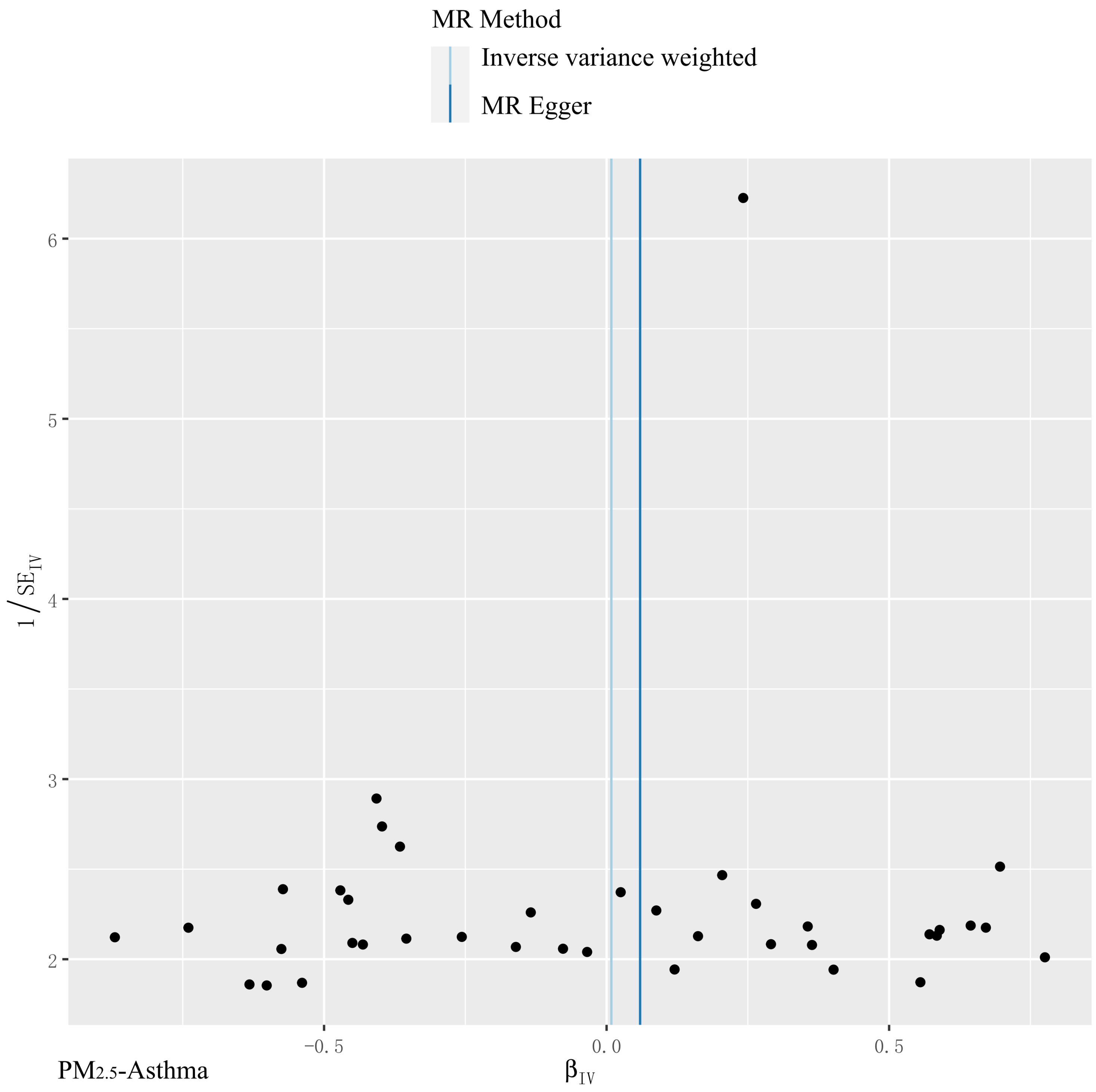
MR Method
Inverse variance weighted
MR Egger
6
5
4
3
2
$1/SE_{IV}$
−0.5
0.0
0.5
β_{IV}

PM2.5-Asthma

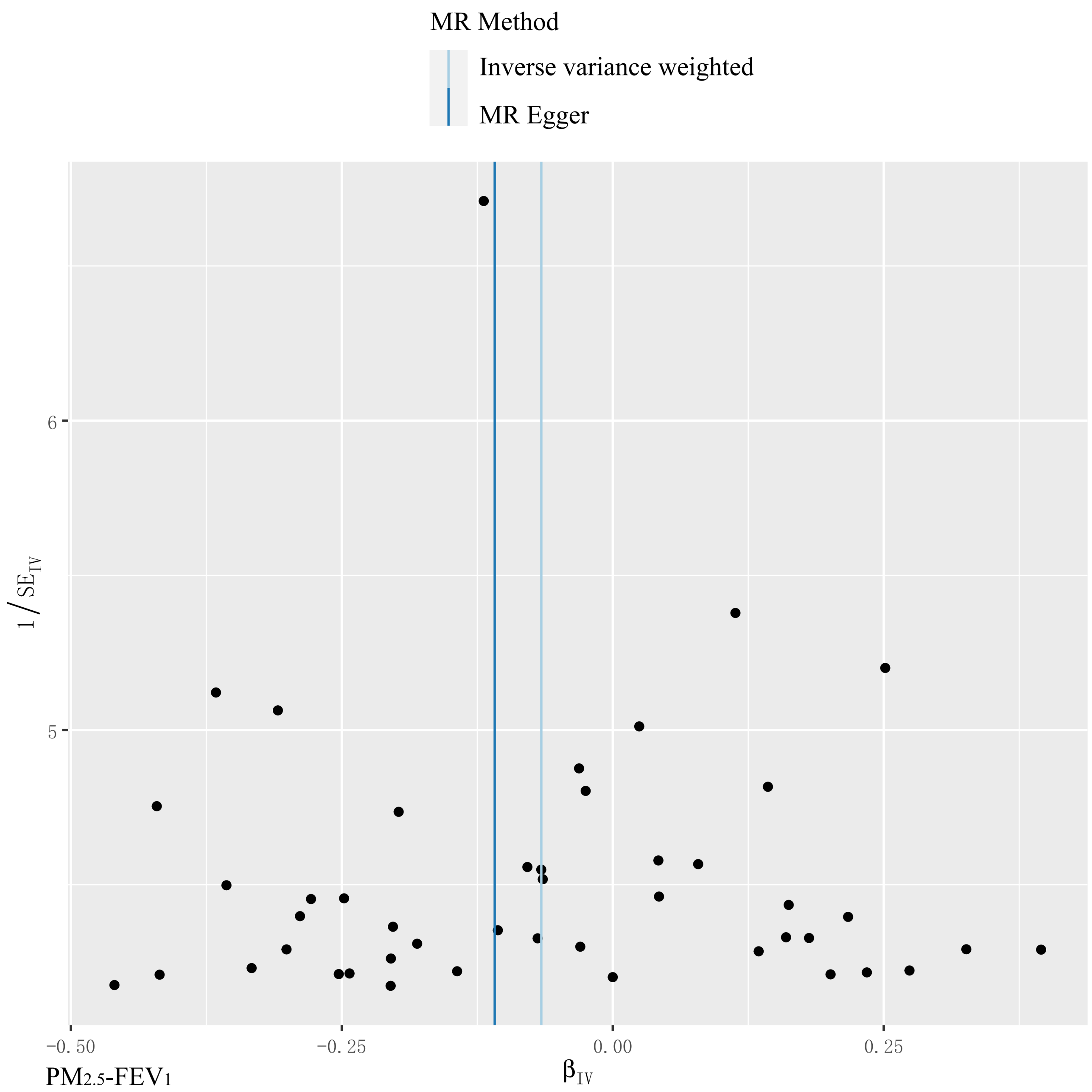

MR Method
Inverse variance weighted
MR Egger
6
5
1/SE_IV
−0.50
−0.25
0.00
0.25
β_IV

PM2.5-FEV1

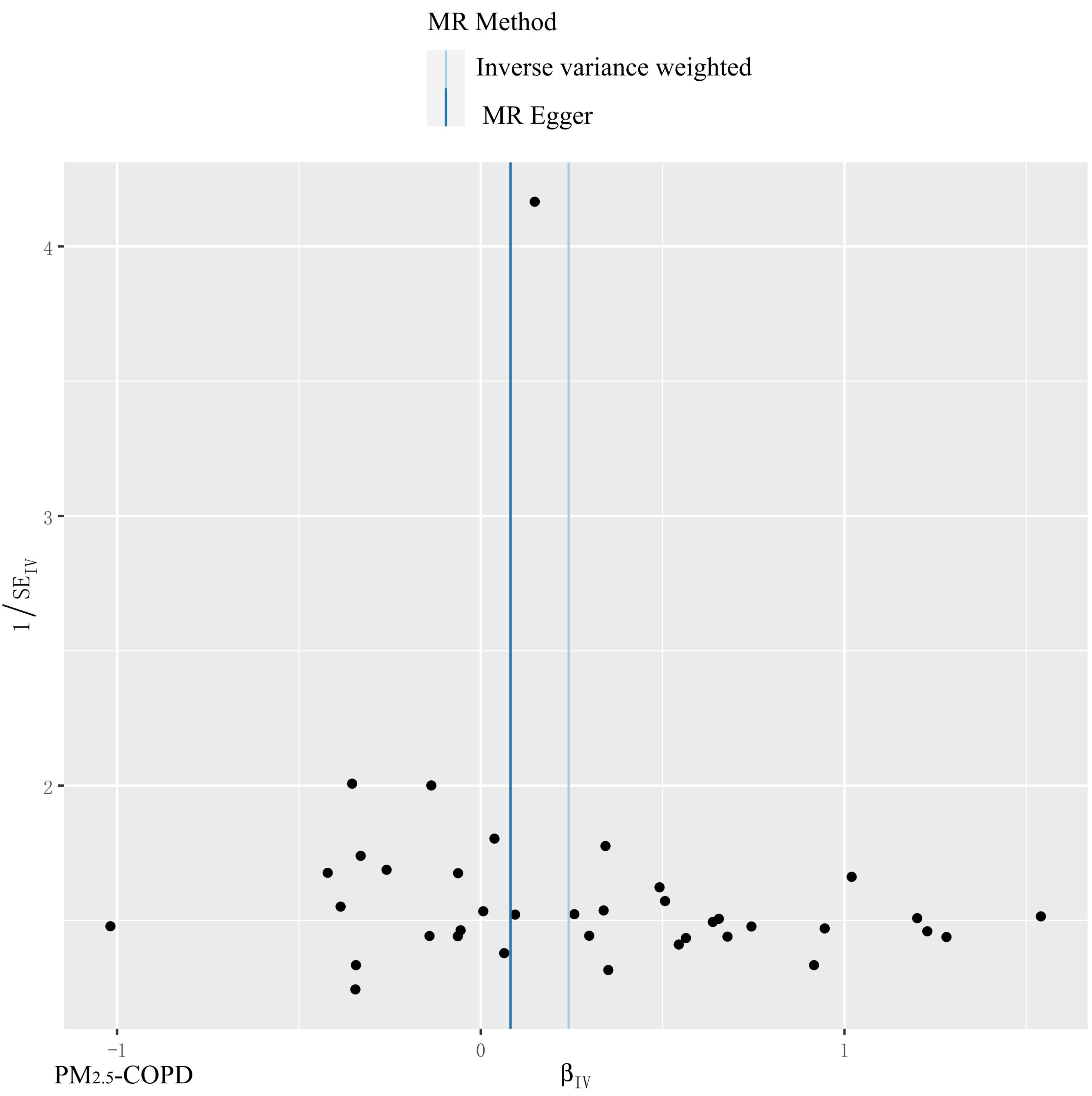
MR Method
Inverse variance weighted
MR Egger
4
3
2
1/SE$_{IV}$
−1
0
1
β$_{IV}$
PM2.5-COPD

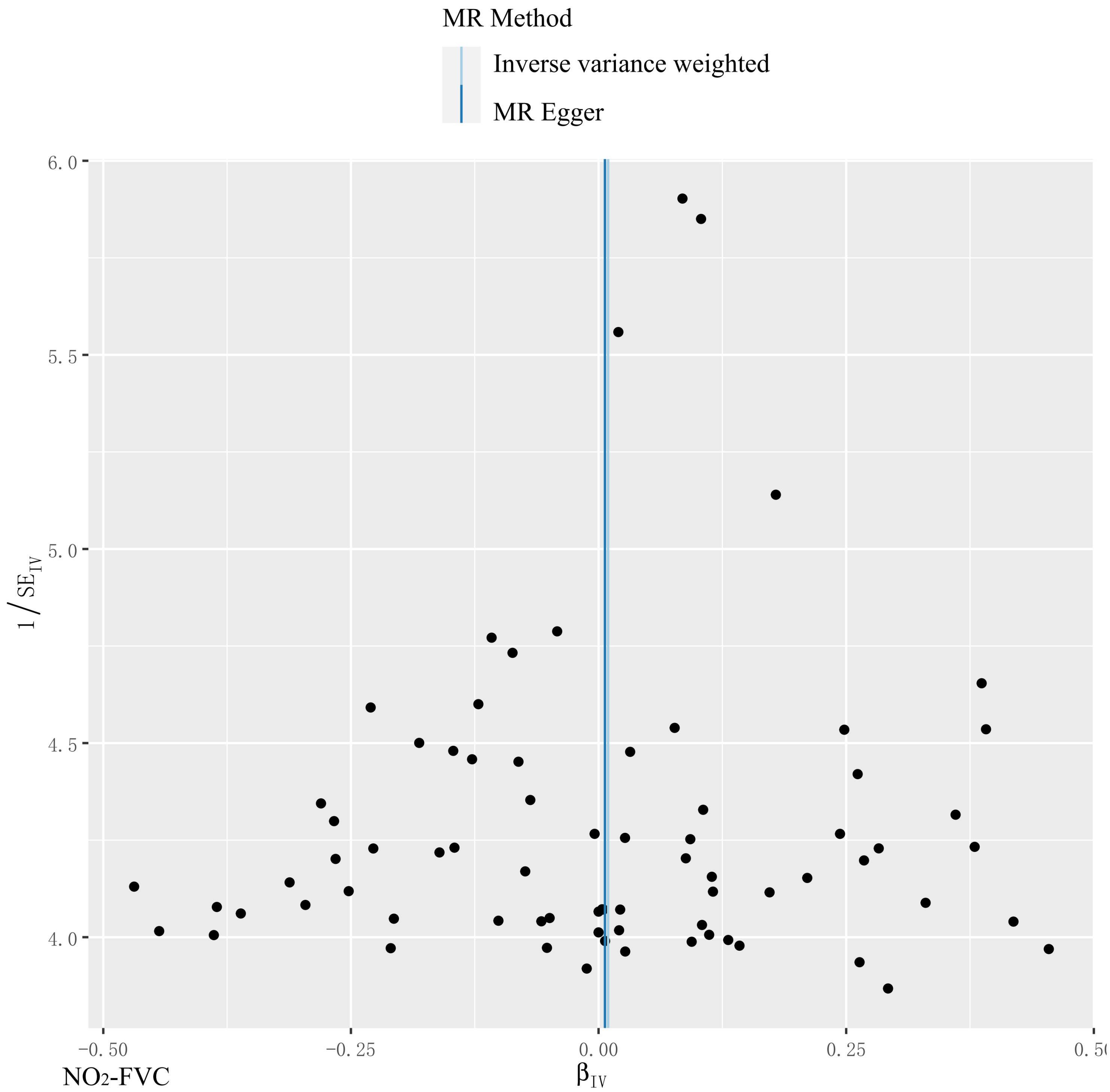

MR Method
Inverse variance weighted
MR Egger
6.0
5.5
5.0
4.5
4.0
$1/SE_{IV}$
−0.50
−0.25
0.00
0.25
0.5
β_{IV}

NO_2-FVC

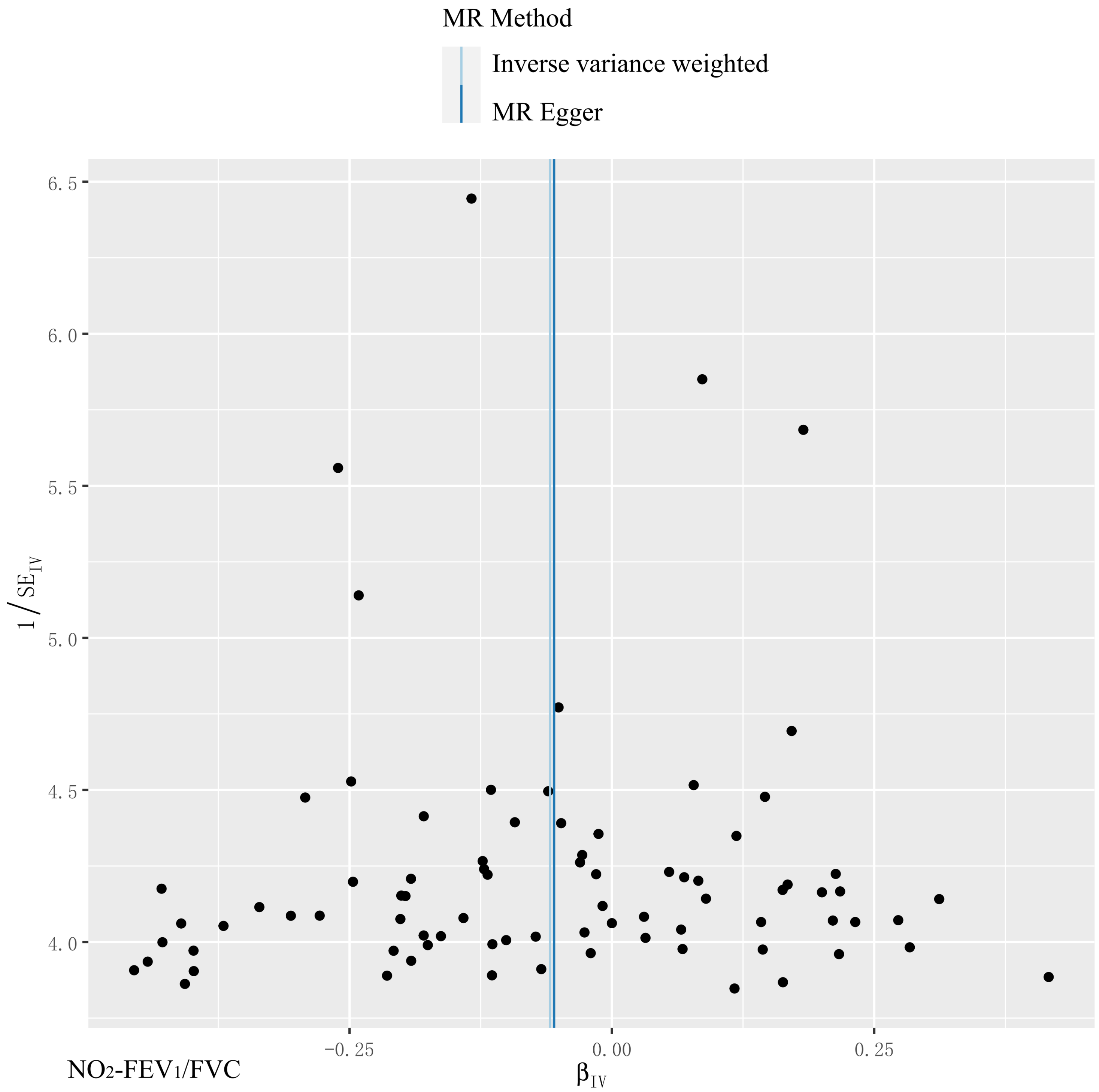
MR Method
Inverse variance weighted
MR Egger
6.5
6.0
5.5
5.0
4.5
4.0
1/SE_IV
-0.25
0.00
0.25
β_IV
NO2-FEV1/FVC

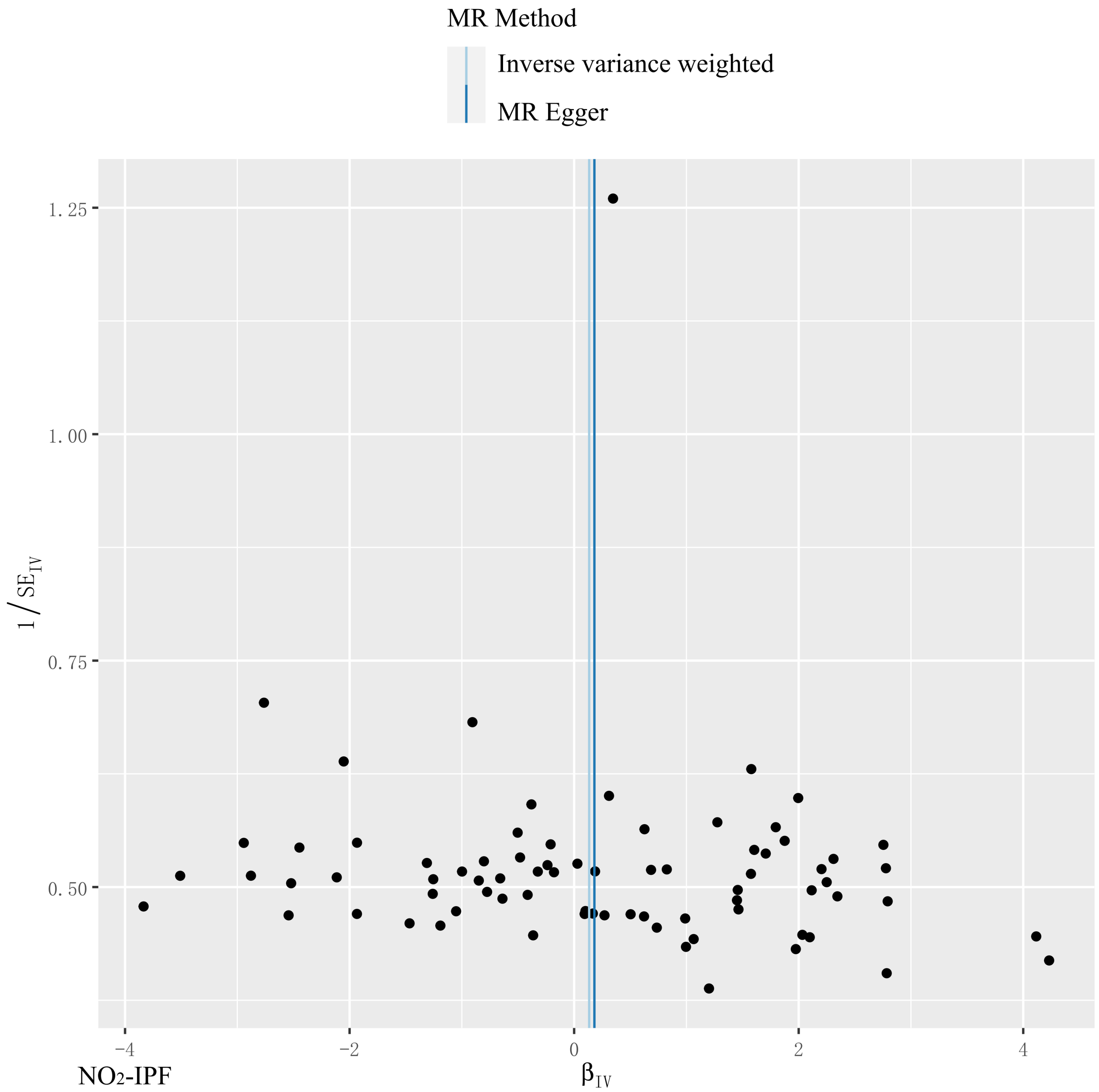
MR Method
Inverse variance weighted
MR Egger
1.25
1.00
0.75
0.50
1/SE_IV
−4
−2
0
2
4
β_IV
NO2-IPF

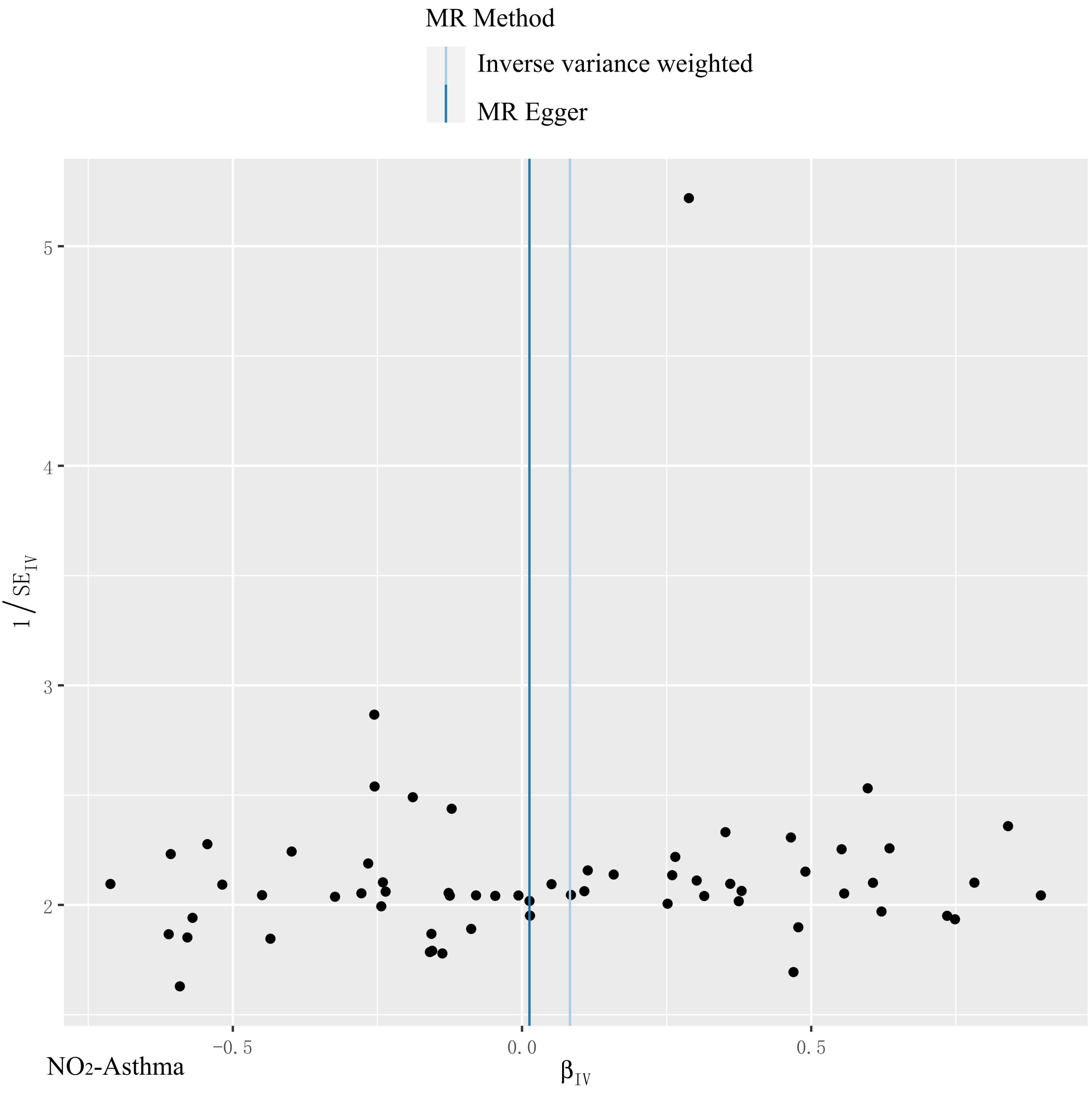

MR Method
Inverse variance weighted
MR Egger
5
4
3
2
1/SE_IV
−0.5
0.0
0.5
β_IV

NO2-Asthma

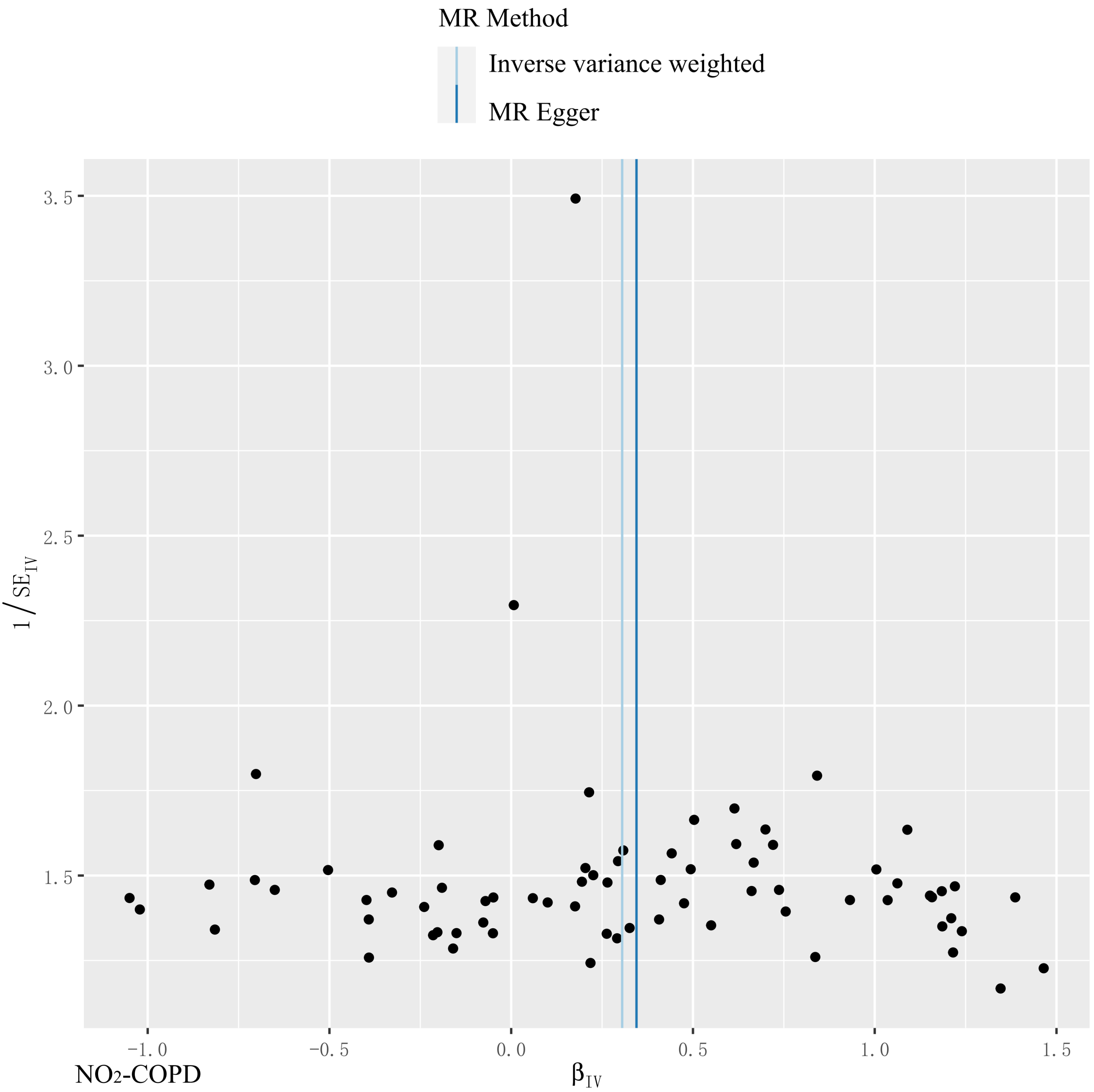
MR Method
Inverse variance weighted
MR Egger
3.5
3.0
2.5
2.0
1.5
1/SE_IV
-1.0
-0.5
0.0
0.5
1.0
1.5
β_IV
NO2-COPD

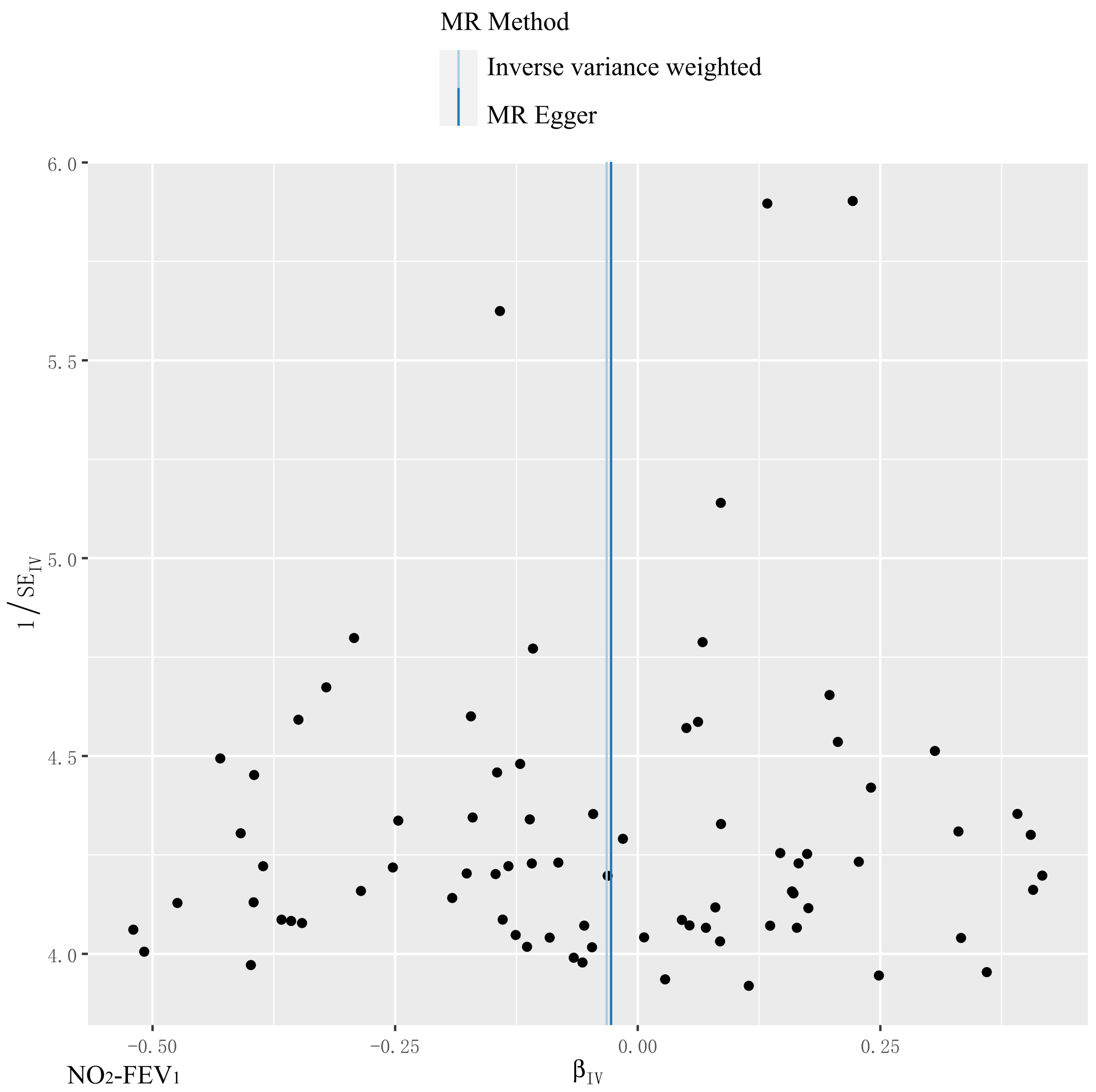

MR Method
Inverse variance weighted
MR Egger
6.0
5.5
5.0
4.5
4.0
1/SE_IV
−0.50
−0.25
0.00
0.25
β_IV
NO2-FEV1

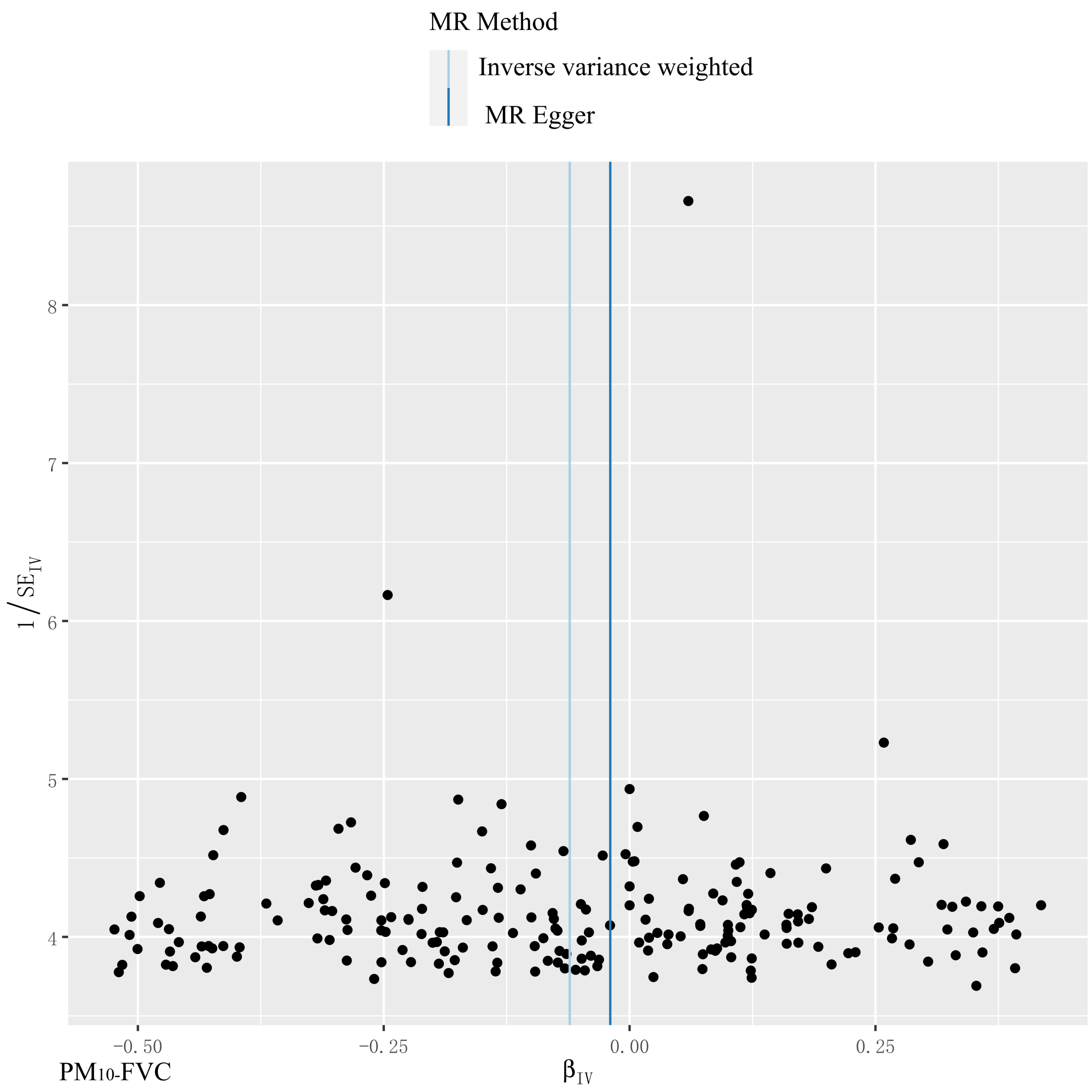
MR Method
Inverse variance weighted
MR Egger
1/SE_IV
8
7
6
5
4
−0.50
−0.25
0.00
0.25
β_IV
PM10-FVC

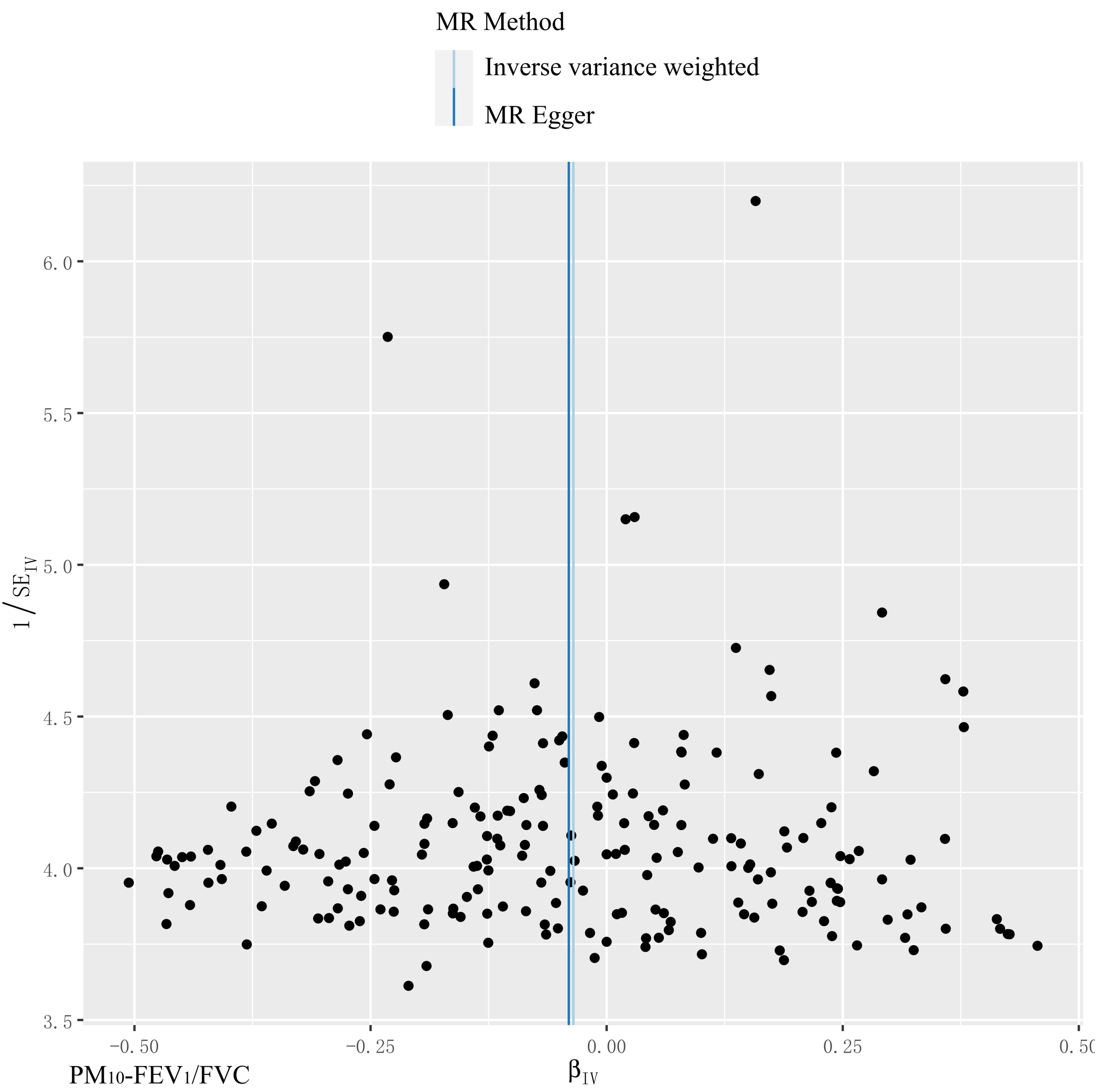
MR Method
Inverse variance weighted
MR Egger
6.0
5.5
5.0
4.5
4.0
3.5
1/SE_IV
−0.50
−0.25
0.00
0.25
0.50
β_IV
PM10-FEV1/FVC

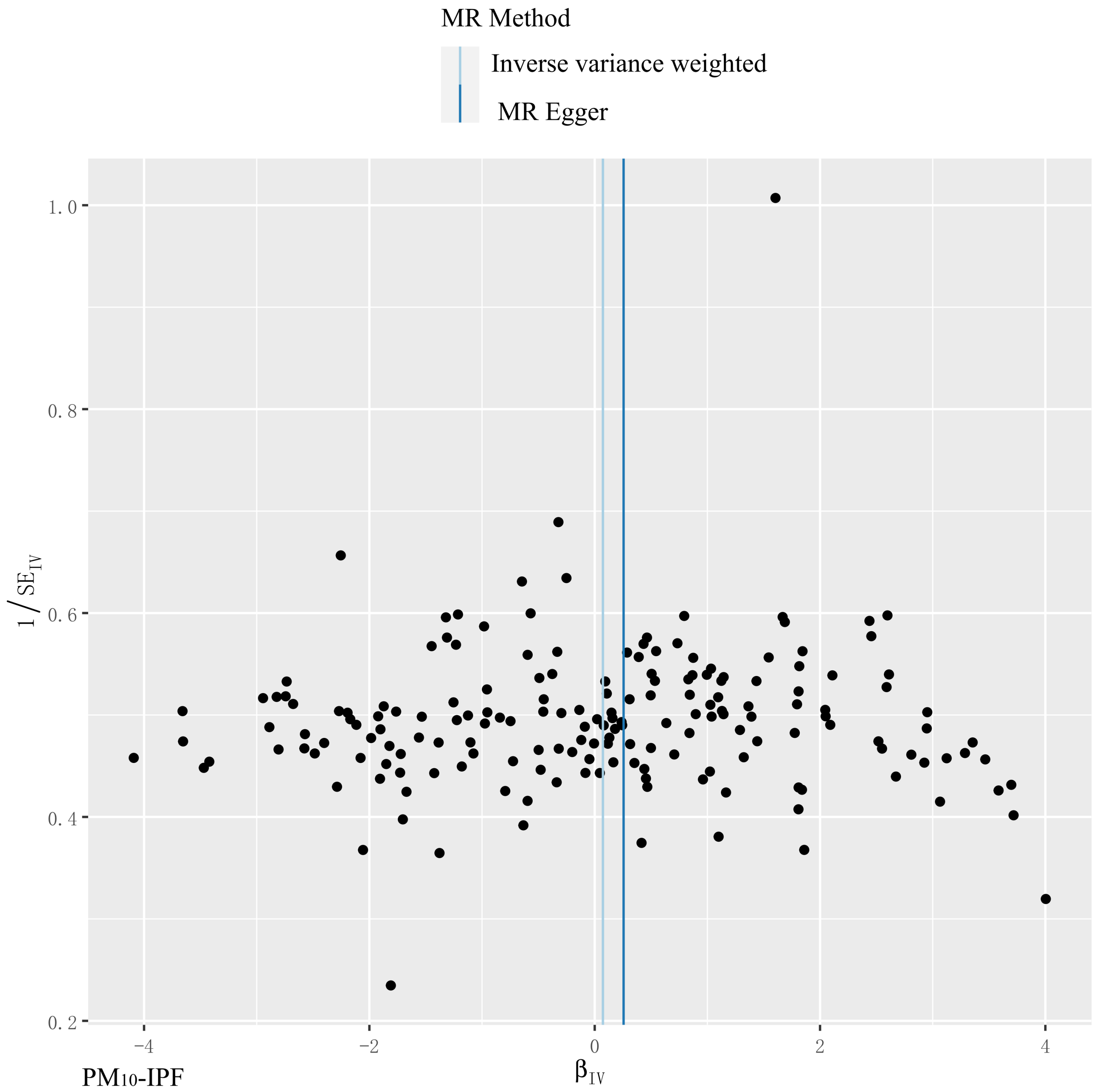
MR Method
Inverse variance weighted
MR Egger
1.0
0.8
0.6
0.4
0.2
−4
−2
0
2
4
1/SE_IV
β_IV
PM10-IPF

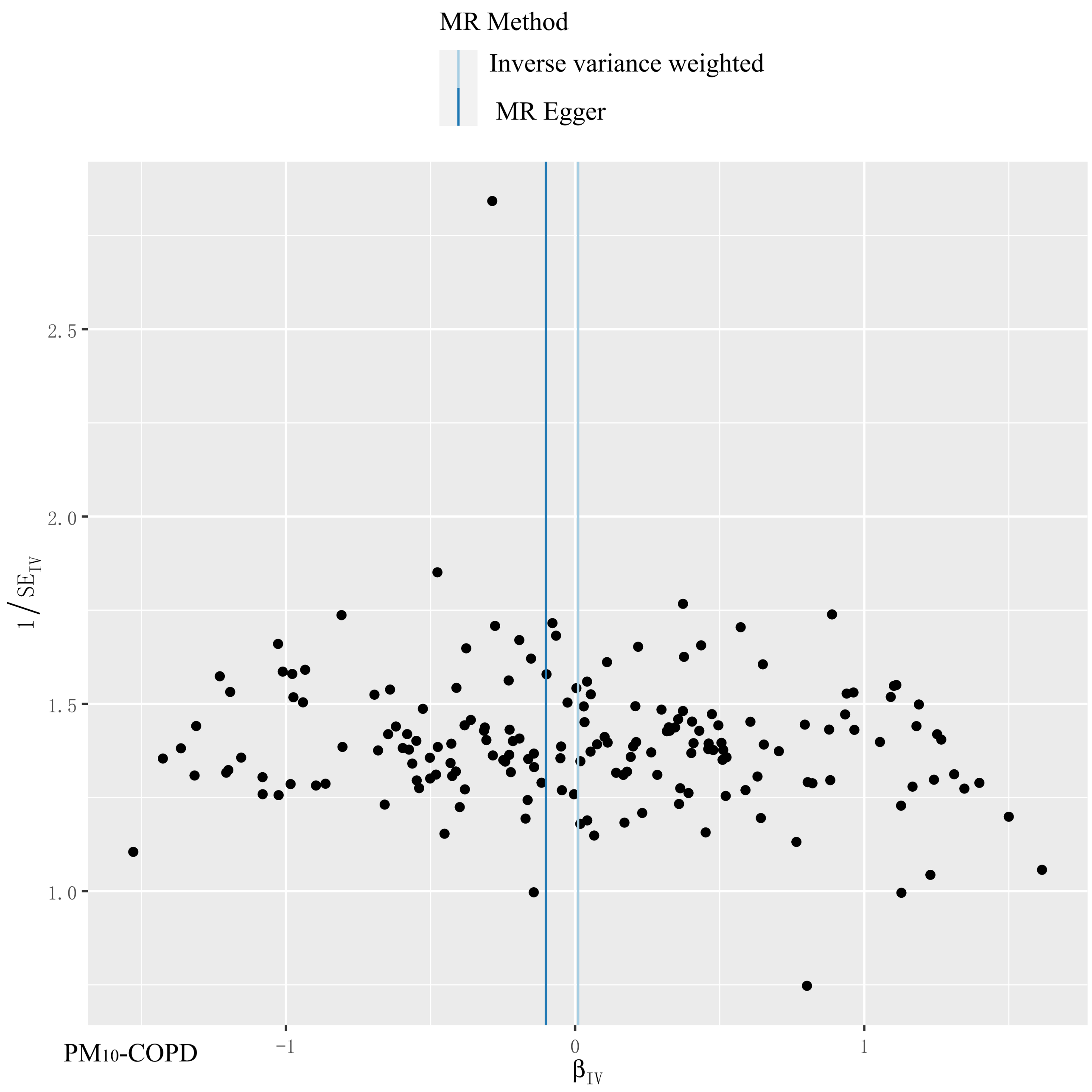

MR Method
Inverse variance weighted
MR Egger
2.5
2.0
1.5
1.0
1/SE_IV
−1
0
1
β_IV

PM10-COPD

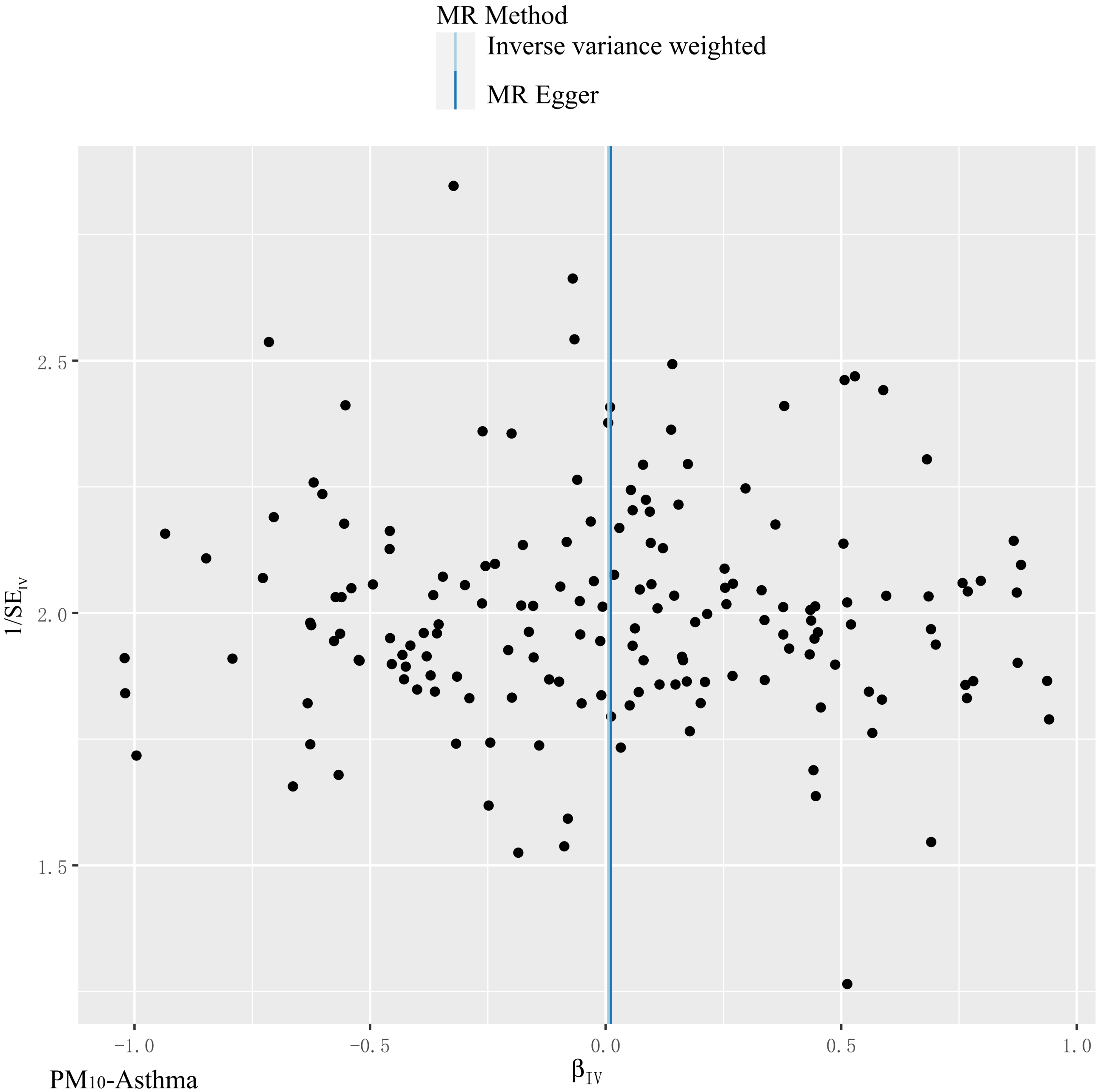
MR Method
Inverse variance weighted
MR Egger
2.5
2.0
1.5
1/SE_IV
−1.0
−0.5
0.0
0.5
1.0
β_IV
PM10-Asthma

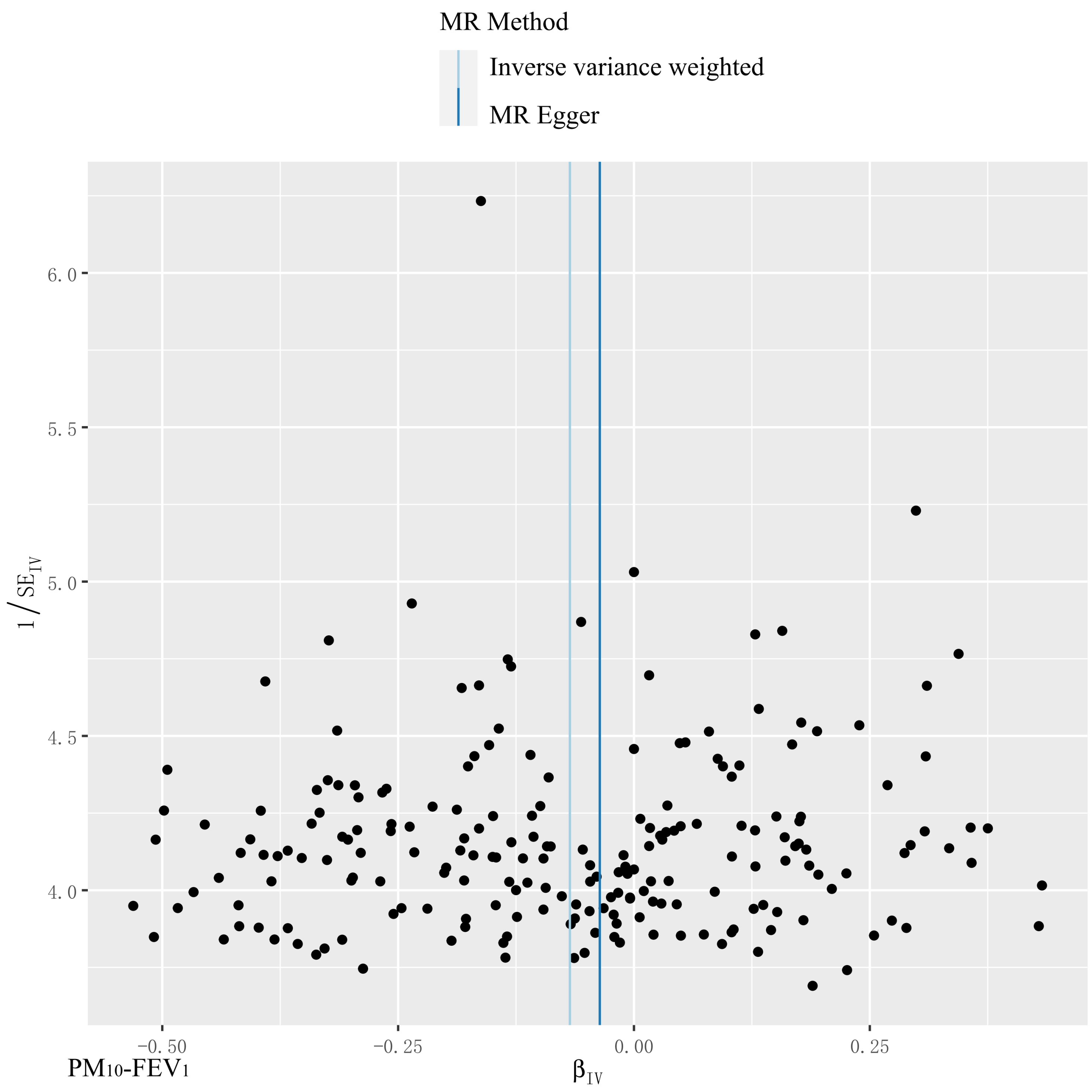
MR Method
Inverse variance weighted
MR Egger
6.0
5.5
5.0
4.5
4.0
1/SE_IV
−0.50
−0.25
0.00
0.25
β_IV

PM10-FEV1

Figure S3. leave-one-out analysis of air pollution with CRDs and lung function

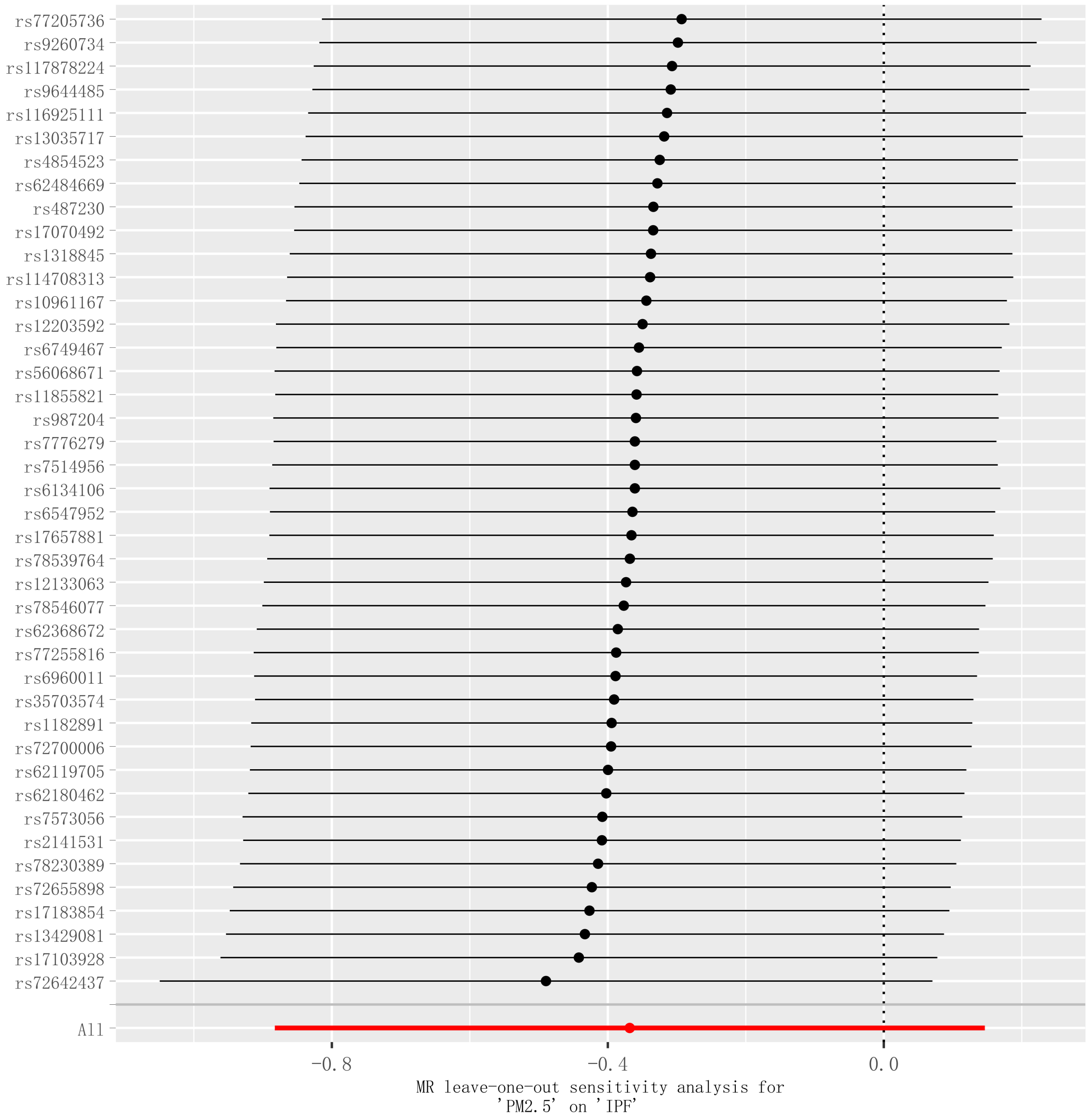

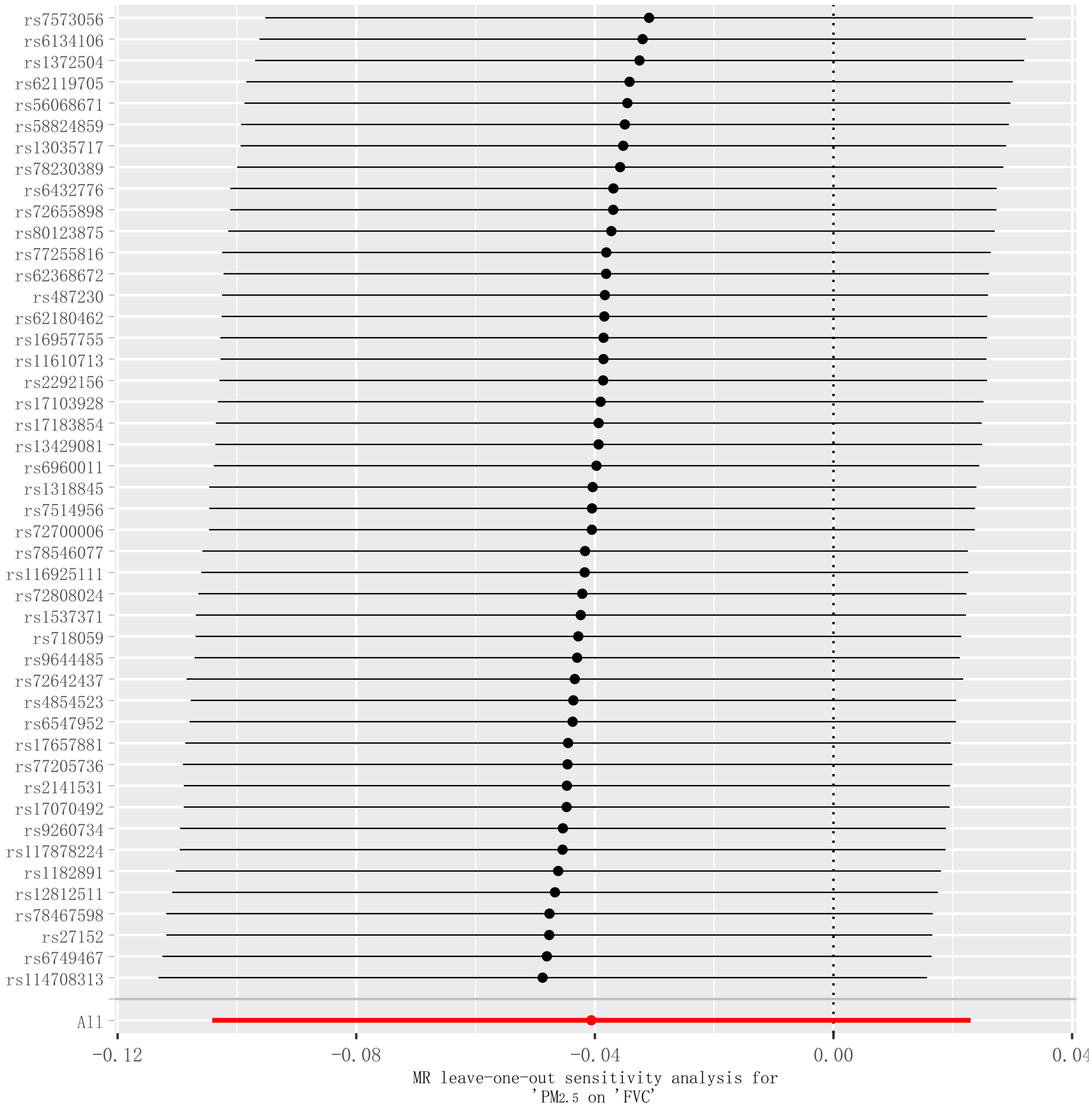

rs7573056
rs6134106
rs1372504
rs62119705
rs56068671
rs58824859
rs13035717
rs78230389
rs6432776
rs72655898
rs80123875
rs77255816
rs62368672
rs487230
rs62180462
rs16957755
rs11610713
rs2292156
rs17103928
rs17183854
rs13429081
rs6960011
rs1318845
rs7514956
rs72700006
rs78546077
rs116925111
rs72808024
rs1537371
rs718059
rs9644485
rs72642437
rs4854523
rs6547952
rs17657881
rs77205736
rs2141531
rs17070492
rs9260734
rs117878224
rs1182891
rs12812511
rs78467598
rs27152
rs6749467
rs114708313
All
-0.12
-0.08
-0.04
0.00
0.04
MR leave-one-out sensitivity analysis for
'PM2.5 on 'FVC'

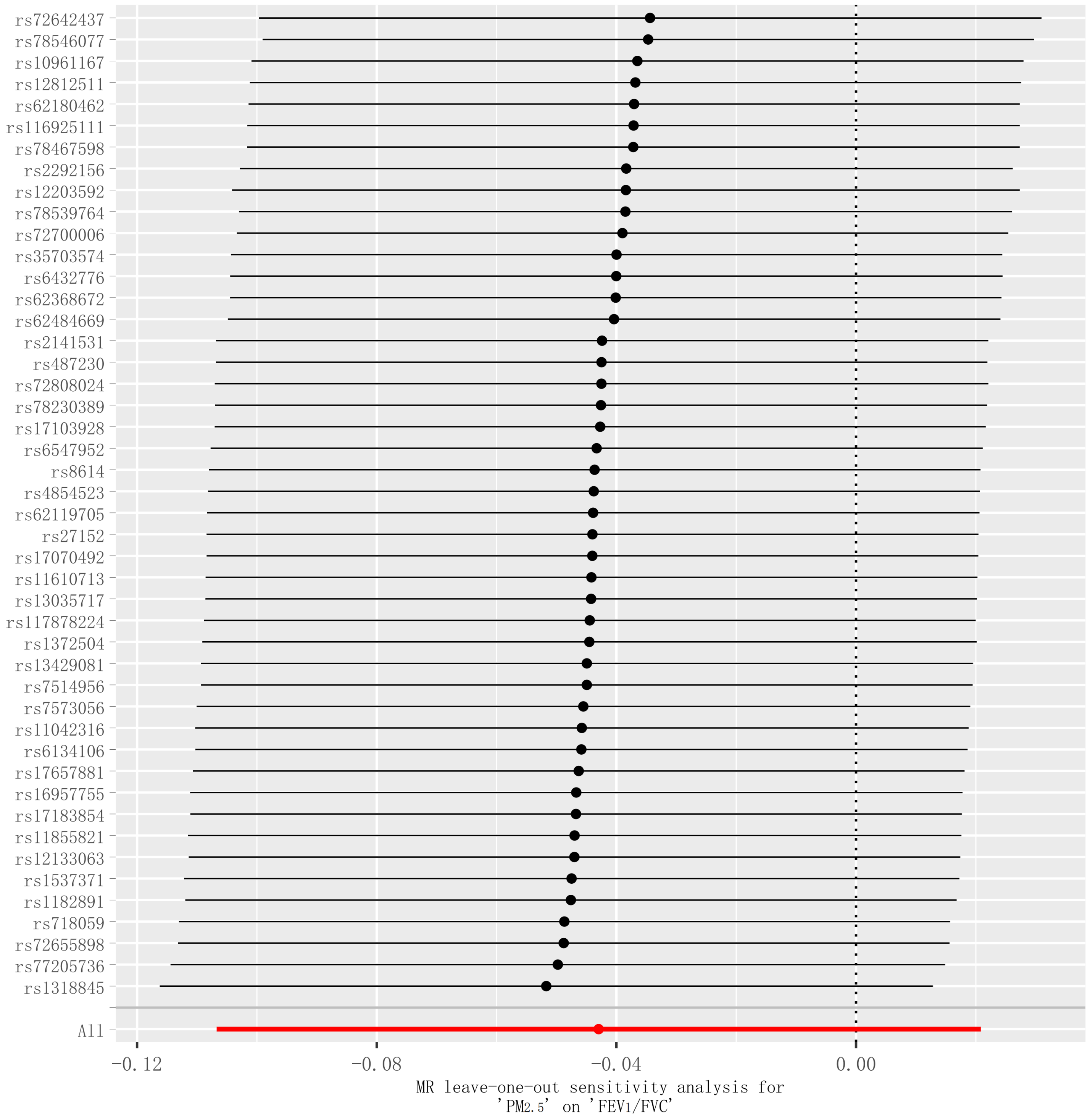

rs72642437
rs78546077
rs10961167
rs12812511
rs62180462
rs116925111
rs78467598
rs2292156
rs12203592
rs78539764
rs72700006
rs35703574
rs6432776
rs62368672
rs62484669
rs2141531
rs487230
rs72808024
rs78230389
rs17103928
rs6547952
rs8614
rs4854523
rs62119705
rs27152
rs17070492
rs11610713
rs13035717
rs117878224
rs1372504
rs13429081
rs7514956
rs7573056
rs11042316
rs6134106
rs17657881
rs16957755
rs17183854
rs11855821
rs12133063
rs1537371
rs1182891
rs718059
rs72655898
rs77205736
rs1318845
All
−0.12
−0.08
−0.04
0.00
MR leave-one-out sensitivity analysis for
'PM2.5' on 'FEV1/FVC'

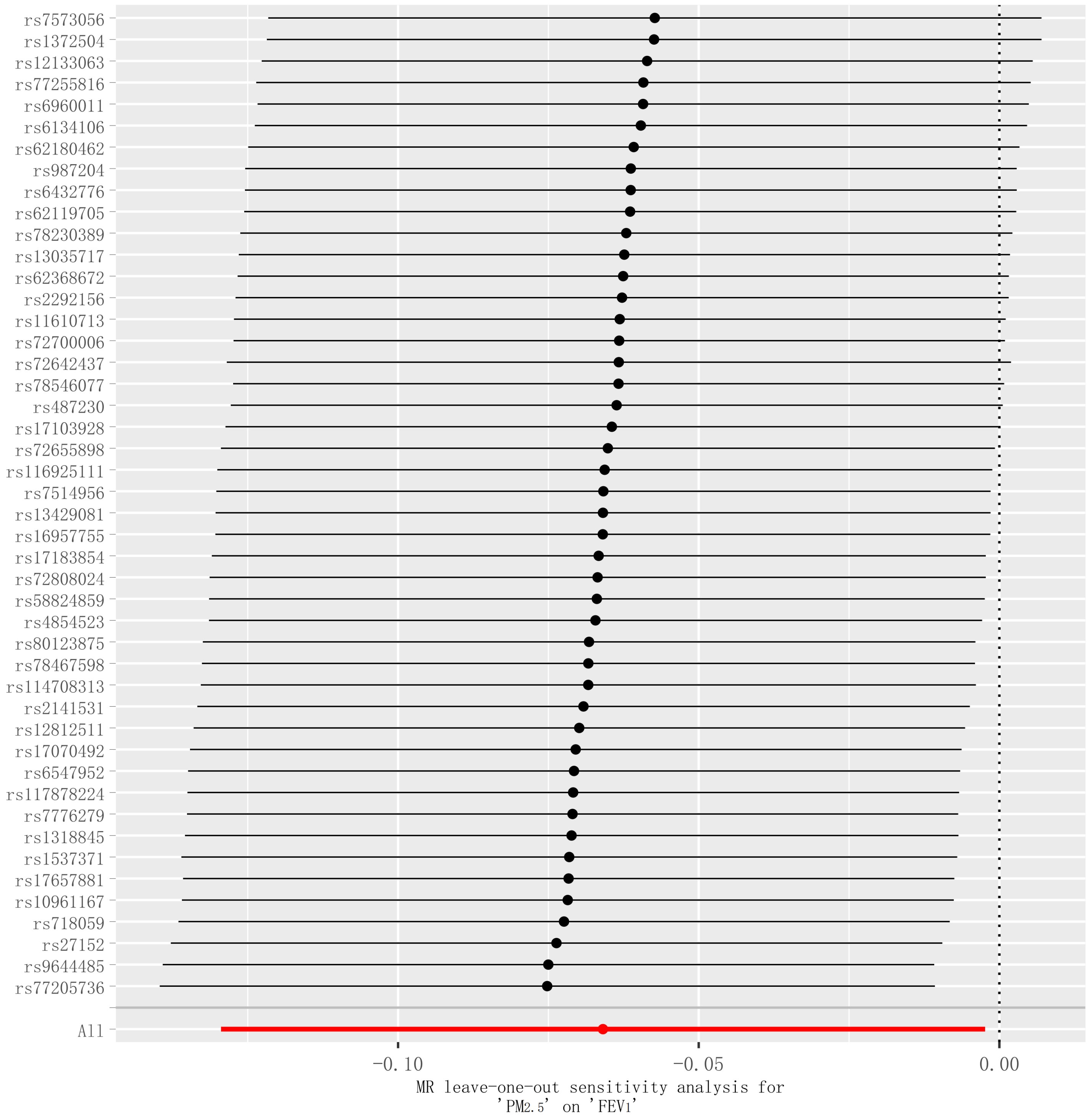
rs7573056
rs1372504
rs12133063
rs77255816
rs6960011
rs6134106
rs62180462
rs987204
rs6432776
rs62119705
rs78230389
rs13035717
rs62368672
rs2292156
rs11610713
rs72700006
rs72642437
rs78546077
rs487230
rs17103928
rs72655898
rs116925111
rs7514956
rs13429081
rs16957755
rs17183854
rs72808024
rs58824859
rs4854523
rs80123875
rs78467598
rs114708313
rs2141531
rs12812511
rs17070492
rs6547952
rs117878224
rs7776279
rs1318845
rs1537371
rs17657881
rs10961167
rs718059
rs27152
rs9644485
rs77205736
All
−0.10
−0.05
0.00
MR leave-one-out sensitivity analysis for
'PM2.5' on 'FEV1'

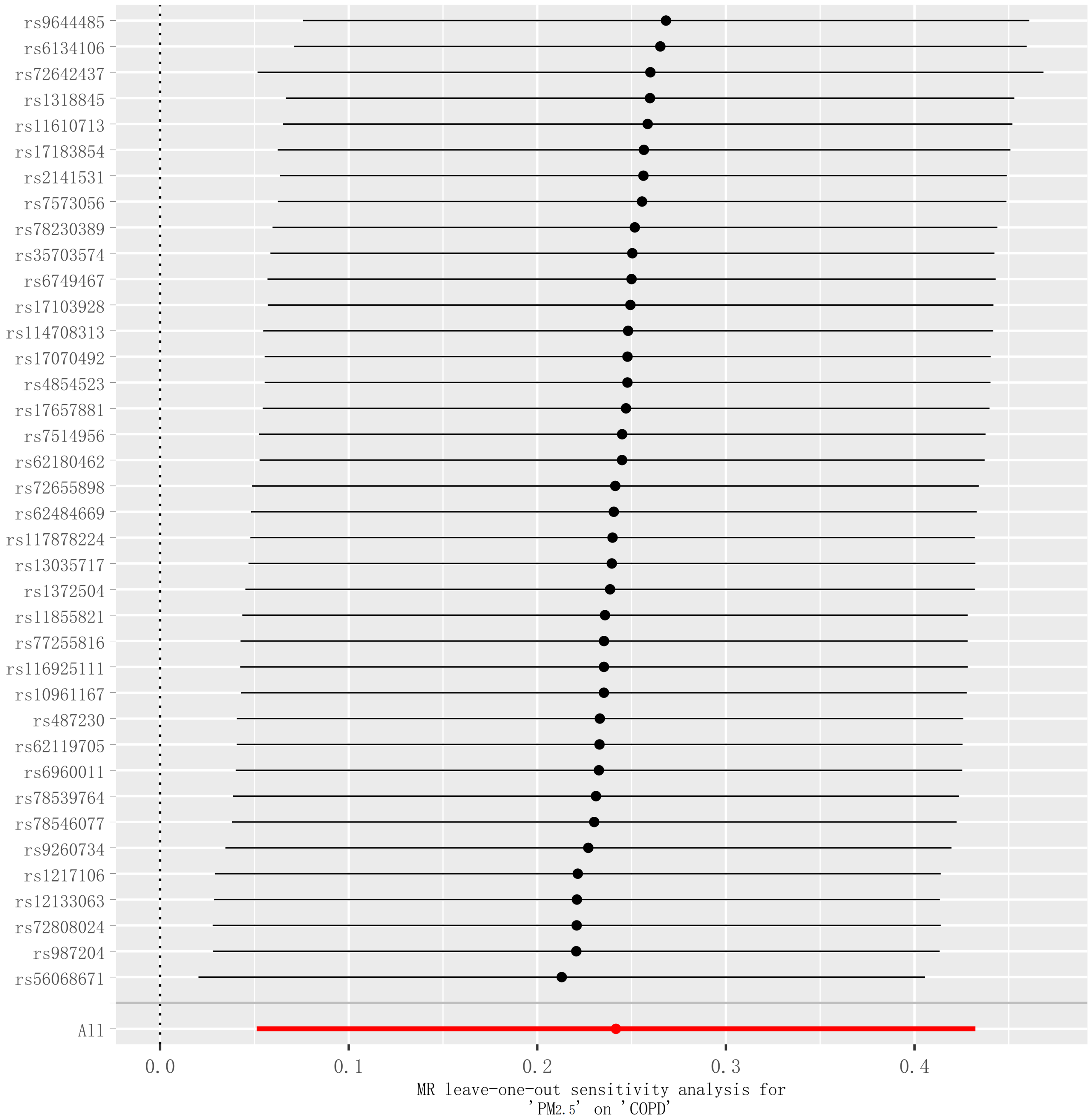

rs9644485
rs6134106
rs72642437
rs1318845
rs11610713
rs17183854
rs2141531
rs7573056
rs78230389
rs35703574
rs6749467
rs17103928
rs114708313
rs17070492
rs4854523
rs17657881
rs7514956
rs62180462
rs72655898
rs62484669
rs117878224
rs13035717
rs1372504
rs11855821
rs77255816
rs116925111
rs10961167
rs487230
rs62119705
rs6960011
rs78539764
rs78546077
rs9260734
rs1217106
rs12133063
rs72808024
rs987204
rs56068671
All
0.0
0.1
0.2
0.3
0.4
MR leave-one-out sensitivity analysis for
'PM2.5' on 'COPD'

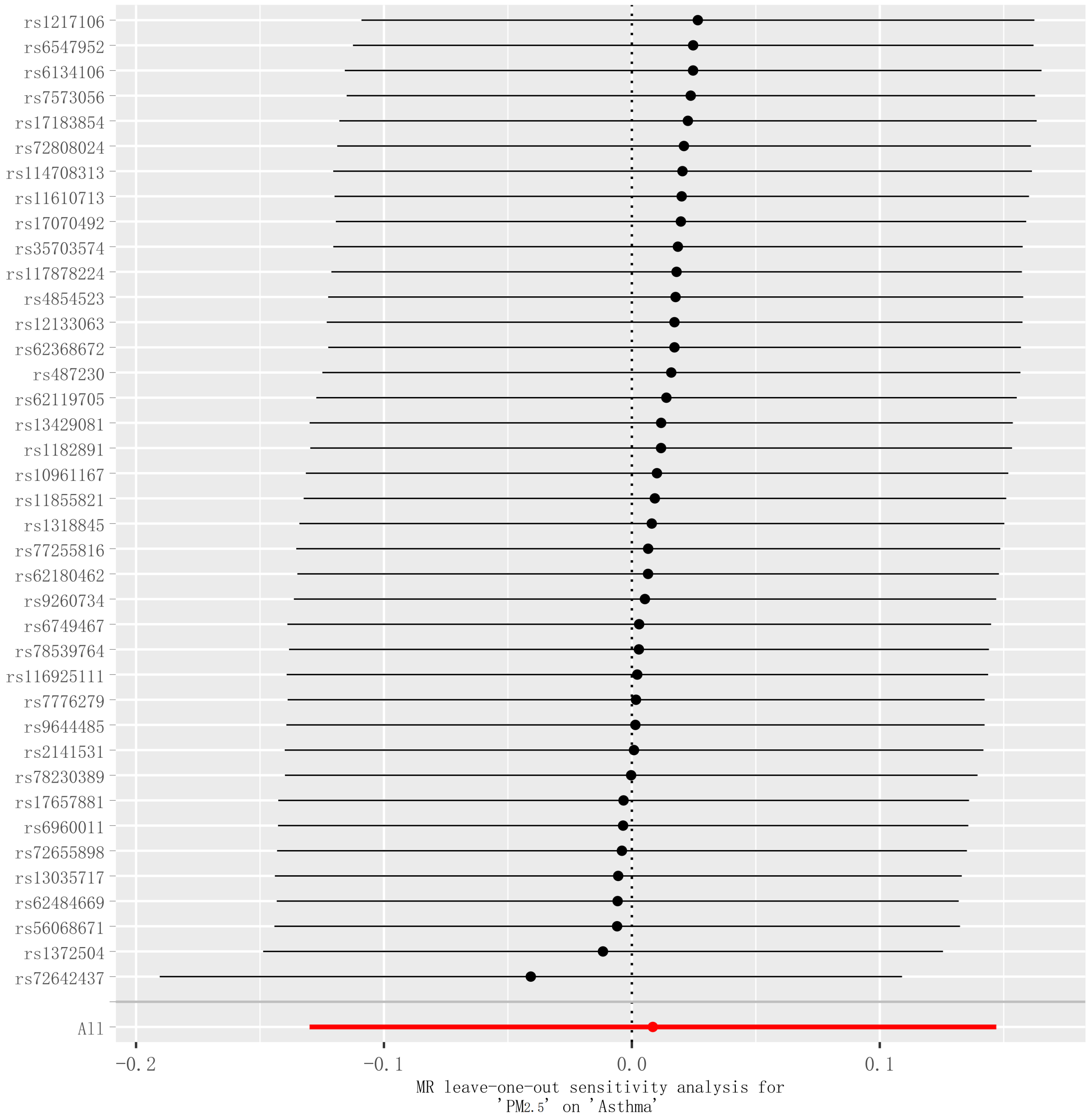
rs1217106
rs6547952
rs6134106
rs7573056
rs17183854
rs72808024
rs114708313
rs11610713
rs17070492
rs35703574
rs117878224
rs4854523
rs12133063
rs62368672
rs487230
rs62119705
rs13429081
rs1182891
rs10961167
rs11855821
rs1318845
rs77255816
rs62180462
rs9260734
rs6749467
rs78539764
rs116925111
rs7776279
rs9644485
rs2141531
rs78230389
rs17657881
rs6960011
rs72655898
rs13035717
rs62484669
rs56068671
rs1372504
rs72642437
All
-0.2
-0.1
0.0
0.1
MR leave-one-out sensitivity analysis for
'PM2.5' on 'Asthma'

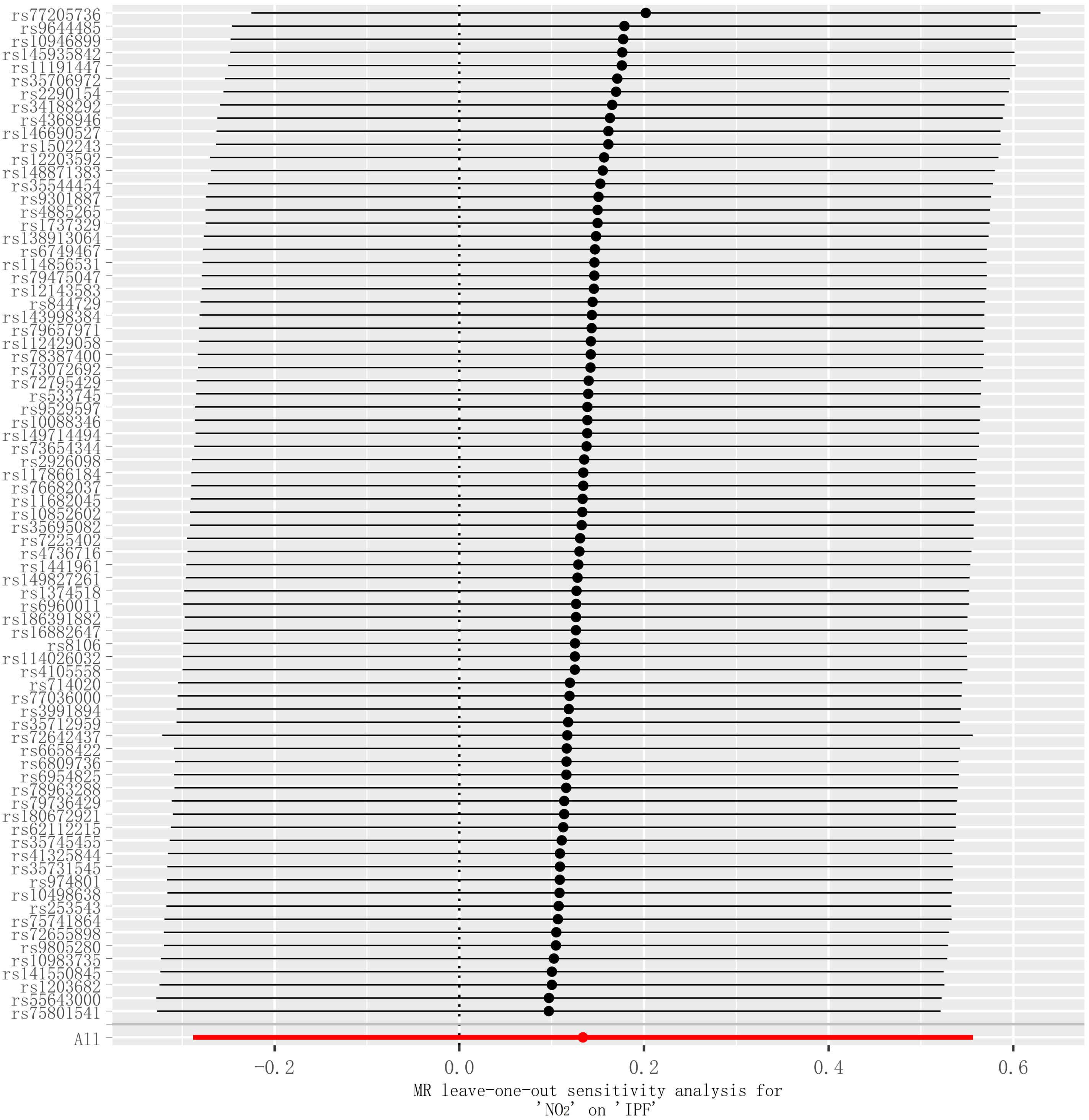
rs77205736
rs9644485
rs10946899
rs145935842
rs11191447
rs35706972
rs2290154
rs34188292
rs4368946
rs146690527
rs1502243
rs12203592
rs148871383
rs35544454
rs9301887
rs4885265
rs1737329
rs138913064
rs6749467
rs114856531
rs79475047
rs12143583
rs844729
rs143998384
rs79657971
rs112429058
rs78387400
rs73072692
rs72795429
rs533745
rs9529597
rs10088346
rs149714494
rs73654344
rs2926098
rs117866184
rs76682037
rs11682045
rs10852602
rs35695082
rs7225402
rs4736716
rs1441961
rs149827261
rs1374518
rs6960011
rs186391882
rs16882647
rs8106
rs114026032
rs4105558
rs714020
rs77036000
rs3991894
rs35712959
rs72642437
rs6658422
rs6809736
rs6954825
rs78963288
rs79736429
rs180672921
rs62112215
rs35745455
rs41325844
rs35731545
rs974801
rs10498638
rs253543
rs75741864
rs72655898
rs9805280
rs10983735
rs141550845
rs1203682
rs55643000
rs75801541
All
-0.2
0.0
0.2
0.4
0.6
MR leave-one-out sensitivity analysis for
'NO2' on 'IPF'

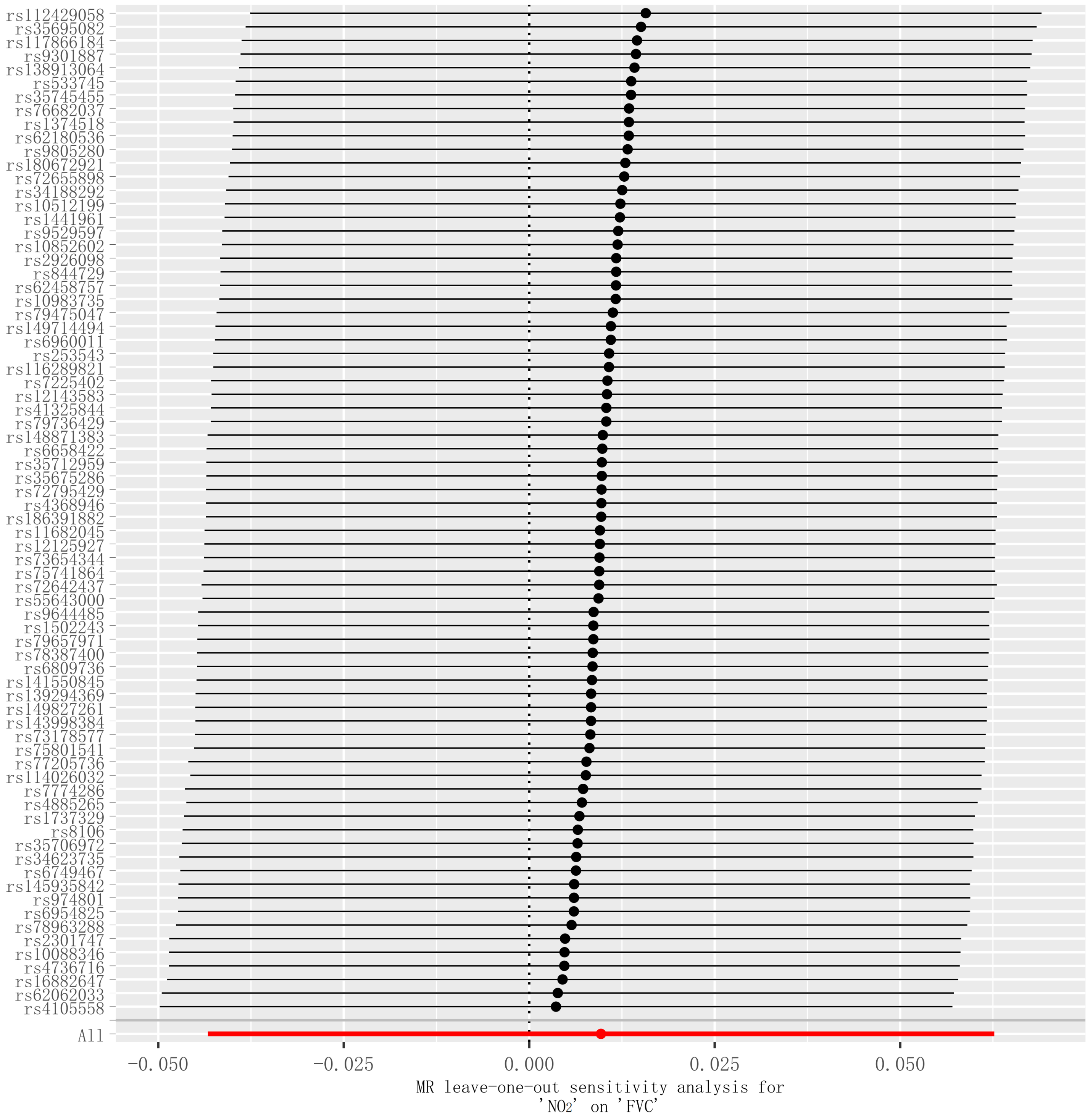
rs112429058
rs35695082
rs117866184
rs9301887
rs138913064
rs533745
rs35745455
rs76682037
rs1374518
rs62180536
rs9805280
rs180672921
rs72655898
rs34188292
rs10512199
rs1441961
rs9529597
rs10852602
rs2926098
rs844729
rs62458757
rs10983735
rs79475047
rs149714494
rs6960011
rs253543
rs116289821
rs7225402
rs12143583
rs41325844
rs79736429
rs148871383
rs6658422
rs35712959
rs35675286
rs72795429
rs4368946
rs186391882
rs11682045
rs12125927
rs73654344
rs75741864
rs72642437
rs55643000
rs9644485
rs1502243
rs79657971
rs78387400
rs6809736
rs141550845
rs139294369
rs149827261
rs143998384
rs73178577
rs75801541
rs77205736
rs114026032
rs7774286
rs4885265
rs1737329
rs8106
rs35706972
rs34623735
rs6749467
rs145935842
rs974801
rs6954825
rs78963288
rs2301747
rs10088346
rs4736716
rs16882647
rs62062033
rs4105558
All
−0.050
−0.025
0.000
0.025
0.050
MR leave-one-out sensitivity analysis for
'NO2' on 'FVC'

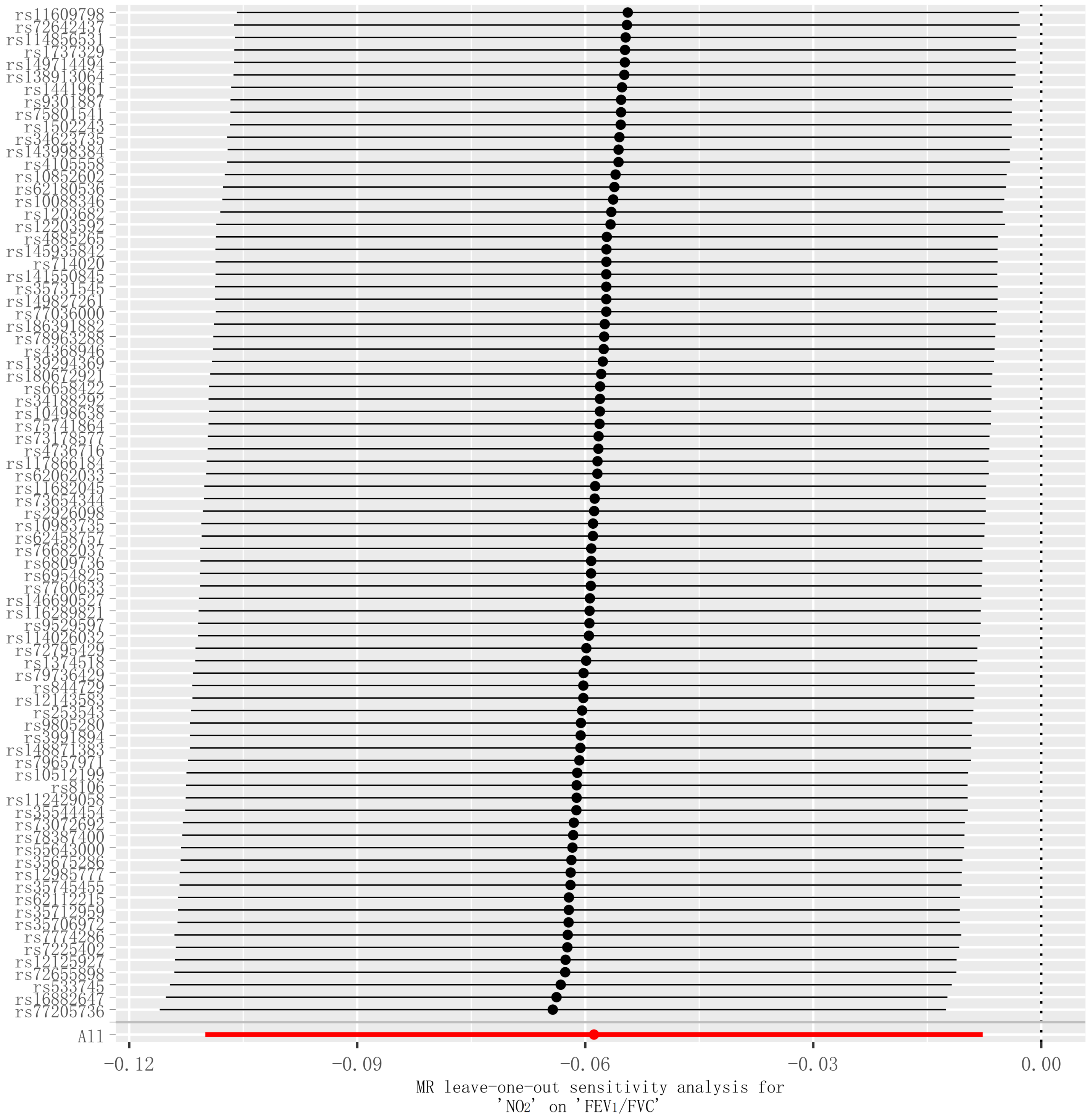
rs11609798
rs72642437
rs114856531
rs1737329
rs149714494
rs138913064
rs1441961
rs9301887
rs75801541
rs1502243
rs34623735
rs143998384
rs4105558
rs10852602
rs62180536
rs10088346
rs1203682
rs12203592
rs4885265
rs145935842
rs714020
rs141550845
rs35731545
rs149827261
rs77036000
rs186391882
rs78963288
rs4368946
rs139294369
rs180672921
rs6658422
rs34188292
rs10498638
rs75741864
rs73178577
rs4736716
rs117866184
rs62062033
rs11682045
rs73654344
rs2926098
rs10983735
rs62458757
rs76682037
rs6809736
rs6954825
rs7760633
rs146690327
rs116289821
rs9529597
rs114026032
rs72795429
rs1374518
rs79736429
rs844729
rs12143583
rs253543
rs9805280
rs3991894
rs148871383
rs79657971
rs10512199
rs8106
rs112429058
rs35544454
rs73072692
rs78387400
rs55643000
rs35675286
rs12985777
rs35745455
rs62112215
rs35712959
rs35706972
rs7774286
rs7225402
rs12125927
rs72655898
rs533745
rs16882647
rs77205736
All
-0.12
-0.09
-0.06
-0.03
0.00
MR leave-one-out sensitivity analysis for
'NO2' on 'FEV1/FVC'

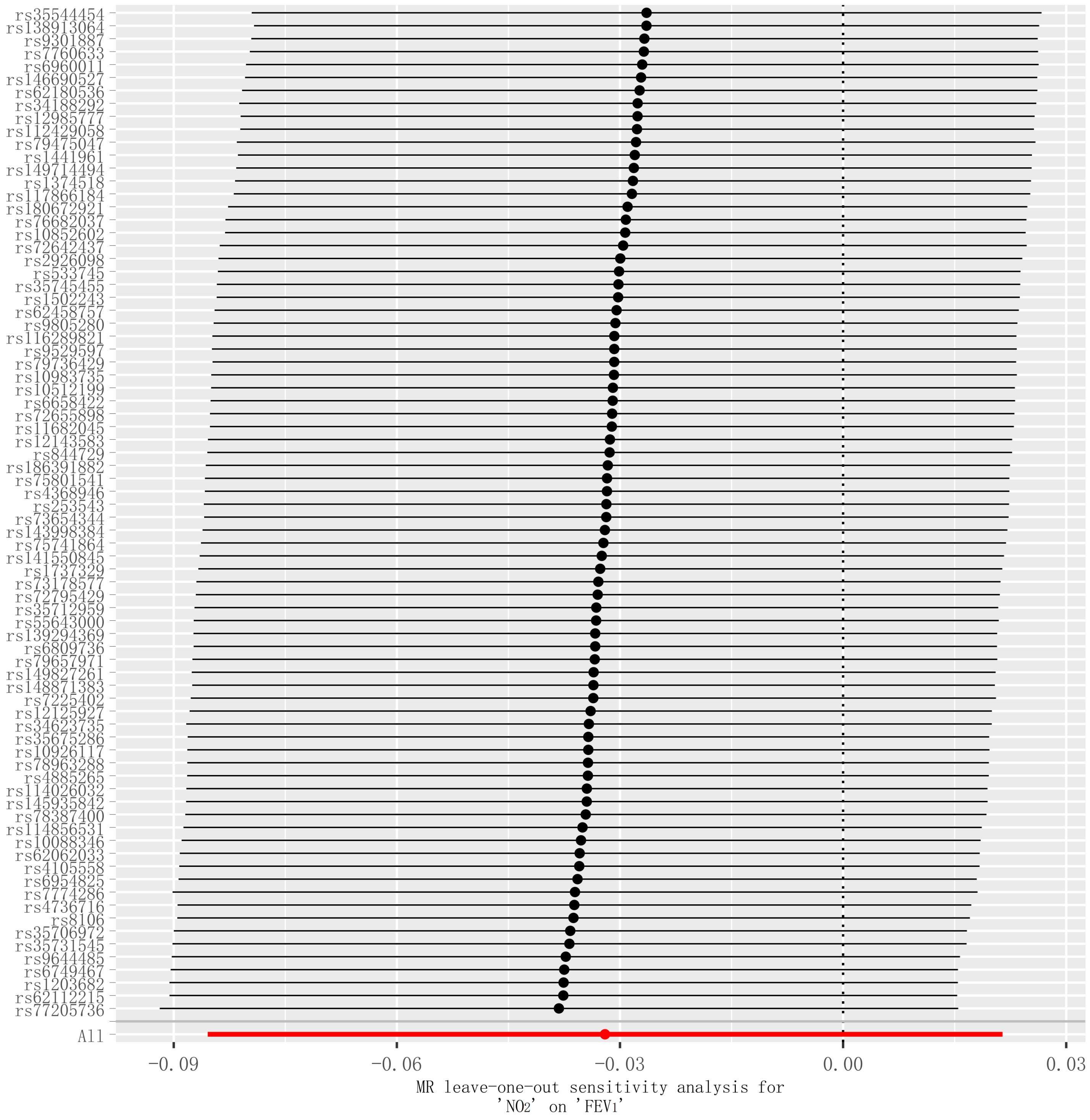
rs35544454
rs138913064
rs9301887
rs7760633
rs6960011
rs146690527
rs62180536
rs34188292
rs12985777
rs112429058
rs79475047
rs1441961
rs149714494
rs1374518
rs117866184
rs180672921
rs76682037
rs10852602
rs72642437
rs2926098
rs533745
rs35745455
rs1502243
rs62458757
rs9805280
rs116289821
rs9529597
rs79736429
rs10983735
rs10512199
rs6658422
rs72655898
rs11682045
rs12143583
rs844729
rs186391882
rs75801541
rs4368946
rs253543
rs73654344
rs143998384
rs75741864
rs141550845
rs1737329
rs73178577
rs72795429
rs35712959
rs55643000
rs139294369
rs6809736
rs79657971
rs149827261
rs148871383
rs7225402
rs12125927
rs34623735
rs35675286
rs10926117
rs78963288
rs4885265
rs114026032
rs145935842
rs78387400
rs114856531
rs10088346
rs62062033
rs4105558
rs6954825
rs7774286
rs4736716
rs8106
rs35706972
rs35731545
rs9644485
rs6749467
rs1203682
rs62112215
rs77205736
All
-0.09
-0.06
-0.03
0.00
0.03
MR leave-one-out sensitivity analysis for
'NO2' on 'FEV1'

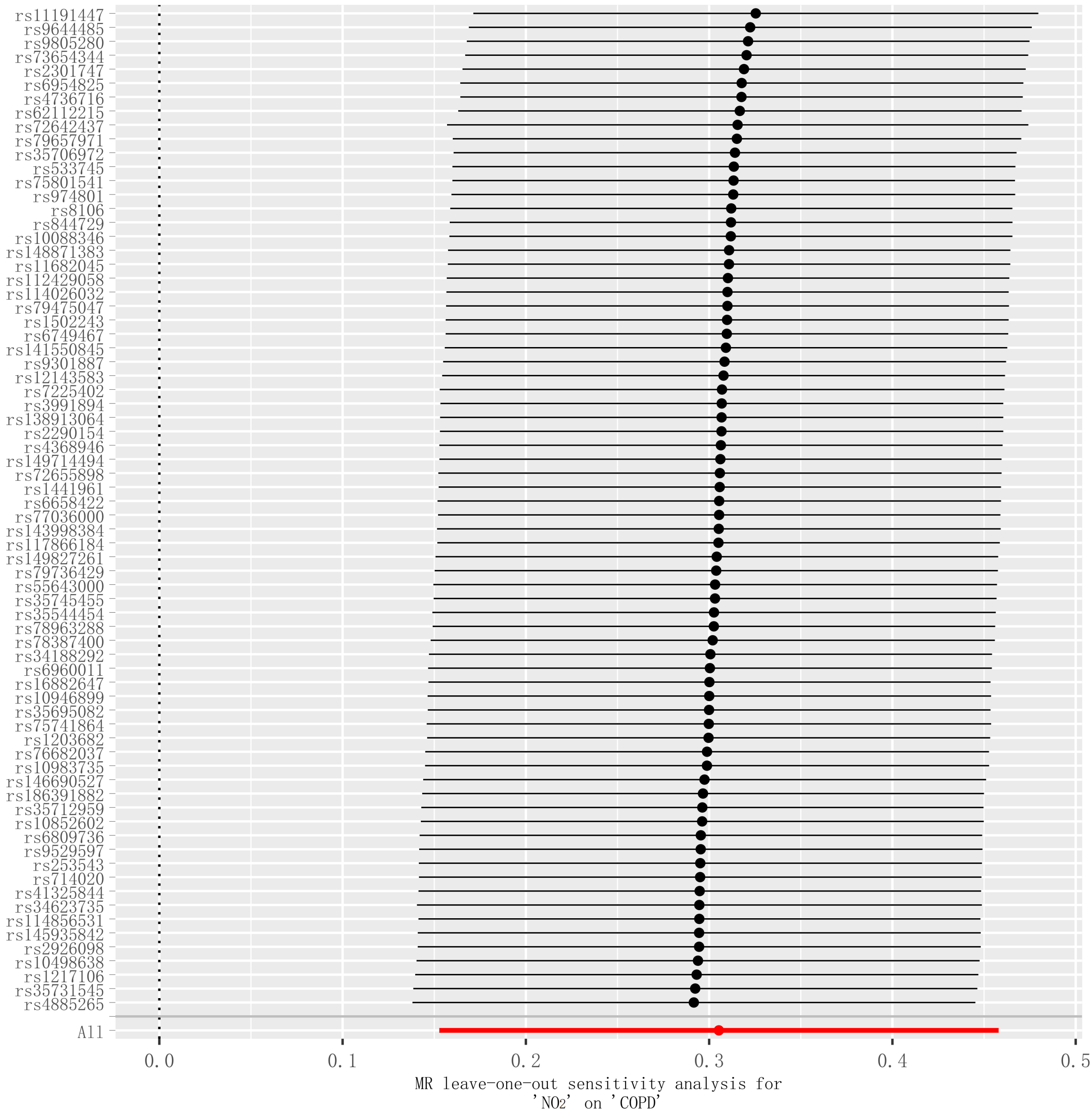

rs11191447
rs9644485
rs9805280
rs73654344
rs2301747
rs6954825
rs4736716
rs62112215
rs72642437
rs79657971
rs35706972
rs533745
rs75801541
rs974801
rs8106
rs844729
rs10088346
rs148871383
rs11682045
rs112429058
rs114026032
rs79475047
rs1502243
rs6749467
rs141550845
rs9301887
rs12143583
rs7225402
rs3991894
rs138913064
rs2290154
rs4368946
rs149714494
rs72655898
rs1441961
rs6658422
rs77036000
rs143998384
rs117866184
rs149827261
rs79736429
rs55643000
rs35745455
rs35544454
rs78963288
rs78387400
rs34188292
rs6960011
rs16882647
rs10946899
rs35695082
rs75741864
rs1203682
rs76682037
rs10983735
rs146690527
rs186391882
rs35712959
rs10852602
rs6809736
rs9529597
rs253543
rs714020
rs41325844
rs34623735
rs114856531
rs145935842
rs2926098
rs10498638
rs1217106
rs35731545
rs4885265
All
0.0
0.1
0.2
0.3
0.4
0.5
MR leave-one-out sensitivity analysis for
'NO2' on 'COPD'

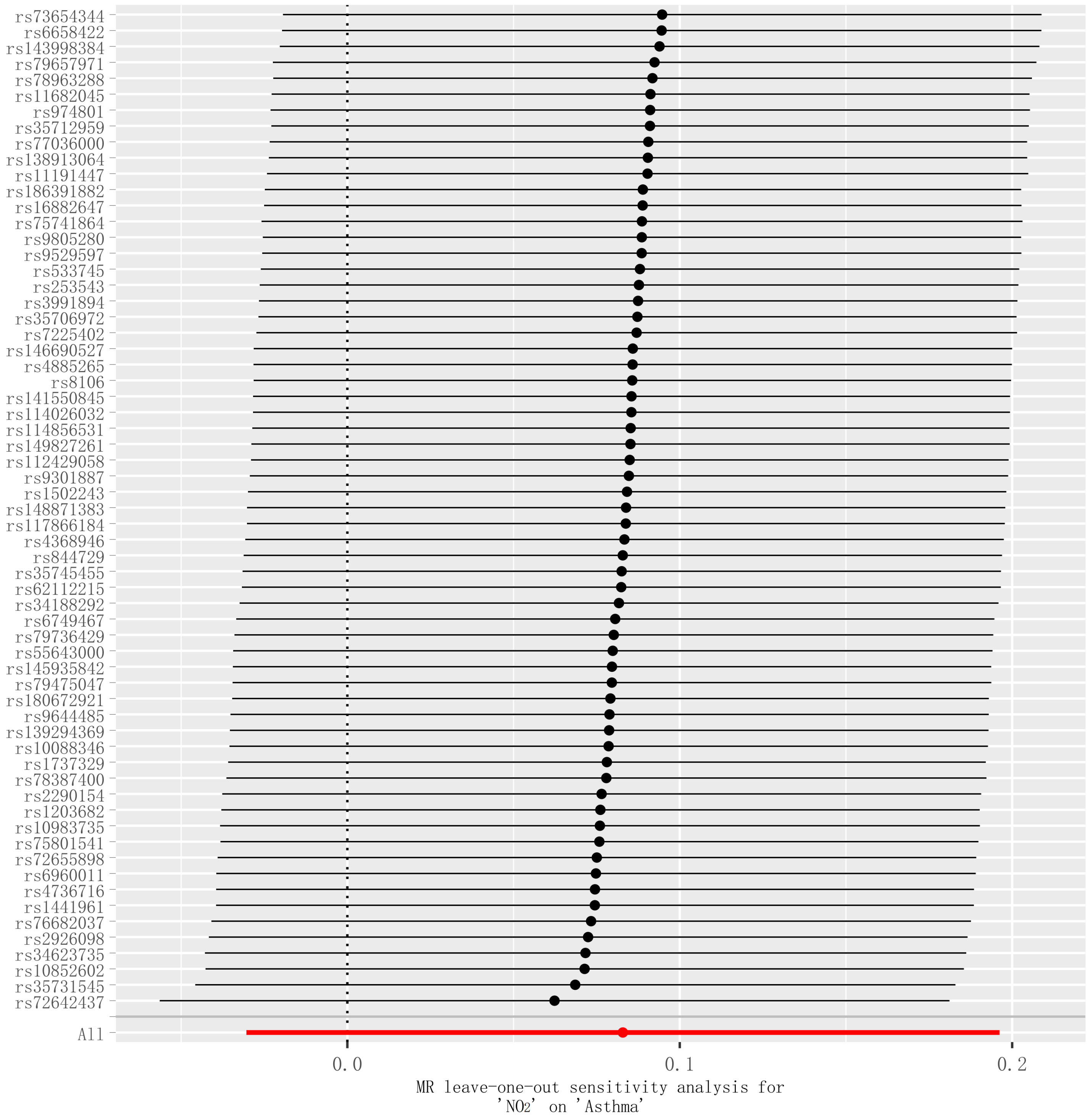

rs73654344
rs6658422
rs143998384
rs79657971
rs78963288
rs11682045
rs974801
rs35712959
rs77036000
rs138913064
rs11191447
rs186391882
rs16882647
rs75741864
rs9805280
rs9529597
rs533745
rs253543
rs3991894
rs35706972
rs7225402
rs146690527
rs4885265
rs8106
rs141550845
rs114026032
rs114856531
rs149827261
rs112429058
rs9301887
rs1502243
rs148871383
rs117866184
rs4368946
rs844729
rs35745455
rs62112215
rs34188292
rs6749467
rs79736429
rs55643000
rs145935842
rs79475047
rs180672921
rs9644485
rs139294369
rs10088346
rs1737329
rs78387400
rs2290154
rs1203682
rs10983735
rs75801541
rs72655898
rs6960011
rs4736716
rs1441961
rs76682037
rs2926098
rs34623735
rs10852602
rs35731545
rs72642437
All
0.0
0.1
0.2
MR leave-one-out sensitivity analysis for
'NO2' on 'Asthma'

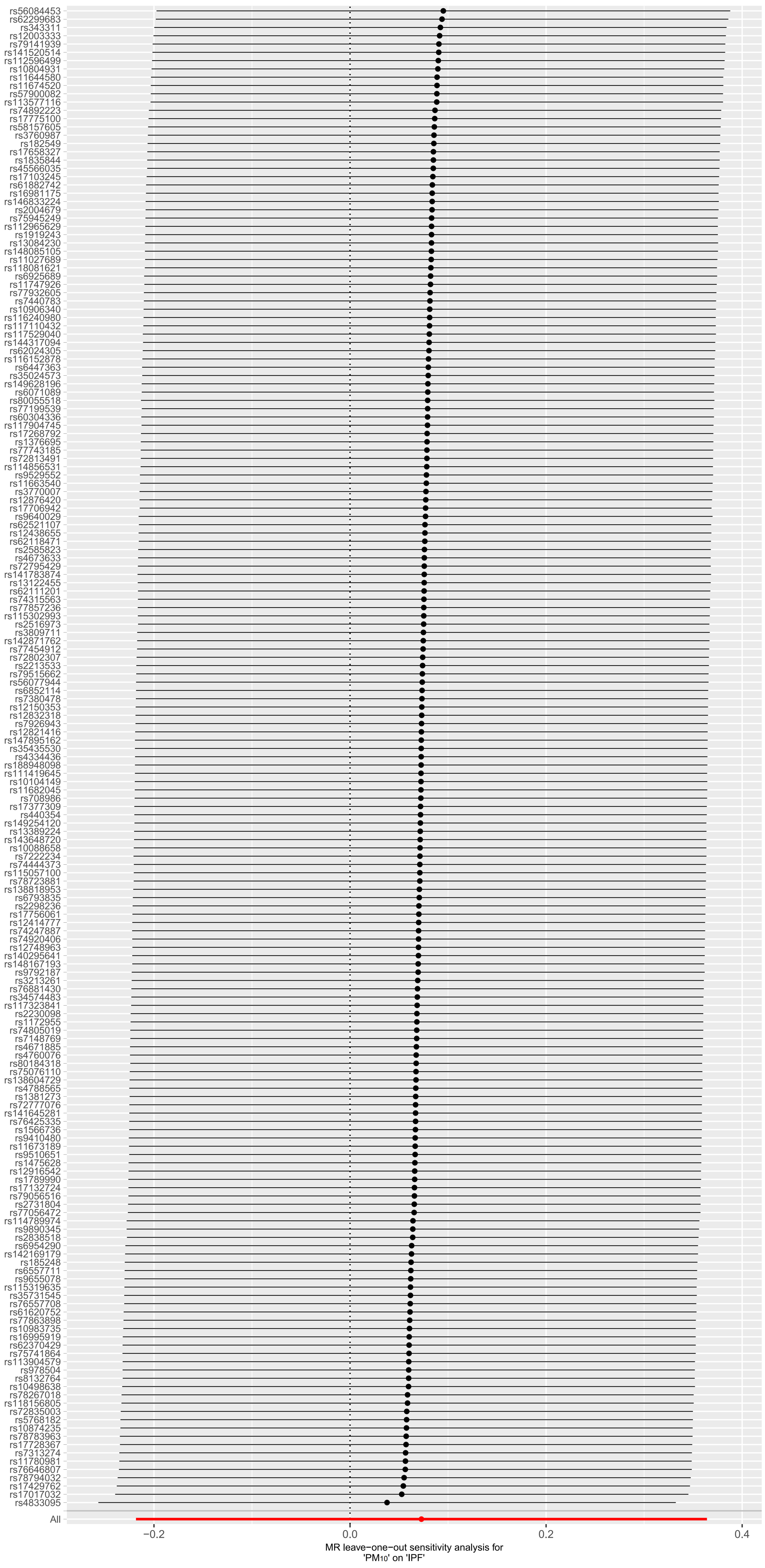
rs56084453
rs62299683
rs343311
rs12003333
rs79141939
rs141520514
rs112596499
rs10804931
rs11644580
rs11674520
rs57900082
rs113577116
rs74892223
rs17775100
rs58157605
rs3760987
rs182549
rs17658327
rs1835844
rs45566035
rs17103245
rs61882742
rs16981175
rs146833224
rs2004679
rs75945249
rs112965629
rs1919243
rs13084230
rs148085105
rs11027689
rs118081621
rs6925689
rs11747926
rs77932605
rs7440783
rs10906340
rs116240980
rs117110432
rs117529040
rs144317094
rs62024305
rs116152878
rs6447363
rs35024573
rs149628196
rs6071089
rs80055518
rs77199539
rs60304336
rs117904745
rs17268792
rs1376695
rs77743185
rs72813491
rs114856531
rs9529552
rs11663540
rs3770007
rs12876420
rs17706942
rs9640029
rs62521107
rs12438655
rs62118471
rs2585823
rs4673633
rs72795429
rs141783874
rs13122455
rs62111201
rs74315563
rs77857236
rs115302993
rs2516973
rs3809711
rs142871762
rs77454912
rs72802307
rs2213533
rs79515662
rs56077944
rs6852114
rs7380478
rs12150353
rs12832318
rs7926943
rs12821416
rs147895162
rs35435530
rs4334436
rs188948098
rs111419645
rs10104149
rs11682045
rs708986
rs17377309
rs440354
rs149254120
rs13389224
rs143648720
rs10088658
rs7222234
rs74444373
rs115057100
rs78723881
rs138818953
rs6793835
rs2298236
rs17756061
rs12414777
rs74247887
rs74920406
rs12748963
rs140295641
rs148167193
rs9792187
rs3213261
rs76881430
rs34574483
rs117323841
rs2230098
rs1172955
rs74805019
rs7148769
rs4671885
rs4760076
rs80184318
rs75076110
rs138604729
rs4788565
rs1381273
rs72777076
rs141645281
rs76425335
rs1566736
rs9410480
rs11673189
rs9510651
rs1475628
rs12916542
rs1789990
rs17132724
rs79056516
rs2731804
rs77056472
rs114789974
rs9890345
rs2838518
rs6954290
rs142169179
rs185248
rs6557711
rs9655078
rs115319635
rs35731545
rs76557708
rs61620752
rs77863898
rs10983735
rs16995919
rs62370429
rs75741864
rs113904579
rs978504
rs8132764
rs10498638
rs78267018
rs118156805
rs72835003
rs5768182
rs10874235
rs78783963
rs17728367
rs7313274
rs11780981
rs76646807
rs78794032
rs17429762
rs17017032
rs4833095
All
−0.2
0.0
0.2
0.4
MR leave−one−out sensitivity analysis for
'PM10' on 'IPF'

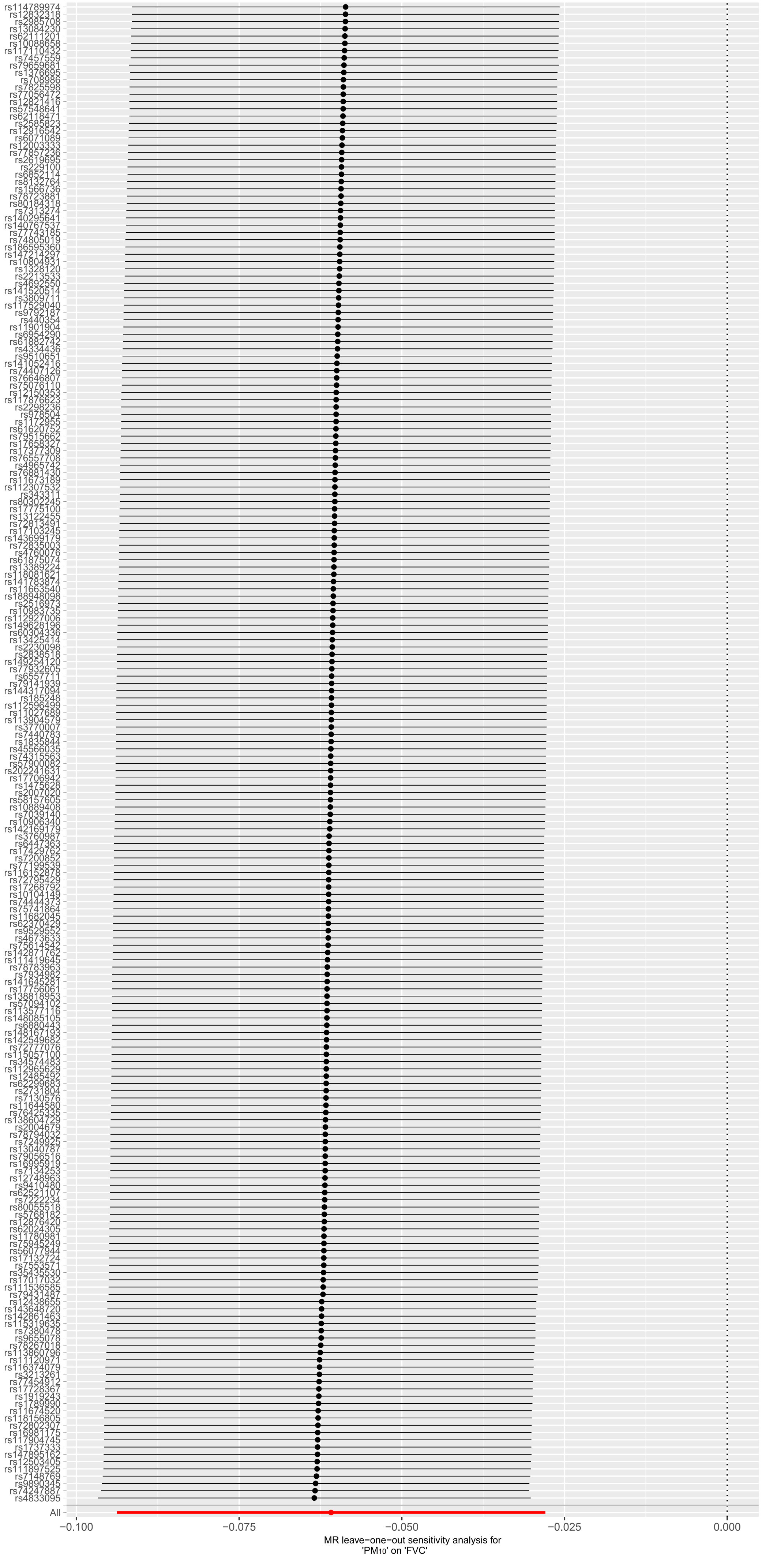

All
−0.100
−0.075
−0.050
−0.025
0.000
MR leave−one−out sensitivity analysis for
'PM10' on 'FVC'

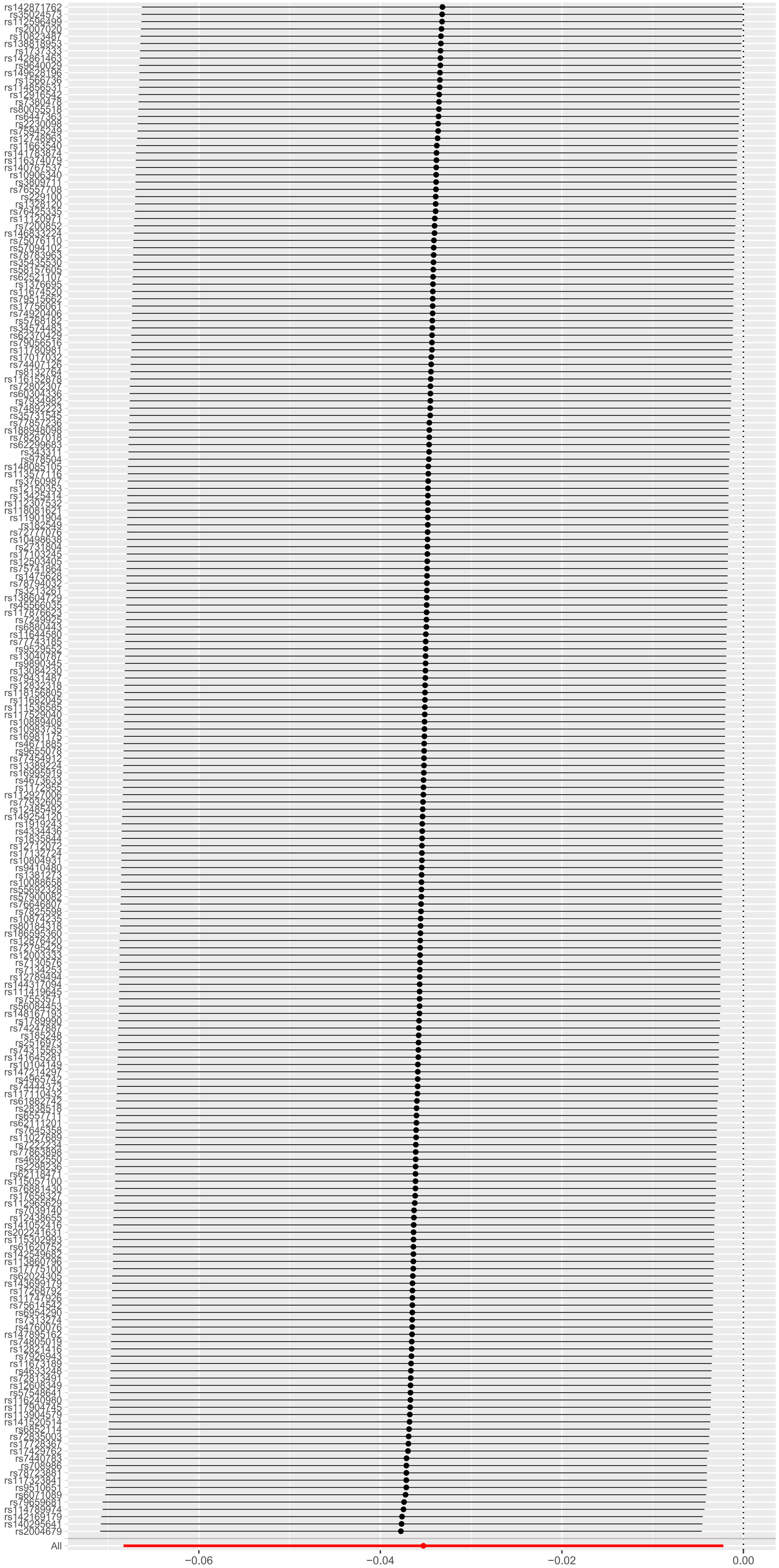
All
−0.06
−0.04
−0.02
0.00
MR leave−one−out sensitivity analysis for
'PM10' on 'FEV1/FVC'

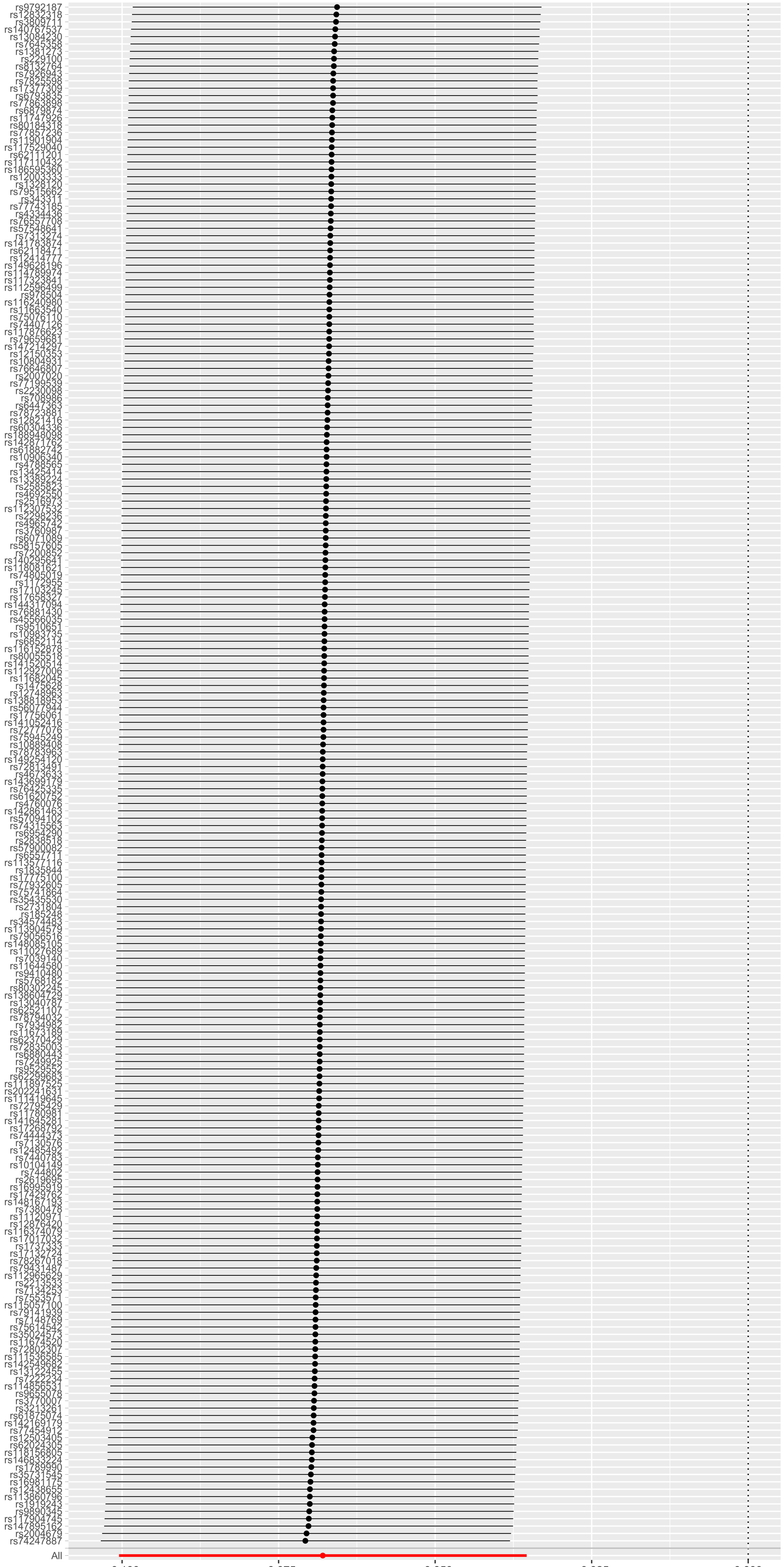
All
−0.100
−0.075
−0.050
−0.025
0.000
MR leave−one−out sensitivity analysis for
'PM10' on 'FEV1'

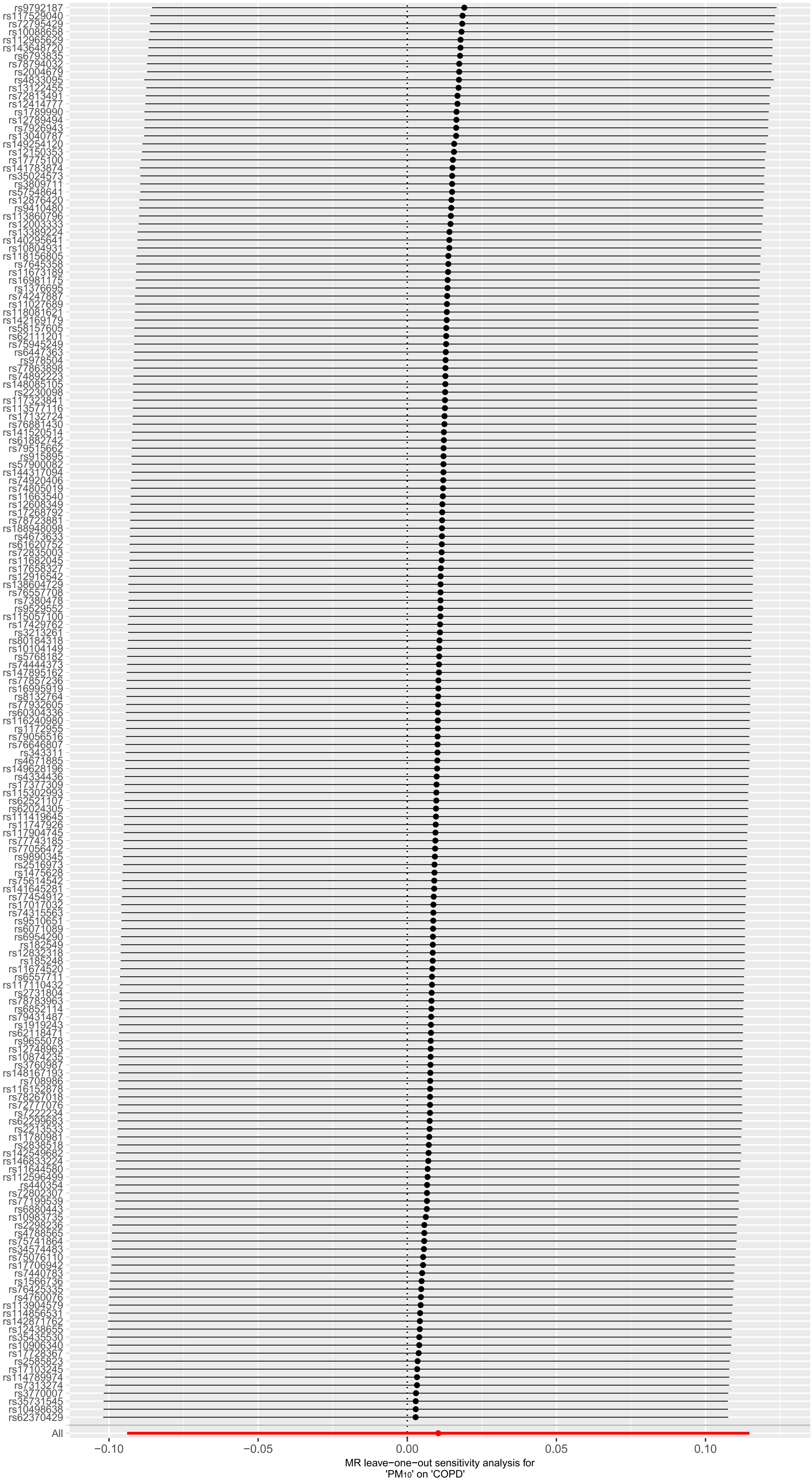
rs9792187
rs117529040
rs72795429
rs10088658
rs112965629
rs143648720
rs6793835
rs78794032
rs2004679
rs4833095
rs13122455
rs72813491
rs12414777
rs1789990
rs12789494
rs7926943
rs13040787
rs149254120
rs12150353
rs17775100
rs141783874
rs35024573
rs3809711
rs57548641
rs12876420
rs9410480
rs113860796
rs12003333
rs13389224
rs140295641
rs10804931
rs118156805
rs7645358
rs11673189
rs16981175
rs1376695
rs74247887
rs11027689
rs118081621
rs142169179
rs58157605
rs62111201
rs75945249
rs6447363
rs978504
rs77863898
rs74892223
rs148085105
rs2230098
rs117323841
rs113577116
rs17132724
rs76881430
rs141520514
rs61882742
rs79515662
rs915895
rs57900082
rs144317094
rs74920406
rs74805019
rs11663540
rs12608349
rs17268792
rs78723881
rs188948098
rs4673633
rs61620752
rs72835003
rs11682045
rs17658327
rs12916542
rs138604729
rs76557708
rs7380478
rs9529552
rs115057100
rs17429762
rs3213261
rs80184318
rs10104149
rs5768182
rs74444373
rs147895162
rs77857236
rs16995919
rs8132764
rs77932605
rs60304336
rs116240980
rs1172955
rs79056516
rs76646807
rs343311
rs4671885
rs149628196
rs4334436
rs17377309
rs115302993
rs62521107
rs62024305
rs111419645
rs11747926
rs117904745
rs77743185
rs77056472
rs9890345
rs2516973
rs1475628
rs75614542
rs141645281
rs77454912
rs17017032
rs74315563
rs9510651
rs6071089
rs6954290
rs182549
rs12832318
rs185248
rs11674520
rs6557711
rs117110432
rs2731804
rs78783963
rs6852114
rs79431487
rs1919243
rs62118471
rs9655078
rs12748963
rs10874235
rs3760987
rs148167193
rs708986
rs116152878
rs78267018
rs72777076
rs7222234
rs62299683
rs2213533
rs11780981
rs2838518
rs142549682
rs146833224
rs11644580
rs112596499
rs440354
rs72802307
rs77199539
rs6880443
rs10983735
rs2298236
rs4788565
rs75741864
rs34574483
rs75076110
rs17706942
rs7440783
rs1566736
rs76425335
rs4760076
rs113904579
rs114856531
rs142871762
rs12438655
rs35435530
rs10906340
rs17728367
rs2585823
rs17103245
rs114789974
rs7313274
rs3770007
rs35731545
rs10498638
rs62370429
All
−0.10
−0.05
0.00
0.05
0.10
MR leave−one−out sensitivity analysis for
'PM10' on 'COPD'

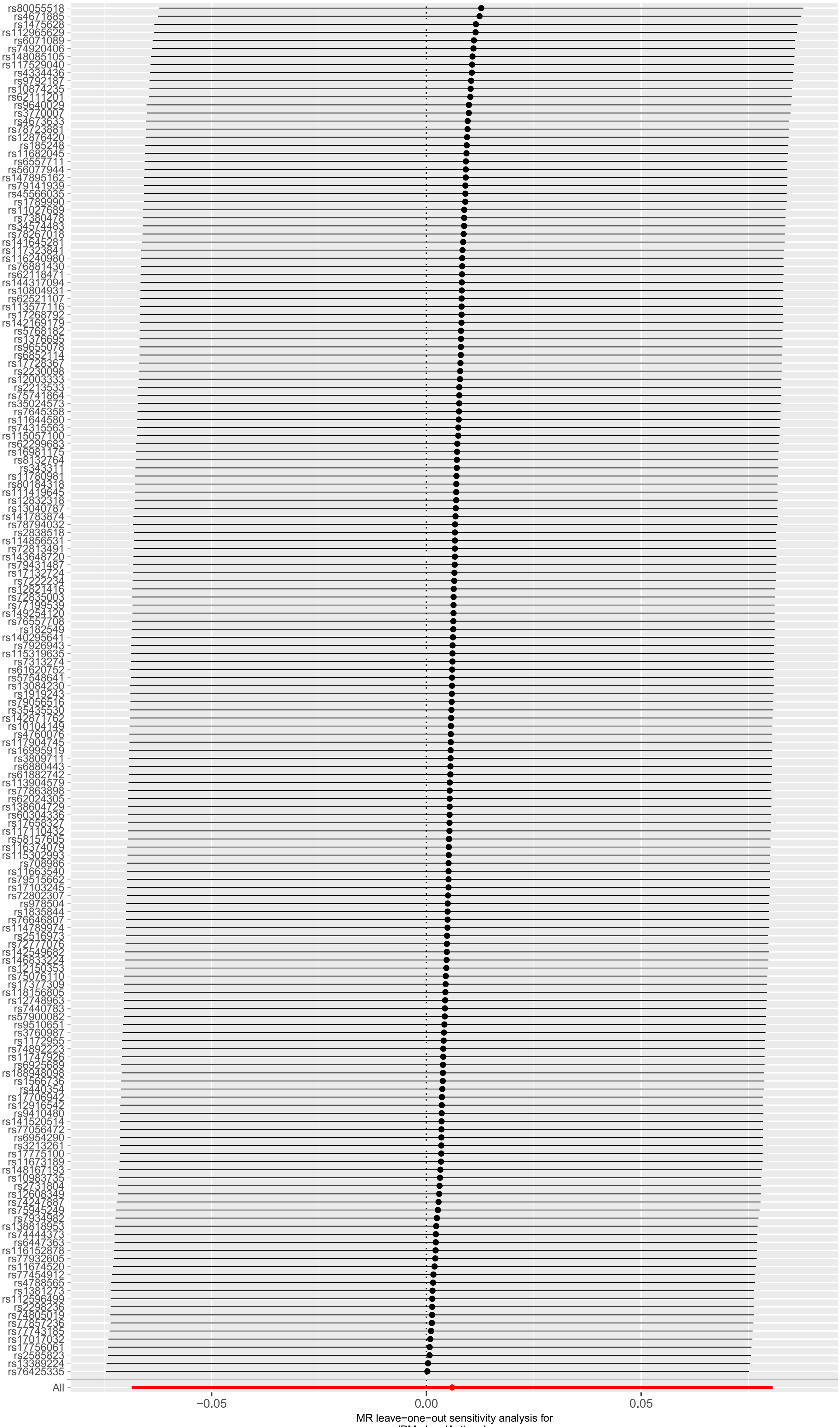
All
-0.05
0.00
0.05
MR leave-one-out sensitivity analysis for
'PM10' on 'Asthma'
